# Global, regional, and national burden of unintentional childhood poisoning, 1990–2021: an analysis of data from the Global Burden of Disease study 2021

**DOI:** 10.3389/fpubh.2025.1596599

**Published:** 2025-08-05

**Authors:** Wenqian Wang, Ding Yuan, Fang Yang, Yanxia Gao

**Affiliations:** Emergency Department, The First Affiliated Hospital of Zhengzhou University, Zhengzhou, China

**Keywords:** poisoning, children, GBD 2021, incidence, DALYs

## Abstract

**Introduction:**

Childhood poisoning remains a significant global public health challenge, driving ongoing efforts to reduce its effect across countries and regions. However, most previous studies have been limited to individual medical centers or specific geographic areas, lacking a comprehensive global comparison. Therefore, this study aims to utilize the Global Burden of Disease (GBD) database to assess the burden of childhood poisoning among children under 14 years old at the global, regional, and national levels from 1990 to 2021.

**Methods:**

Secondary analysis using data from the 2021 GBD database was conducted to assess the burden and temporal trends of childhood poisoning from 1990 to 2021. Health inequality was evaluated using the Slope Index of Inequality and the Concentration Index. Additionally, a frontier analysis was performed to identify regions with the potential for burden reduction.

**Results:**

Between 1990 and 2021, the global age-standardized incidence rate (ASIR), age-standardized mortality rate (ASMR), and age-standardized disability-adjusted life years rate (ASDR) for childhood poisoning declined. In 2021, the ASIR, ASMR, and ASDR for children under 14 years old per 100,000 population was 40.46 (95% UI: 24.87–63.29), 0.45 (95% UI: 0.28–0.65), and 40.2 (95% UI: 26.32–57.93). Across different SDI regions, high-SDI countries had the highest ASIR, while low-SDI countries had the highest ASMR and ASDR. Among the 204 countries analyzed, Norway had the highest ASIR in 2021, whereas South Sudan had the highest ASMR and ASDR. Health inequality analysis revealed that while the gap between low- and high-SDI countries narrowed over time, children in lower-SDI countries continued to experience a significantly higher disability burden. Frontier analysis revealed a negative correlation between ASDR and SDI; however, childhood poisoning burdens varied significantly among countries with similar SDI levels.

**Conclusion:**

Between 1990 and 2021, the global burden of childhood poisoning declined, yet health inequalities remain, with less-developed countries disproportionately affected. Future efforts should strengthen emergency response systems in these regions, promote poisoning prevention and safe storage regulations in higher-development countries, and further investigate childhood poisoning risk factors.

## 1 Introduction

Children have an underdeveloped cognitive awareness of risk, increasing their susceptibility to poisoning from exposure to medications, household chemicals, toxic plants, and other hazardous substances. Childhood poisoning is a significant global public health concern and is the fourth leading cause of unintentional death among children aged 0–14 years (1). Childhood poisoning imposes a significant economic burden, particularly in low-income regions, where medical costs can reach 60–300% of the local minimum monthly wage (2, 3). However, most childhood poisoning incidents are preventable, as parents or guardians largely control the living environment and safety conditions of a child. Over the years, global efforts have continued to reduce the burden of childhood poisoning (4). Nonetheless, variations in economic development and resource availability across countries influence poisoning rates and health outcomes. Assessing the global burden of childhood poisoning is essential for developing targeted prevention strategies across different socioeconomic contexts. However, most epidemiological studies on childhood poisoning focus on specific regions or healthcare facilities, lacking comprehensive cross-national comparisons.

Therefore, this study utilizes the 2021 Global Burden of Disease (GBD) database to conduct a secondary analysis, examining the epidemiological patterns of childhood poisoning worldwide and assessing the burden disparities and potential areas for improvement across regions and countries at different stages of socioeconomic development. Through these analyses, our study could provide a scientific basis for developing targeted poisoning prevention and public health policies, particularly for countries at different levels of socioeconomic development.

## 2 Materials and methods

In this study, we utilized data from the 2021 GBD database to collect information on the incidence, mortality, and disability-adjusted life years (DALYs) related to poisoning among children aged 0–14 years across 204 countries and territories from 1990 to 2021. The GBD study, a comprehensive global epidemiological research initiative, integrates data from multiple sources, including population-based surveys, hospital records, death registries, surveillance systems, and other health datasets (5). We specifically analyzed poisoning as defined by the GBD, which includes unintentional exposure to a noninfectious substance that contacts or enters the body via inhalation, ingestion, injection, or absorption, leading to physiological dysfunction or death. This definition aligns with the International Classification of Diseases (ICD) coding system (ICD-9: E856-E857.99, E860-E865, E867-E869.99; E929.2 ICD-10: J70.5, X46-X48.9). The diagnostic scope does not include adverse drug reactions, intentional self-harm, drug abuse, or assault. A detailed description of the ICD-coded diagnoses included in this definition is provided in the Supplementary Table 1.

Given the differences in developmental, behavioral, and exposure risk profiles across age groups, the study population was categorized into three age groups: under 5 years, 5–9 years, and 10–14 years. Countries were classified into five groups based on the Sociodemographic Index (SDI): high, high-middle, middle, low-middle, and low SDIs. The GBD study developed a composite index, the SDI, which is used to assess the socioeconomic development level of countries based on factors such as per capita income, educational attainment, and fertility rates (6). In addition, GBD 2021 estimates 204 countries and territories, which are organized into 21 GBD regions with close geographic proximity, similar epidemiology, and similar cause of death distribution (5).

We applied direct-standardization methods to calculate ASIR, ASMR, and ASDR. Age standardization was performed using the world standard population to eliminate the influence of population structure differences when comparing incidence, mortality, and DALYs across different populations. Direct standardization requires a reference population, which in this study is the 2021 global standard population estimated by GBD (7, 8). To analyze temporal trends in incidence, mortality, and DALYs, we calculated the Estimated Annual Percentage Change (EAPC). The EAPC was estimated using a linear regression model, with the log-transformed age-standardized rates (ASIR, ASMR, and ASDR) as the dependent variables and year as the independent variable. The regression coefficient was used to determine the trend in disease burden over time (9).

To assess health inequalities in childhood poisoning burden, we applied the Slope Index of Inequality (SII) and the Concentration Index (CI). SII was used to measure absolute inequality through the evaluation of the differences in disease burden between the highest and lowest socioeconomic groups, while CI was used to measure relative inequality through the assessment of disease burden concentration in lower-income populations (10). In order to evaluate the relationship between burden of poisoning and socio-demographic development, we applied a frontier analysis as a quantitative methodology to identify the lowest potentially achievable burden on the basis of development status as measured by the SDI. The frontier pinpoints the minimum burden that could be attained for every country or territory given its SDI. Distance from the frontier is termed effective difference (EF); a large EF from the frontier suggests there may be unrealized opportunities for gains or improvement that should be possible based on the country or territory’s place on the development spectrum (11). All statistical analyses and visualizations were conducted in R (version 4.4.1) and Stata (version 16.0).

## 3 Results

### 3.1 Global trends

**Figure 1:**
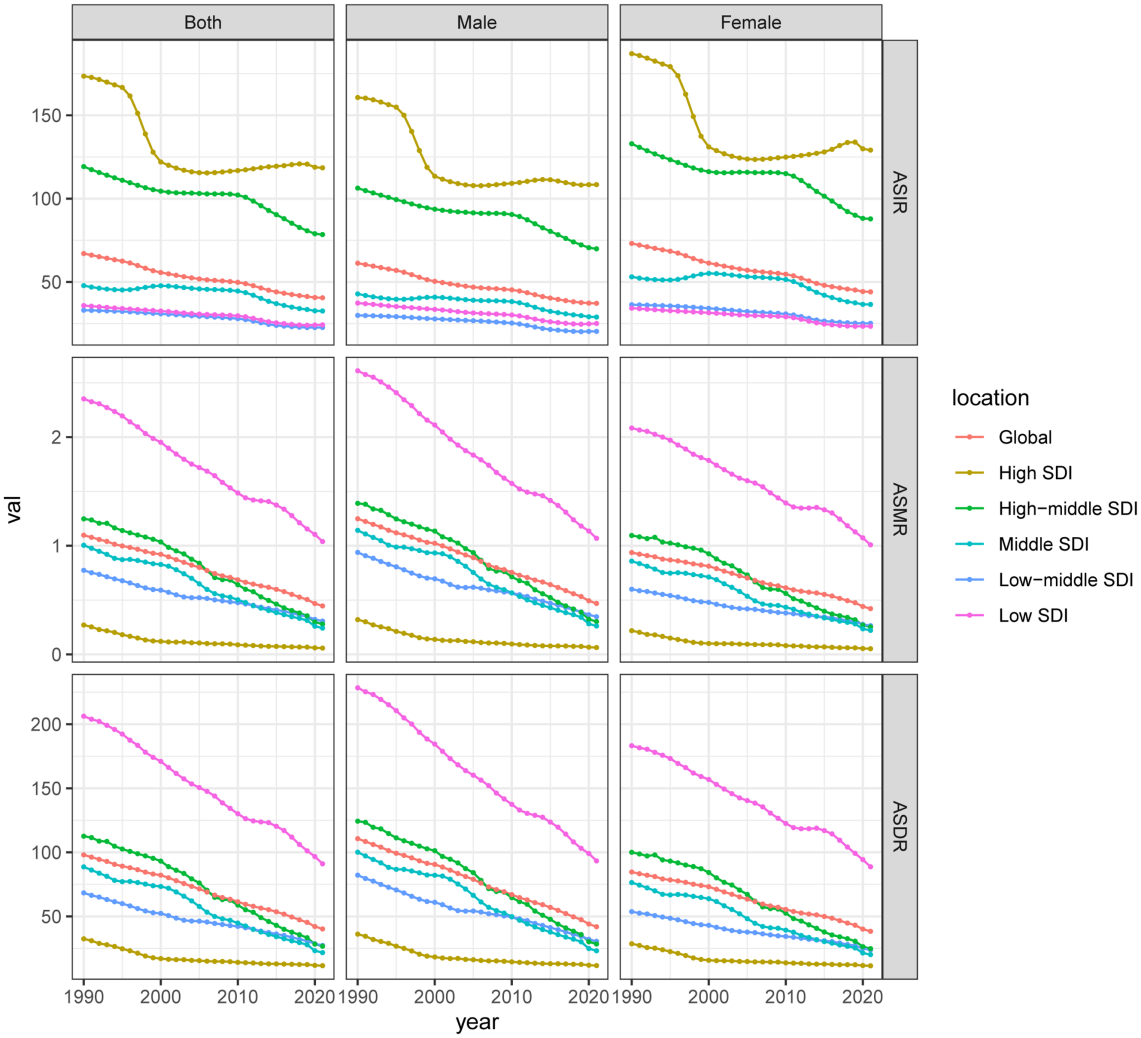
Epidemiological trends in poisoning incidence, mortality, and DALYs rates in children across five SDI regions from 1990 to 2021. ASIR, age-standardized incidence rates; ASMR, age-standardized mortality rate; ASDR, age-standardized disability-adjusted life years rate; DALYs, disability-adjusted life years; SDI, Sociodemographic Index.

The global burden of poisoning among children < 14 years old declined from 1990 to 2021. The number of poisoning cases declined by 29.25%, from 1,163,111.48 (95% UI: 798,334.75–1,664,230.44) in 1990 to 822,877.52 (95% UI: 550,949.58–1,197,683.25) in 2021. The ASIR decreased from 67.04 per 100,000 population (95% UI: 42.03–103.61) in 1990 to 40.46 per 100,000 population (95% UI: 24.87–63.29) in 2021, with an EAPC of −1.63% (95% CI: −1.69 to −1.57). The number of deaths decreased by 54.98%, from 19,283.64 (95% UI: 15,278.57–24,433.41) in 1990 to 8,681.59 (95% UI: 5,638.47–12,296.79) in 2021. The ASMR declined from 1.1 per 100,000 population (95% UI: 0.85–1.41) in 1990 to 0.45 per 100,000 population (95% UI: 0.28–0.65) in 2021, with an EAPC of −2.73% (95% CI: −2.87 to −2.59). DALYs decreased from 1,722,547.46 (95% UI: 1,378,810.36–2,173,390.45) in 1990 to 782,877.25 (95% UI: 528,958.14–1,102,844.78) in 2021 while ASDR declined from 98.04 per 100,000 population (95% UI: 76.59–124.76) to 40.2 per 100,000 population (95% UI: 26.32–57.93), with an EAPC of −2.71% (95% CI: −2.85 to −2.57) (Table 1; Figure 1).

**Table 1:** Age-standardized incidence rate, mortality rate, and disability-adjusted life years rate of childhood poisoning in 1990 and 2021.

Location	ASIR (95%UI)		EAPC (95%CI)	ASMR (95%UI)		EAPC (95%CI)	ASDR (95%UI)		EAPC (95%CI)
	1990	2021		1990	2021		1990	2021	
Global	67.04 (42.03–103.61)	40.49 (24.87–63.29)	−1.63 (−1.69 to −1.57)	1.10 (0.85–1.41)	0.45 (0.28–0.65)	−2.73 (−2.87 to −2.59)	98.04 (76.59–124.76)	40.20 (26.32–57.93)	−2.71 (−2.85 to −2.57)
Sex									
Male	61.27 (38.41–94.46)	37.16 (22.91–57.91)	−1.61 (−1.68 to −1.55)	1.25 (0.92–1.64)	0.47 (0.29–0.71)	−2.95 (−3.11 to −2.79)	110.67 (82.61–144.19)	41.94 (26.71–62.90)	−2.94 (−3.09 to −2.79)
Female	73.13 (45.74–113.31)	44.04 (26.94–69.12)	−1.64 (−1.70 to −1.59)	0.94 (0.57–1.34)	0.42 (0.23–0.61)	−2.44 (−2.56 to −2.31)	84.65 (53.07–119.68)	38.36 (22.13–54.59)	−2.42 (−2.54 to −2.29)
SDI									
High SDI	173.50 (107.89–267.73)	118.49 (72.06–186.2)	−1.26 (−1.62 to −0.91)	0.27 (0.24–0.30)	0.06 (0.05–0.07)	−4.39 (−4.74 to −4.03)	32.49 (28.44–37.63)	11.52 (9.06–14.78)	−3.03 (−3.40 to −2.66)
High–middle SDI	119.24 (77.40–179.05)	78.44 (49.99–118.91)	−1.15 (−1.29 to −1.00)	1.25 (1.06–1.70)	0.28 (0.21–0.34)	−4.61 (−4.98 to −4.24)	112.60 (96.10–151.99)	26.71 (21.11–32.03)	−4.45 (−4.79 to −4.10)
Middle SDI	47.79 (29.15–74.53)	32.55 (19.47–51.69)	−1.13 (−1.39 to −0.88)	1.00 (0.79–1.50)	0.24 (0.14–0.32)	−4.31 (−4.63 to −3.98)	88.65 (69.66–130.79)	21.72 (13.36–28.32)	−4.27 (−4.59 to −3.95)
Low–middle SDI	33.04 (20.20–52.02)	22.68 (13.70–35.52)	−1.35 (−1.47 to −1.22)	0.77 (0.42–1.03)	0.31 (0.18–0.48)	−2.64 (−2.76 to −2.52)	68.36 (38.29–89.85)	27.30 (16.63–42.10)	−2.63 (−2.75 to −2.51)
Low SDI	35.82 (21.94–55.57)	24.24 (15.02–36.92)	−1.35 (−1.45 to −1.24)	2.35 (1.56–3.16)	1.04 (0.64–1.62)	−2.48 (−2.59 to −2.38)	206.25 (137.89–275.94)	91.06 (56.37–141.25)	−2.49 (−2.59 to −2.39)
Region									
Central Asia	150.15 (94.44–226.39)	114.01 (68–177.59)	−0.84 (−0.93 to −0.75)	1.17 (0.99–1.36)	0.38 (0.30–0.50)	−5.06 (−5.79 to −4.33)	107.81 (91.37–124.57)	36.45 (28.91–47.58)	−4.85 (−5.54 to −4.16)
Central Europe	355.03 (236.39–519.37)	233.98 (137.69–372.06)	−1.23 (−1.34 to −1.11)	1.24 (1.12–1.36)	0.10 (0.08–0.11)	−7.93 (−8.19 to −7.67)	121.79 (108.94–135.89)	17.22 (13.41–22.11)	−6.22 (−6.38 to −6.05)
Eastern Europe	193.93 (125.81–288.03)	113.32 (73.38–166.43)	−1.91 (−2.07 to −1.75)	2.03 (1.92–2.13)	0.57 (0.52–0.61)	−4.14 (−4.59 to −3.70)	180.18 (169.98–190.86)	50.77 (45.77–55.15)	−4.15 (−4.58 to −3.72)
Australasia	157.08 (94.61–248.93)	136.96 (82.89–216.09)	−0.42 (−0.48 to −0.37)	0.09 (0.08–0.11)	0.03 (0.02–0.03)	−3.79 (−4.35 to −3.21)	17.23 (13.23–22.32)	10.35 (7.09–14.83)	−1.59 (−1.81 to −1.38)
High–income Asia Pacific	201.41 (126.61–309.16)	135.69 (78.59–218.47)	−1.29 (−1.38 to −1.21)	0.27 (0.14–0.37)	0.02 (0.02–0.03)	−7.97 (−8.40 to −7.53)	33.50 (22.44–42.74)	8.72 (6.01–12.19)	−4.12 (−4.46 to −3.78)
Southern Latin America	378.22 (244.41–568.62)	329.75 (211.67–495.48)	−0.07 (−0.32 to 0.19)	0.69 (0.63–0.76)	0.21 (0.17–0.24)	−2.97 (−3.34 to −2.60)	82.12 (70.87–95.22)	35.99 (27.61–46.14)	−1.99 (−2.29 to −1.69)
Western Europe	173.71 (108.79–271.16)	138.28 (85.67–213.61)	−0.69 (−0.74 to −0.65)	0.15 (0.14–0.16)	0.02 (0.02–0.02)	−6.60 (−7.08 to −6.12)	23.77 (19.56–29.29)	10.31 (7.08–14.39)	−2.59 (−2.86 to −2.31)
Andean Latin America	104.03 (66.14–154.53)	69.34 (40.53–110.72)	−1.27 (−1.32 to −1.21)	1.33 (0.98–1.70)	0.22 (0.16–0.33)	−5.76 (−6.04 to −5.49)	120.29 (89.89–152.11)	21.77 (15.69–30.91)	−5.52 (−5.78 to −5.27)
Caribbean	107.23 (66.21–166.64)	92.39 (56.24–144.90)	−0.32 (−0.44 to −0.20)	1.61 (0.88–2.56)	0.47 (0.26–0.86)	−3.73 (−4.30 to −3.17)	146.14 (82.96–229.45)	45.51 (26.30–80.07)	−3.51 (−4.05 to −2.98)
Central Latin America	178.28 (109.92–274.64)	126.85 (75.65–202.2)	−0.8 (−1.14 to −0.45)	0.87 (0.80–0.95)	0.15 (0.12–0.19)	−5.03 (−5.36 to −4.70)	85.25 (76.99–95.01)	19.51 (15.29–24.97)	−4.17 (−4.41 to −3.93)
Tropical Latin America	47.92 (26.74–79.36)	29.34 (16.95–47.27)	−2.66 (−3.08 to −2.23)	0.20 (0.17–0.23)	0.03 (0.02–0.04)	−5.65 (−5.90 to −5.41)	20.06 (17.45–23.17)	4.01 (3.16 − 5.05)	–5.00 (−5.17 to −4.83)
North Africa and Middle East	37.56 (23.25–57.95)	22.62 (13.08–36.02)	−1.65 (−1.78 to −1.52)	1.72 (0.97–2.45)	0.44 (0.26–0.70)	−4.23 (−4.45 to −4.00)	149.23 (84.99–212.39)	38.55 (22.54–60.08)	−4.24 (−4.46 to −4.02)
South Asia	19.26 (11.41–31.00)	11.79 (6.89–19.01)	−1.68 (−1.86 to −1.49)	0.51 (0.23–0.74)	0.13 (0.08–0.21)	−3.95 (−4.11 to −3.79)	43.98 (20.93–64.32)	11.63 (7.02–18.64)	−3.94 (−4.09 to −3.79)
East Asia	33.52 (20.11–52.44)	25.28 (15.53–39.28)	−0.56 (−0.76 to −0.36)	1.61 (1.19–2.78)	0.42 (0.21–0.59)	−3.93 (−4.58 to −3.28)	139.26 (103.14–239.64)	36.16 (18.41 − 49.85)	–4.00 (−4.65 to −3.34)
Oceania	25.05 (15.35–38.19)	21.90 (13.81–32.78)	−0.47 (−0.62 to −0.31)	0.86 (0.42–1.56)	0.59 (0.29–1.11)	−1.15 (−1.31 to −0.98)	72.00 (35.92–129.94)	49.42 (24.95–92.39)	−1.14 (−1.31 to −0.97)
Southeast Asia	28.51 (16.81–45.81)	21.03 (12.36–34.41)	−0.86 (−1.06 to −0.67)	0.17 (0.09–0.27)	0.08 (0.05–0.11)	−2.08 (−2.24 to −1.92)	15.69 (9.06–24.67)	7.40 (4.94–9.95)	−2.02 (−2.15 to −1.88)
Central Sub–Saharan Africa	31.01 (18.74–47.60)	19.28 (11.73–29.71)	−1.60 (−1.79 to −1.40)	2.48 (1.49–3.56)	0.86 (0.42–2.08)	−3.10 (−3.40 to −2.80)	218.35 (131.97–312.59)	75.51 (37.04–181.17)	−3.12 (−3.42 to −2.82)
Eastern Sub–Saharan Africa	44.53 (27.39–68.81)	26.99 (16.69–41.57)	−1.78 (−1.90 to −1.65)	3.04 (2.03–3.96)	1.22 (0.69–2.13)	−2.69 (−2.80 to −2.57)	268.38 (180.08–348.57)	107.24 (61.02–187.11)	−2.70 (−2.81 to −2.58)
Southern Sub–Saharan Africa	36.38 (22.75–56.15)	24.92 (15.52–37.48)	−1.29 (−1.39 to −1.18)	1.62 (1.03–2.14)	1.22 (0.76–1.73)	−0.59 (−0.91 to −0.27)	140.57 (90.05–184.46)	105.37 (65.73–148.84)	−0.60 (−0.93 to −0.28)
Western Sub–Saharan Africa	38.80 (23.55–60.72)	27.67 (17.05–42.08)	−1.17 (−1.31 to −1.02)	1.79 (1.14–2.92)	1.04 (0.64–1.45)	−1.67 (−1.89 to −1.46)	157.71 (100.73–255.99)	90.96 (57.03–126.85)	−1.68 (−1.90 to −1.47)

### 3.2 Global differences by sex and age

In 2021, the ASIR for girls was 44.04 per 100,000 population (95% CI: 26.94–69.12), which was higher than that for boys (37.16 per 100,000 population, 95% CI: 22.91–57.91). However, boys exhibited a higher ASMR (0.47 per 100,000 population, 95% UI: 0.29–0.71) and ASDR (41.94 per 100,000 population, 95% UI: 26.71–62.9) than those of girls (ASMR: 0.42 per 100,000 population, 95% UI: 0.23–0.61; ASDR: 38.36 per 100,000 population, 95% UI: 22.13–54.59). From 1990 to 2021, ASIR, ASMR, and ASDR declined for both sexes (Table 1; Figure 1).

Among the different age groups in 2021, children aged 10–14 years exhibited the highest ASIR (49.06 per 100,000 population, 95% UI: 28.79–80.80), followed by those aged 5–9 years (43.42 per 100,000 population, 95% UI: 25.13–68.68). The lowest ASIR was observed in children < 5 years old (30.01 per 100,000 population, 95% UI: 21.11–42.42). However, children < 5 years exhibited the highest ASMR (0.87 per 100,000 population, 95% UI: 0.53–1.33) and ASDR (77.25 per 100,000 population, 95% UI: 47.78–118.62) than those of the other age groups (Supplementary Table 1; Supplementary Figure 1). Between 1990 and 2021, ASIR, ASMR, and DALYs declined across all age groups (Supplementary Table 1; Supplementary Figure 2).

### 3.3 Sociodemographic index regional trends

In 2021, the number of poisoning cases among children in high-SDI regions was 203,036.49 (95% UI: 136,193.59–299,811.79), with 98.17 deaths (95% UI: 88.97–109.46), and 20,040.11 DALYs (95% UI: 15,872.84–25,555.79). The ASIR was 118.49 per 100,000 population (95% UI: 72.06–186.2), the ASMR was 0.06 per 100,000 (95% UI: 0.05–0.07), and the ASDR was 11.52 per 100,000 (95% UI: 9.06–14.78). In contrast, low-SDI regions reported 110,972.50 incident cases (95% UI: 75,283.76–159,296.01), 4,866.41 deaths (95% UI: 3,091.78–7,458.22), and 426,603.87 DALYs (95% UI: 272,507.15–651,368.22), with an ASIR of 24.24 (95% UI: 15.02–36.92), ASMR of 1.04 per 100,000 (95% UI: 0.64–1.62), and ASDR of 91.06 per 100,000 (95% UI: 56.37–141.25). Between 1990 and 2021, the burden of childhood poisoning decreased across all SDI groups, although the magnitude of the decline varied. The greatest reduction in ASIR was observed in middle-low SDI countries (EAPC: –1.35, 95% CI: −1.47 to −1.22), while high-SDI regions showed the most pronounced declines in ASMR (EAPC: –4.61, 95% CI: −4.98 to −4.24) and ASDR (EAPC: –4.45, 95% CI: −4.79 to −4.10) (Table 1; Figure 1; Supplementary Table 3).

### 3.4 Regional and national trends

**Figure 2:**
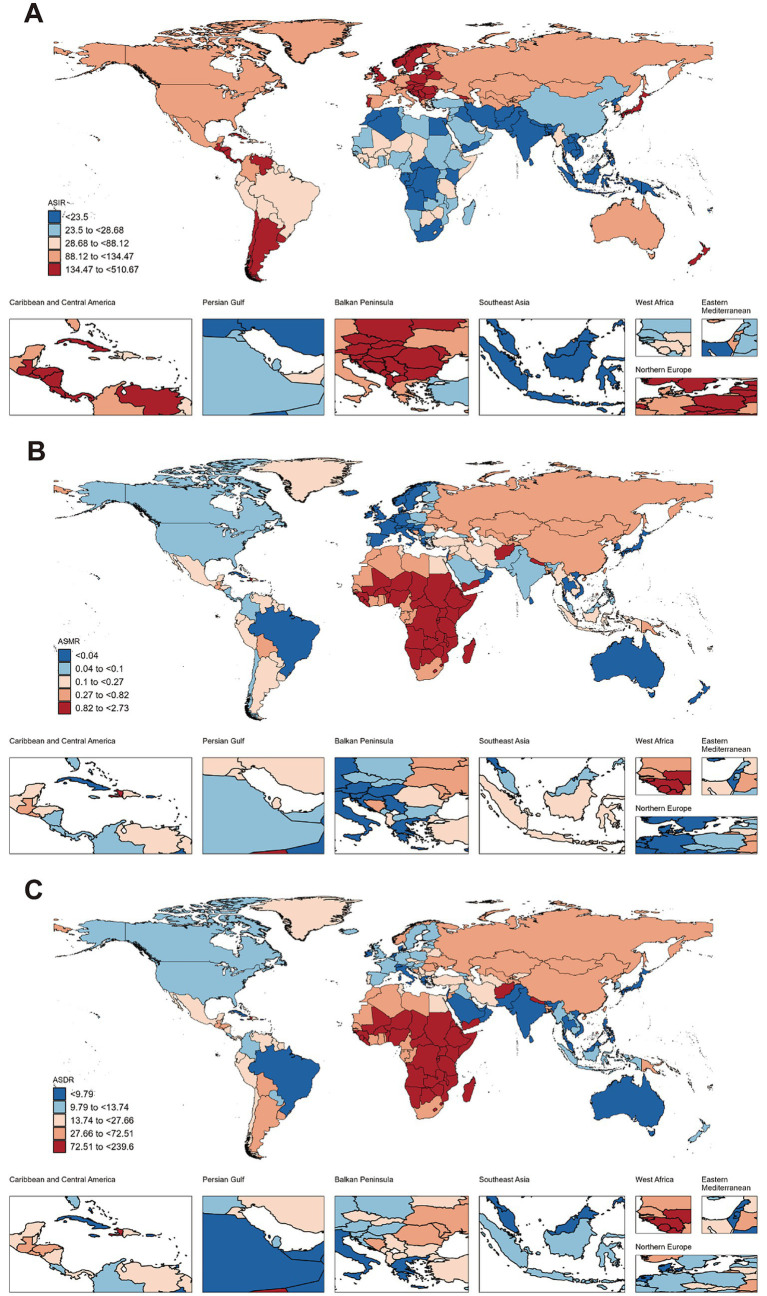
Global burden of childhood poisoning (ages 0–14) across countries in 2021. **(A)** ASIR of childhood poisoning in 2021. **(B)** ASMR of childhood poisoning in 2021. **(C)** ASDR of childhood poisoning in 2021. ASIR, age-standardized incidence rate; ASMR, age-standardized mortality rate; ASDR, age-standardized disability-adjusted life years rate.

Among the 21 GBD regions, Southern Latin America exhibited the highest ASIR for childhood poisoning in 2021, at 329.75 per 100,000 population (95% UI: 211.67–495.48), while South Asia exhibited the lowest, at 11.79 per 100,000 population (95% UI: 6.89–19.01). Between 1990 and 2021, ASIR declined across all regions, with the steepest decline observed in Tropical Latin America (EAPC: -2.66, 95% CI: −3.08 to −2.23) and the smallest in Southern Latin America (EAPC: -0.07, 95% CI: −0.32 to 0.19) (Table 1). Among the 204 countries in 2021, Norway reported the highest ASIR (510.67, 95% UI: 357.14–707.34), followed by Argentina (340.07, 95% UI: 216.08–516.46), Czechia (317.93, 95% UI: 177.68–512.84), Chile (313.17, 95% UI: 200.69–458.61), and Slovenia (298.91, 95% UI: 169.83–481.60). Conversely, the lowest ASIR was reported in India (10.85, 95% UI: 6.15–17.91), followed by Bangladesh (12.23, 95% UI: 7.10–19.43), Bhutan (13.17, 95% UI: 7.40–21.8), Pakistan (14.75, 95% UI: 9.05–22.60), and Cambodia (15.89, 95% UI: 8.73–25.86) (Figure 2A; Supplementary Table 4). Over the same period, Iran experienced the greatest ASIR decline (EAPC: -3.49, 95% CI: −3.82 to −3.15), while Cuba experienced the most significant increase (EAPC: 1.51, 95% CI: 1.18–1.84) (Supplementary Figure 2A; Supplementary Table 4).

In 2021, Southern Sub-Saharan Africa exhibited the highest ASMR for childhood poisoning (1.22 per 100,000 population, 95% UI: 0.76–1.73), while Western Europe exhibited the lowest (0.02 per 100,000 population, 95% UI: 0.02–0.02). The most significant reduction in ASMR was observed in the High-income Asia Pacific region (EAPC: -7.97, 95% CI: −8.4 to −7.53), whereas Southern Sub-Saharan Africa exhibited the smallest decline (EAPC: -0.59, 95% CI: −0.91 to −0.27) (Table 1). South Sudan reported the highest ASMR (2.73, 95% UI: 1.41–4.73), followed by Zimbabwe (2.29, 95% UI: 1.09–3.95), Somalia (1.99, 95% UI: 0.88–4.30), Nepal (1.96, 95% UI: 1.05–3.54), and Burkina Faso (1.92, 95% UI: 0.91–4.65). Conversely, the lowest ASMR was recorded in Andorra (0.00, 95% UI: 0.00–0.00), Singapore (0.00, 95% UI: 0.00–0.00), Switzerland (0.00, 95% UI: 0.00–0.00), Denmark (0.01, 95% UI: 0.00–0.01), and the Netherlands (0.01, 95% UI: 0.01–0.01) (Figure 2B; Supplementary Table 5). During this period, Puerto Rico experienced the most significant ASMR decline (EAPC: -12.79, 95% CI: −13.92 to −11.63), while Cabo Verde recorded the highest increase (EAPC: 3.23, 95% CI: 0.80–5.72) (Supplementary Figure 2B; Supplementary Table 5).

In 2021, Eastern Sub-Saharan Africa exhibited the highest ASDR (107.24 per 100,000 population, 95% UI: 61.02–187.11), while Tropical Latin America exhibited the lowest (4.01 per 100,000 population, 95% UI: 3.16–5.05). Central Europe exhibited the most significant ASDR decline (EAPC: -6.22, 95% CI: −6.38 to −6.05), while Southern Sub-Saharan Africa exhibited the smallest decline (EAPC: -0.60, 95% CI: −0.93 to −0.28) (Table 1). The highest ASDR values were reported in South Sudan (239.60, 95% UI: 125.16–413.09), followed by Zimbabwe (197.91, 95% UI: 94.24–341.39), Somalia (174.38, 95% UI: 78.29–374.37), Burkina Faso (169.63, 95% UI: 81.43–408.54), and the Central African Republic (167.10, 95% UI: 88.52–375.61). Conversely, the lowest ASDR was reported in Oman (2.67, 95% UI: 1.76–3.92), Sri Lanka (2.94, 95% UI: 1.96–4.43), Vietnam (3.21, 95% UI: 1.67–7.32), Maldives (3.31, 95% UI: 1.86–5.93), and Brazil (3.68, 95% UI: 2.90–4.64) (Figure 2C; Supplementary Table 6). During this period, Belize experienced the most significant ASDR decline (EAPC: -9.22, 95% CI: −10.17 to −8.26), while Cabo Verde recorded the most significant increase (EAPC: 1.88, 95% CI: −0.01 to 3.80) (Supplementary Figure 2C; Supplementary Table 6).

### 3.5 Health inequality analysis

**Figure 3:**
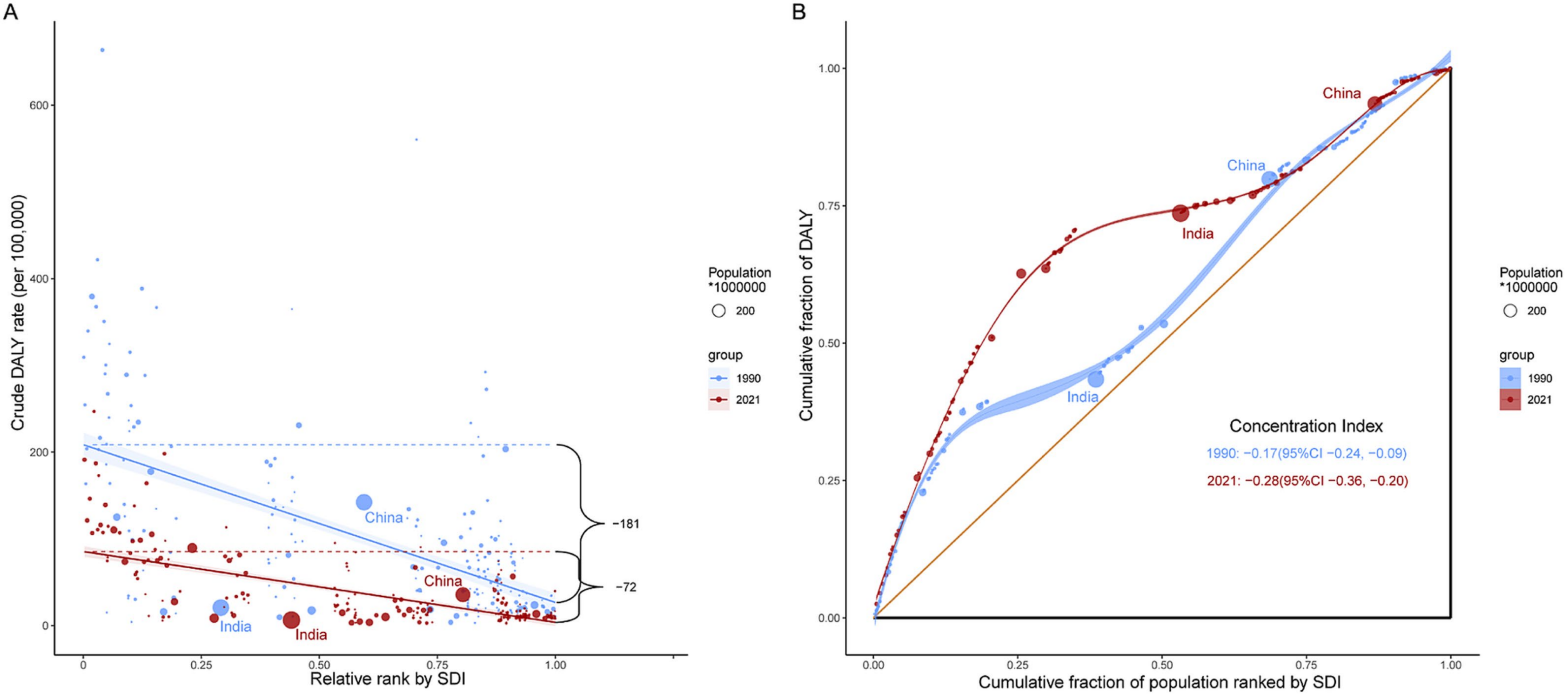
SII and CI for DALYs due to childhood poisoning in 1990 and 2021. **(A)** SII for DALYs due to childhood poisoning in 1990 and 2021. **(B)** CI for DALYs due to childhood poisoning in 1990 and 2021. SII, Slope Index of Inequality; CI, Concentration Index; DALYs, disability-adjusted life years.

In 1990, the SII for ASDR in children < 14 years old was −181.38 (95% CI: −206.19 to −156.57) and decreased to −71.80 (95% CI: −82.65 to −60.96) in 2021 (Figure 3A). Similarly, the CI for ASDR shifted from −0.17 (95% CI: −0.24 to −0.09) in 1990 to −0.28 (95% CI: −0.36 to −0.20) in 2021 (Figure 3B). These findings indicate that while the absolute gap in ASDR between high- and low-SDI countries narrowed, lower-SDI countries continue to experience a disproportionately higher burden.

Similarly, the SII for ASIR declined from 140.54 (95% CI: 113.47–167.61) in 1990 to 114.64 (95% CI: 94.94–134.33) in 2021. The CI for ASIR declined from 0.42 (95% CI: 0.36–0.48) in 1990 to 0.37 (95% CI: 0.29–0.45) in 2021. These findings suggest that while the incidence gap between high- and low-SDI countries narrowed, high-SDI countries continue to account for a greater share of the poisoning incidence burden (Supplementary Figure 3).

### 3.6 Frontier analysis

**Figure 4:**
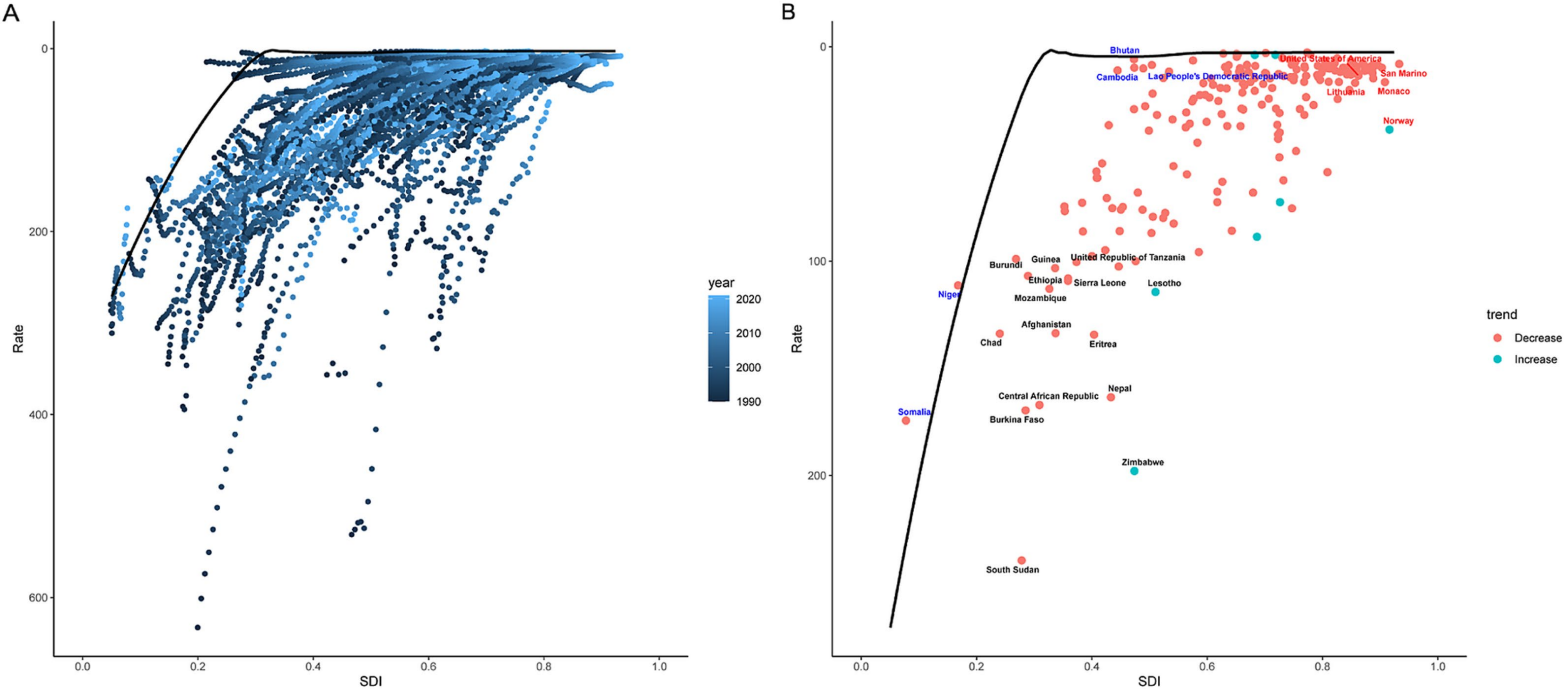
Frontier analysis of DALYs for childhood poisoning in 2021. A solid black line represents the frontier, while dots indicate countries and territories. Black indicates the top 15 countries with the highest EF in childhood poisoning burden (the largest gap in ASDR from the frontier). Blue represents examples of frontier countries with low SDI (< 0.5) and reduced EF, while red indicates those with high SDI (> 0.85) and relatively elevated EF for their development level. Red dots indicate a reduction in the burden of childhood poisoning between 1990 and 2021, whereas blue dots indicate an increase in burden over the same period. ASDR, age-standardized disability-adjusted life years rate; EF, effective differences; and SDI, sociodemographic index.

In the analysis of ASDR, the EF decreased with increasing SDI. In 2021, the 10 countries with the highest EF in ASDR were South Sudan, Zimbabwe, Burkina Faso, Central African Republic, Nepal, Eritrea, Afghanistan, Lesotho, Mozambique, and Sierra Leone, with values ranging from 230.36 to 104.50. In contrast, the 10 countries with the lowest EF were Niger, Somalia, Oman, Sri Lanka, Vietnam, Maldives, Brazil, Thailand, Mauritius, and the Cook Islands, with values ranging from 0.00 to 1.09 (Figure 4; Supplementary Table 7).

For ASIR, the highest EF values were observed in Norway, Argentina, Czechia, Chile, Slovenia, Bosnia and Herzegovina, Serbia, Hungary, Uruguay, and Slovakia, ranging from 245.92 to 499.79. The lowest EF values were recorded in India, Bangladesh, Somalia, Niger, Bhutan, Pakistan, Timor-Leste, Afghanistan, Cambodia, and the Lao People’s Democratic Republic, ranging from 0.00 to 0.01 (Supplementary Figure 4; Supplementary Table 8).

## 4 Discussion

Data from the GBD database was used to investigate the burden of poisoning among children < 14 years old at global, regional, and national levels from 1990 to 2021. Temporal trends, health inequalities, and potential areas for intervention were analyzed, providing new insights into the epidemiology of childhood poisoning.

Several key findings were observed. First, the global burden of childhood poisoning declined significantly from 1990 to 2021, but the trends varied across regions. Second, older children exhibited higher incidence rates, whereas younger children experienced higher mortality and DALY rates. Third, high-SDI countries reported higher incidence rates than other countries, but mortality and DALYs were lower. Conversely, low-SDI countries showed the opposite pattern. Fourth, although the absolute gap in the burden of poisoning-related DALYs between countries with different SDI levels decreased from 1990 to 2021, children in low-SDI countries still face a disproportionately higher burden. Fifth, frontier analysis revealed a negative correlation between ASDR, and SDI. However, significant heterogeneity was observed among countries with similar SDI levels.

Unlike poisoning in adults, childhood poisoning primarily stems from environmental exposure rather than occupational exposure. Targeted interventions addressing various risk factors can significantly reduce its burden. This study showed a substantial global decline in ASIR, ASMR, and ASDR for childhood poisoning from 1990 to 2021. This progress indicates collaborative efforts from multiple stakeholders worldwide, including designers improving child-resistant medication packaging (12, 13), public health officials implementing policies (14), community educators (15), and clinical emergency responders (16). However, this trend is not uniform across all regions. Over the past 15 years, high-SDI regions have experienced fluctuations in childhood poisoning incidence. Retrospective clinical studies show that trends vary by country. For example, childhood poisoning rates have declined in the United States (17), remained stable in South Korea (18), and increased in Turkey (19). These fluctuations may result from factors such as advancements in statistical methods, the influence of social media, and changes in pharmaceutical and chemical accessibility. In high-SDI countries, improved surveillance and reporting systems allow families to seek medical assistance not only from traditional healthcare institutions but also through poison control hotlines (20). These expanded reporting mechanisms may contribute to increased documentation of poisoning cases that previously went unrecorded, potentially inflating incidence rates in certain years. Additionally, social media has influenced childhood poisoning trends. Reports show that some children ingest excessive amounts of diphenhydramine to induce hallucinations as part of online challenges, temporarily increasing poisoning cases (21). Furthermore, the predominant types of childhood poisoning have evolved over time (1, 22). Retrospective studies from countries such as the Czech Republic and Brazil show increasing rates of medication-related poisonings among children (23, 24). This may be due to the widespread availability of medications in households and limited parental awareness regarding safe storage (25). In contrast, poisoning from household fuels and pesticides has declined, indicating strengthened public health interventions (26). These changes highlight that, despite an overall decline in the burden of childhood poisoning, continued vigilance is necessary, as poisoning remains a significant threat to the health of children.

This study showed that poisoning incidence was higher in children aged 10–14 years than in other age groups, differing from most studies (20, 27, 28). This discrepancy may stem from differences in data sources, as previous studies often relied on data from single treatment centers or poison control centers. Older children with mild symptoms may remain at home under parental observation rather than seek medical care, leading to underreporting of cases in previous studies. Additionally, differences in children’s behavioral awareness and supervision may further contribute to this trend. As children grow older, their ability to engage in independent activities increases, while parental supervision tends to decrease, significantly raising their chances of exposure to medications and household chemicals. However, due to a lack of full awareness of the potential dangers of these substances, they may misuse household chemicals or take excessive doses of medications, contributing to a higher incidence of poisoning in this age group. In contrast, although children aged 0–4 years have a lower incidence of poisoning, they exhibit significantly higher mortality and DALY rates than older children. This is largely attributed to physiological factors; younger children have immature metabolic and liver enzyme systems, reducing their ability to detoxify harmful substances. Consequently, the same doses of a toxic agent have a greater effect on them. Moreover, their limited verbal communication makes poisoning cases harder to detect, often only becoming evident when symptoms appear or caregivers recognize potential exposure. This delay in seeking medical care may worsen health outcomes (1). These age-specific epidemiological patterns highlight the need for targeted poisoning prevention strategies at different developmental stages. Community health workers should tailor educational interventions for parents and caregivers. For young children, parents and guardians should be encouraged to store hazardous substances in secure containers or in places that are out of the reach of children. Retrospective studies show that modifying medication packaging effectively prevents childhood poisoning (29). For older children, it is essential to enhance their awareness and education regarding hazardous substances while simultaneously strengthening supervision and parental monitoring.

This study showed that children in low-SDI countries have a lower incidence of poisoning but significantly higher mortality and DALY rates than those in high-SDI countries, which shows the opposite pattern. This finding contrasts with previous assumptions that lower-income countries would experience both higher incidence and mortality rates. Several factors may contribute to these differences, including toxic exposure types, social behavior patterns, and healthcare capacity. In high-SDI countries, greater availability of industrialized products, such as cosmetics, over-the-counter medications, and prescription drugs, potentially contributes to higher poisoning incidence. Moreover, gaps in the enforcement of child-resistant packaging regulations may leave children more vulnerable. The predominance of nuclear families also means that children may spend more time unsupervised, increasing the chances of accidental ingestion or misuse of toxic substances. Additionally, more robust surveillance and reporting systems in high-SDI settings may capture a greater number of mild poisoning cases, contributing to the higher observed incidence. Nonetheless, well-established healthcare systems in these regions enable timely identification, treatment, and referral of poisoning cases, leading to markedly improved outcomes. Greater accessibility to primary healthcare and poison control centers, along with better baseline nutritional status and physiological resilience among children, likely reduces mortality and long-term disability associated with poisoning. Conversely, low-SDI countries generally have less exposure to industrialized products, and multi-generational households are more prevalent, reducing unsupervised time for children and lowering poisoning incidence. However, weaker regulatory oversight of highly toxic substances, such as pesticides and industrial chemicals, allows greater accessibility to highly lethal poisons. Furthermore, the limited availability of emergency medical resources in low-SDI countries further exacerbates poisoning outcomes. Even in treatable cases, delays in treatment and inadequate healthcare infrastructure contribute to high mortality and disability burdens.

The health inequality analysis in this study revealed that while the absolute disparity between countries at different development levels has decreased from 1990 to 2021, children in low-SDI countries still experience a significantly higher disability burden. Furthermore, frontier analysis revealed that some countries have considerable potential for reducing childhood poisoning burdens. For poisoning-related diseases, both prevention and treatment are equally important; however, the burden varies across countries. In some high-SDI countries, the relatively high EF in ASIR suggests an urgent need to strengthen preventive strategies for childhood poisoning. Conversely, in certain low-SDI countries, the relatively high EF in ASDR indicates that, while prevention remains important, immediate improvements in treatment capacity and emergency response systems are critically needed. Mary et al. reported that integrating preventive educational interventions with regulatory measures to improve drug storage and formulation safety can help reduce the burden of pediatric drug poisoning (30). Flemming et al. reported that securing pesticides in safe storage environments is an effective strategy for preventing severe poisoning cases (31). Yura et al. demonstrated that round, colorful, and small-sized capsules and tablets are visually appealing to children, increasing the risk of accidental ingestion and poisoning (32). Additionally, Noah et al. reported that the identification of unknown products by children is influenced by their physical characteristics, and unclear packaging of household chemicals may increase the risk of accidental ingestion (13). In high-SDI countries, reinforcing caregiver education on the safe storage of household chemicals, enhancing the regulation of packaging and physical characteristics of household products and medications, and optimizing home safety settings may serve as effective strategies to prevent childhood poisoning and reduce its incidence. Advanced healthcare systems in high-SDI countries ensure access to extensive poisoning treatment resources, including dialysis, circulatory support, and antidotal therapy, which significantly enhance survival outcomes. However, low-SDI countries often face resource constraints that delay access to specialized medical treatment, exacerbating the health burden. Therefore, enhancing poisoning treatment capacity in these countries— particularly by strengthening emergency response systems and optimizing primary healthcare facilities to effectively manage poisoning cases—is essential for reducing childhood poisoning-related disabilities. Although there is no universal antidote, the management of poisoning generally follows a series of standardized protocols. Many of these steps offer cost-effective options that can be adapted according to the economic capacities of low-income countries and regions. Timely treatment after toxic exposure is critical. Anar et al. reported that pediatric poisoning cases treated in intensive care units experience significantly longer delays between exposure and hospital admission than those managed in general wards (19). Since most poisoning incidents occur at home, providing parents with first-aid training for poisoning emergencies could reduce the time between exposure and medical intervention, improving patient outcomes (33, 34). For most types of poisoning, prompt elimination of toxins from the body or prevention of further absorption remains an effective treatment strategy. However, in many primary healthcare facilities, the lack of essential equipment such as gastric lavage devices and insufficient training of healthcare personnel pose significant challenges to early management. Strengthening the training of frontline medical staff is crucial to ensure timely recognition and treatment of pediatric poisoning cases. In addition, while standard adsorbents like activated charcoal are often unavailable in these settings, low-cost alternatives—such as montmorillonite powder (a gastrointestinal adsorbent), mannitol (a diuretic agent), or even milk (used to dilute corrosive substances in the digestive tract)—can be considered feasible substitutes and should be stocked in primary healthcare institutions. In agricultural regions with high rates of pesticide poisoning, medical facilities should ensure the availability of specific antidotes, such as pralidoxime, for organophosphate poisoning. Additionally, for pediatric patients with unidentified toxic exposures, initial diagnosis based on clinical symptoms and prompt, empirical treatment should be administered without waiting for laboratory confirmation to minimize delays and improve survival rates (16). Following initial detoxification, maintaining organ function and stabilizing vital signs are also critical components of treatment. Enhancing the capacity of primary healthcare facilities to recognize severe pediatric poisoning cases, along with strengthening referral systems and coordination with higher-level hospitals, is essential. Timely transfer of patients who have received initial management to advanced medical centers ensures access to intensive care and improves overall outcomes.

This study has some limitations. First, this study is a secondary analysis based on data from the GBD database, which uses multiple sources to construct statistical models. In regions where data are missing, estimates are generated based on data from neighboring areas or from countries with similar socioeconomic and demographic characteristics. While this approach improves comparability across countries, it may introduce uncertainty in estimating the burden of poisoning in data-scarce regions. In contrast, high-income regions often have more comprehensive surveillance systems and better case documentation through poison control centers and healthcare facilities, which may lead to more accurate data capture compared to low-income regions. As a result, information bias may exist. In the future, the development of standardized and harmonized methods for poisoning surveillance could enhance the reliability and comparability of global poisoning burden assessments. Second, due to constraints in the GBD 2021 database, this study did not analyze the burden of specific poisoning types (e.g., medications, household chemicals, or pesticides) in children. Future research targeting specific poisoning types would provide valuable insights for prevention strategies and policy development. Third, it is important to note that our study, based on GBD definitions, only includes unintentional poisoning cases. Research has shown that as children grow older, the number of intentional poisoning cases also increases (17, 20). Future studies should explore the distinctions between intentional and unintentional poisoning in children to gain a more comprehensive understanding of poisoning trends among adolescents. Fourth, significant disparities in economic status and healthcare access often exist within countries (e.g., urban vs. rural areas). Therefore, findings from the health inequality and frontier analyses may not fully capture these variations. Policymaking and resource allocation should, therefore, be tailored to the specific local contexts of each region.

## 5 Conclusion

From 1990 to 2021, the global burden of poisoning among children under 14 years old has declined. However, childhood poisoning remains a significant public health concern. Although the disparities between high- and low-development countries have narrowed, lower-development countries continue to experience a higher burden of disease and disability. Therefore, different region-specific policies and interventions are essential based on their development status. In low-development countries, efforts should focus on strengthening emergency response systems for poisoning incidents. In high-development countries, the focus should shift to advancing poisoning prevention strategies and enforcing safe storage regulations. Additionally, further research is needed to explore the risk factors associated with childhood poisoning.

## Data Availability

Publicly available datasets were analyzed in this study. This data can be found here: https://vizhub.healthdata.org/gbd-results/.
