# Supplementary material for: Global, regional, and national burden of unintentional childhood poisoning, 1990–2021: an analysis of data from the Global Burden of Disease study 2021
Source: Front Public Health. 2025 Aug 5;13:1596599. doi: 10.3389/fpubh.2025.1596599 (PMC12361209; doi:10.3389/fpubh.2025.1596599)
Supplement: Supplementary file 1 [file Data_Sheet_1.docx]

Supplementary Material


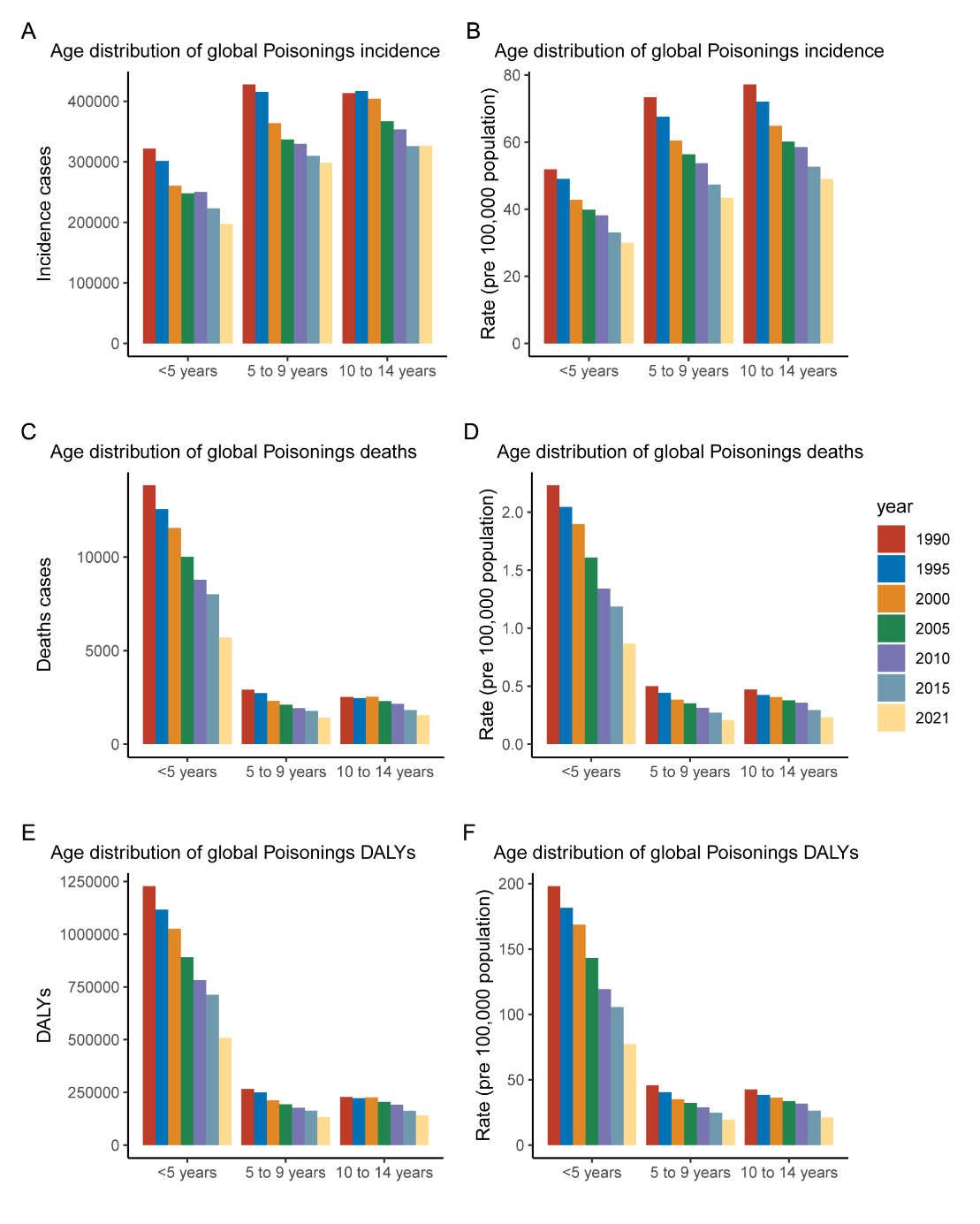


**Supplementary Figure 1.** Trends in poisoning burden in children under 14 years old from 1990 to 2021 **(A)** trends in Incidence cases **(B)** trends in Incidence rate **(C)** trends in Deaths cases **(D)** trends in Deaths rate **(E)** trends in DALYs **(F)** trends in DALYs rate

**
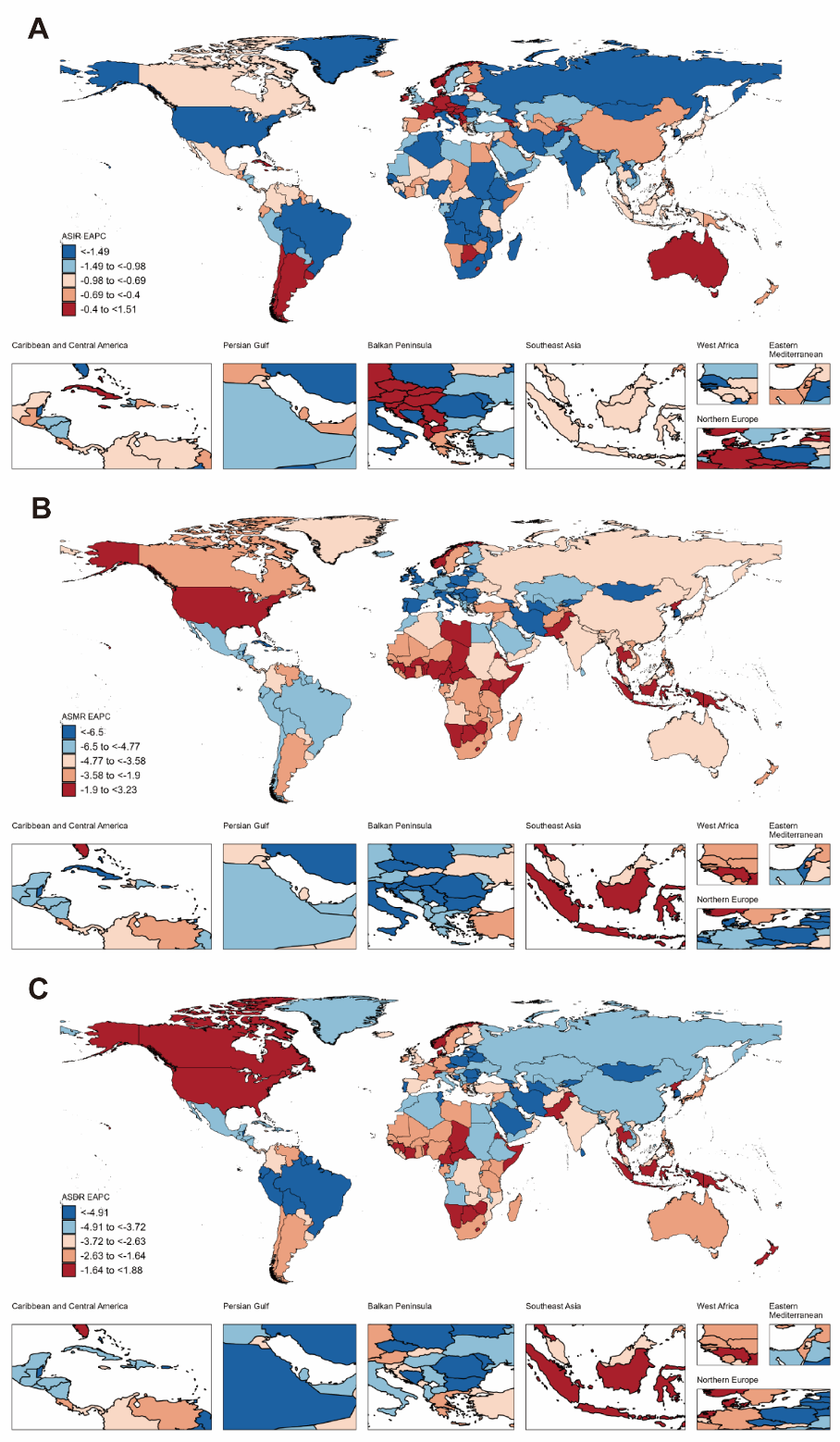
**

**Supplementary Figure 2.** Global burden of childhood poisoning (ages 0–14) across countries from 1990 to 2021. (A) EAPC in ASIR from 1990 to 2021. (B) EAPC in ASMR from 1990 to 2021. (C) EAPC in ASDR from 1990 to 2021. ASIR, age-standardized incidence rate; ASMR, age-standardized mortality rate; ASDR, age-standardized disability-adjusted life years rate; EAPC, Estimated Annual Percentage Change.


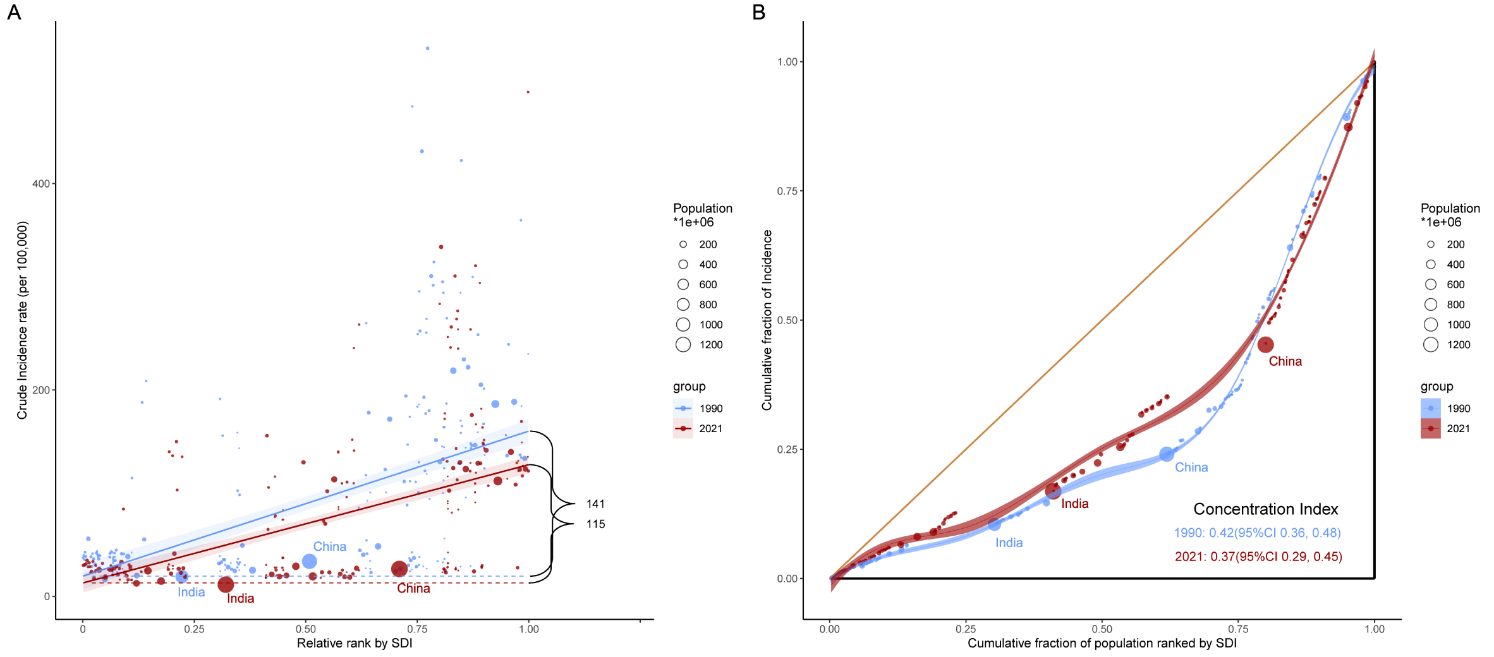


**Supplementary Figure 3.** SII and CI for incidence due to childhood poisoning in 1990 and 2021. (A) SII for incidence due to childhood poisoning in 1990 and 2021. (B) CI for incidence due to childhood poisoning in 1990 and 2021. SII, Slope Index of Inequality; CI, Concentration Index.


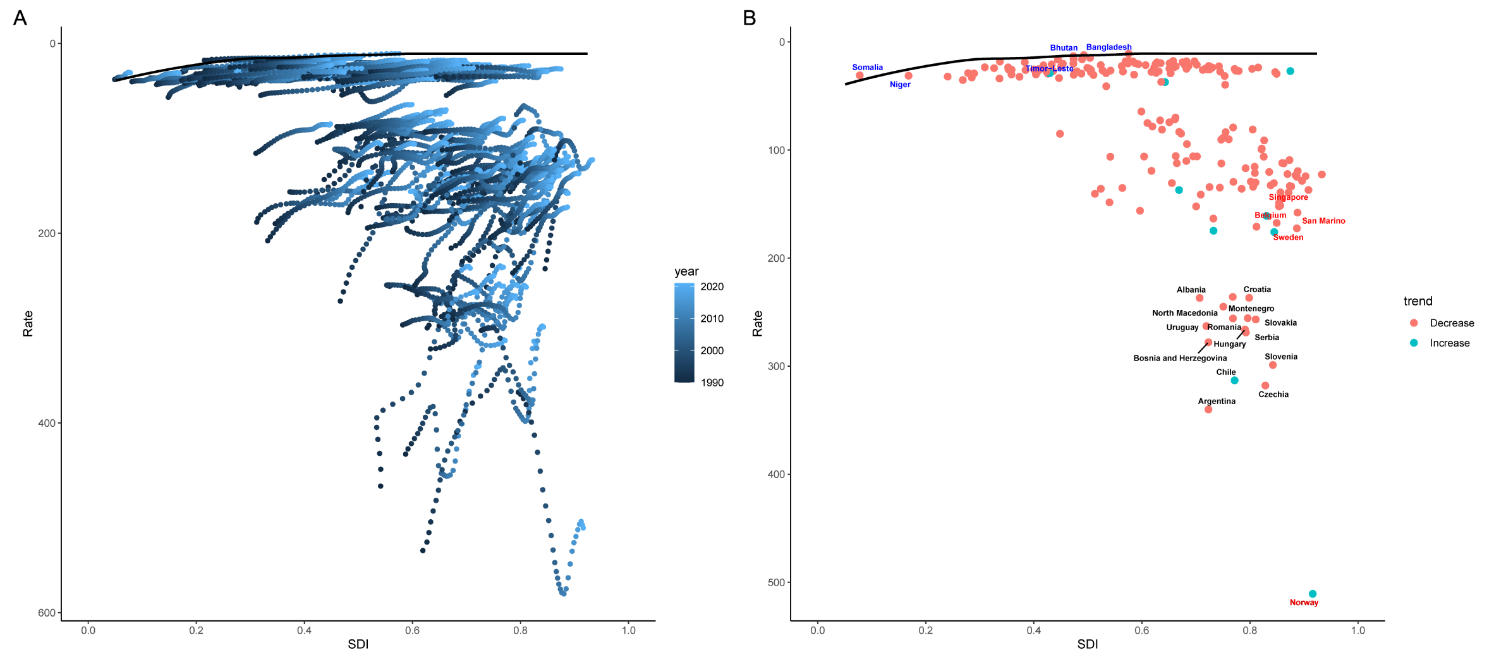


**Supplementary Figure 4.** Frontier analysis of incidence for childhood poisoning in 2021. A solid black line represents the frontier, while dots indicate countries and territories. Black indicates the top 15 countries with the highest EF in childhood poisoning burden (the largest gap in ASIR from the frontier). Blue represents examples of frontier countries with low SDI (< 0.5) and reduced EF, while red indicates those with high SDI (> 0.85) and relatively elevated EF for their development level. Red dots indicate a reduction in the burden of childhood poisoning between 1990 and 2021, whereas blue dots indicate an increase in burden over the same period. ASIR, age–standardized incidence rate; EF, effective differences; and SDI, sociodemographic index.

**Supplementary Table 1.** ICD Classification and Code Descriptions of Poisoning According to the GBD Definition

| ICD Code | Code Description |
| --- | --- |
| **ICD9** |  |
| E856 | Accidental poisoning by antibiotics |
| E857 | Accidental poisoning by other anti-infectives |
| E860 | Accidental poisoning by alcohol, not elsewhere classified |
| E861 | Accidental poisoning by cleansing and polishing agents, disinfectants, paints, and varnishes |
| E862 | Accidental poisoning by petroleum products, other solvents and their vapors, not elsewhere classified |
| E863 | Accidental poisoning by agricultural and horticultural chemical and pharmaceutical preparations other than plant foods and fertilizers |
| E864 | Accidental poisoning by corrosives and caustics, not elsewhere classified |
| E865 | Accidental poisoning from poisonous foodstuffs and poisonous plants |
| E867 | Accidental poisoning by gas distributed by pipeline |
| E868 | Accidental poisoning by other utility gas and other carbon monoxide |
| E869 | Accidental poisoning by other gases and vapors |
| E929.2 | Late effects of accidental poisoning |
| **ICD10** |  |
| J70.5 | Respiratory conditions due to smoke inhalation |
| X46 | Accidental poisoning by and exposure to organic solvents and halogenated hydrocarbons and their vapours |
| X47 | Accidental poisoning by and exposure to carbon monoxide and other gases and vapours |
| X48 | Accidental poisoning by and exposure to pesticides |

**Supplementary Table 2.** Trends in Incidence, Mortality, and Disability–Adjusted Life Years (DALYs) Rate of Childhood Poisoning by Age Group, 1990–2021

| age group | Incidence rate  (95% UI) | | EAPC  (95%CI) | Mortality rate  (95% UI) | | EAPC  (95%CI) | DALYs rate  (95% UI) | | EAPC  (95%CI) |
| --- | --- | --- | --- | --- | --- | --- | --- | --- | --- |
|  | 1990 | 2021 |  | 1990 | 2021 |  | 1990 | 2021 |  |
| <5 years old | 51.91(37.50 – 72.67) | 30.01(21.11 – 42.42) | –1.81 (–1.89 to –1.74) | 2.23(1.67 – 2.94) | 0.87(0.53 – 1.33) | –2.91 (–3.07 to –2.75) | 198.17(149.16 – 260.21) | 77.25(47.79 – 118.62) | –2.9 (–3.06 to –2.74) |
| 5–9 years old | 73.32( 43.56 – 114.84) | 43.42(25.13 – 68.68) | –1.67 (–1.72 to –1.62) | 0.50(0.41 – 0.60) | 0.21(0.14 – 0.27) | –2.56 (–2.68 to –2.44) | 45.56(38.22 – 52.89) | 19.42(14.30 – 24.76) | –2.51 (–2.62 to –2.39) |
| 10–14 years old | 77.19(45.46 – 126.11) | 49.06(28.79 – 80.80) | –1.46 (–1.51 to –1.41) | 0.47 (0.40 – 0.58) | 0.23(0.15 – 0.29) | –2.02 (–2.2 to –1.84) | 42.60(36.76 – 50.71) | 21.15(15.26 – 25.77) | –2.03 (–2.19 to –1.87) |

**Supplementary Table 3.** Incidence cases, Mortality cases, and Disability-Adjusted Life Years of Childhood Poisoning in 1990 and 2021.

| Location | Incidence Cases(95%UI) | | Mortality Cases(95%UI) | | DALYs(95%UI) | |
| --- | --- | --- | --- | --- | --- | --- |
|  | 1990 | 2021 | 1990 | 2021 | 1990 | 2021 |
| Global | 1163111.48 (798334.75-1664230.44) | 822877.52 (550949.58-1197683.25) | 19283.64 (15278.57-24433.41) | 8681.59 (5638.47-12296.79) | 1722547.46 (1378810.36-2173390.45) | 782877.25 (528958.14-1102844.78) |
| Sex |  |  |  |  |  |  |
| Male | 546029 (375384.88-781681.64) | 389270.54 (261631.38-563153.73) | 11285.21 (8421.62-14445.23) | 4736.09 (2927.9-7106.4) | 1000469.99 (753327.2-1282798.74) | 423197.06 (272281.83-626403.9) |
| Female | 617082.48 (424638.65-883901.94) | 433606.98 (289256.04-632366.2) | 7998.44 (5183.41-10988.91) | 3945.5 (2207.92-5617.99) | 722077.48 (470600.17-983015.32) | 359680.18 (215961-502865.37) |
| SDI |  |  |  |  |  |  |
| High SDI | 321627.76 (223162.68-462479.09) | 203036.49 (136193.59-299811.79) | 498.9 (456.65-538.97) | 98.17 (88.97-109.46) | 60228.93 (53325.56-68570.57) | 20040.11 (15872.84-25555.79) |
| High-middle SDI | 327915.16 (232298.21-461558.36) | 185297.86 (125580.06-264388) | 3361.03 (2903.64-4472.73) | 628.2 (479.62-740.08) | 303495.82 (262980.32-399486.48) | 60453.03 (48459.11-70350.92) |
| Middle SDI | 276253.04 (184483-397126.58) | 189185.71 (124120.59-279198.49) | 5777.77 (4653.21-8619.86) | 1346.78 (794.42-1720.99) | 509730.46 (412755.83-754090.23) | 120733.95 (77081.36-153284.3) |
| Low-middle SDI | 154497.16 (102318.09-225606.61) | 133141.76 (87459.1-194811.66) | 3749.32 (2056.61-4780.84) | 1735.78 (1031.45-2673.18) | 331150.12 (186606.75-420012.58) | 154455.45 (95086.02-235914.99) |
| Low SDI | 80424.12 (54407.23-116484.11) | 110972.5 (75283.76-159296.01) | 5879.17 (3957.91-7768.61) | 4866.41 (3091.78-7458.22) | 516321.28 (349542.96-680307.21) | 426603.87 (272507.15-651368.22) |
| Region |  |  |  |  |  |  |
| Central Asia | 36408.01 (24540.83-53078.8) | 31072.35 (19547.78-45923.37) | 310.54 (266.33-356.27) | 106.73 (85.45-138.66) | 28410.1 (24313.88-32605.94) | 10207.53 (8320.44-13008.08) |
| Central Europe | 106731.99 (76085.28-149165.6) | 42227.11 (27463.13-63360.79) | 346.84 (316.76-376.68) | 17.2 (15.33-19.5) | 34512.08 (31515.21-37735.7) | 3099.72 (2499.02-3896.62) |
| Eastern Europe | 101189.51 (71207.72-143461.54) | 43276.94 (29398.32-61419.68) | 1021.54 (976.68-1070.01) | 193.34 (180.63-203.97) | 90835.51 (86459.72-95123.51) | 17266.91 (16090.1-18324.83) |
| Australasia | 7147.44 (4769.95-10658.43) | 7729.2 (5094.57-11374.65) | 4.33 (3.98-4.73) | 1.55 (1.36-1.74) | 794.71 (627.8-1011.06) | 607.2 (429.99-833.47) |
| High-income Asia Pacific | 70830.05 (48988.79-103133.43) | 30420.15 (19760.08-45843.42) | 96.52 (51.79-124.74) | 4.38 (3.73-6.13) | 12018.25 (8197.03-14697.18) | 2043.64 (1415.63-2846.51) |
| High-income North America | 112052.22 (74606.06-167458.2) | 73121.02 (48598.78-107641.34) | 96.43 (94.16-98.69) | 51.96 (47.17-56.59) | 14533.94 (12210.77-17554.07) | 8681.41 (7163.3-10563.26) |
| Southern Latin America | 56346.69 (39529.19-79713.04) | 47549.62 (32801.4-67627.11) | 101.97 (95.55-108.98) | 28.78 (24.82-32.98) | 12203.86 (10841.45-13790.21) | 5226.89 (4170.36-6562.08) |
| Western Europe | 121033.99 (83403.95-175127.53) | 91989.07 (63302.39-133313.68) | 104.63 (101.21-108.03) | 12.96 (11.98-14.01) | 16972.89 (14017.37-20642.88) | 7197.53 (4998.81-9909.38) |
| Andean Latin America | 15396.31 (10799.41-21384.23) | 12655.48 (8254.89-18684.41) | 199.85 (151.37-242.2) | 40.08 (29.7-56.02) | 18010.77 (13907.84-21624.61) | 3912.4 (2966.14-5310.66) |
| Caribbean | 12165.71 (8315.17-17562.84) | 10692.07 (7212.62-15664.19) | 187.93 (110.12-296.93) | 52.48 (29.39-94.97) | 17065.37 (10271.94-26498.33) | 5108.51 (3112.91-8913.29) |
| Central Latin America | 114455.31 (77611.85-164489.88) | 80747.7 (53346.71-120246.73) | 563.35 (519.81-611.37) | 92.07 (73.8-115.62) | 55265.31 (50914.09-60181.81) | 12354.82 (10109.96-15188.82) |
| Tropical Latin America | 26047.04 (16259.44-40536.73) | 14768.76 (9714.17-22045.99) | 102.03 (90.36-116.57) | 14.59 (11.64-17.73) | 10292.9 (9251.81-11624.29) | 2012.1 (1651.48-2466.31) |
| North Africa and Middle East | 52169.55 (35197.47-75460.36) | 42048.36 (26307.86-63601.96) | 2462.55 (1393.86-3303.76) | 801.08 (474.28-1243.1) | 214278.7 (122247.71-288264.35) | 69461.93 (41701.77-106885.8) |
| South Asia | 82666.69 (53094.5-123511.37) | 61795.24 (39076.45-94674.58) | 2211.87 (1023.45-3048.32) | 666.14 (413.59-996.87) | 192712.58 (93624.49-265546.94) | 58256 (37966.06-86781.98) |
| East Asia | 110638.57 (73768.88-162082.21) | 70471.33 (47043.61-103124.31) | 5305.93 (4090.47-9090.54) | 1111.27 (572.68-1490.81) | 459671.75 (354628.45-788142.28) | 94478.56 (50084.38-126582.07) |
| Oceania | 656.17 (442.98-948.44) | 1079.29 (756.44-1521.97) | 22.8 (12.46-35.78) | 29.53 (17.58-49.34) | 1923.44 (1060.67-2974.36) | 2497.98 (1497.22-4070.26) |
| Southeast Asia | 49051.19 (31451.92-73815.87) | 37193 (23313.7-56438.97) | 286.85 (167.95-427.62) | 132.3 (82.01-169.94) | 26611.61 (16298.82-39172.62) | 12638.41 (8780.97-15931.36) |
| Central Sub-Saharan Africa | 7684.94 (5182.97-11177.57) | 11237.98 (7470.36-16258.43) | 711.69 (441.3-981.63) | 513.81 (265.34-1173.05) | 62724.53 (38910.36-86563.53) | 45058.51 (23311.91-103224.33) |
| Eastern Sub-Saharan Africa | 39559.04 (27108.94-56913.13) | 47947.19 (32737.53-69622.17) | 3051.68 (2057.14-3929.47) | 2205.43 (1246.69-3807.65) | 269654.19 (182110.57-346010.97) | 194286.04 (111121.3-335304.49) |
| Southern Sub-Saharan Africa | 7503.63 (5153.49-10783.44) | 6015.53 (4140.89-8522.83) | 339.35 (224.56-428.43) | 290.39 (186.64-384.94) | 29509.64 (19588.93-37271.54) | 24948.48 (16092.68-32939.33) |
| Western Sub-Saharan Africa | 33377.44 (22444.37-48680.81) | 58840.12 (39846.45-84394.12) | 1754.98 (1127.89-2825.69) | 2315.53 (1439.63-3224.96) | 154545.31 (99527.99-248215.71) | 203532.69 (128734.25-282346.61) |

**Supplementary Table4.** Incidence Cases and Age–Standardized Incidence Rate (ASIR) of Childhood Poisoning in 204 Countries and Territories, 1990–2021

| location | Incidence cases_1990 | Incidence cases_2021 | ASIR_1990 | ASIR_2021 | EAPC |
| --- | --- | --- | --- | --- | --- |
| Afghanistan | 1251.73 (855.74–1836.94) | 2466.28 (1607.55–3737.49) | 30.16 (18.44–47.14) | 18.15 (10.83–29.03) | –1.6 (–1.75 to –1.46) |
| Albania | 2839.9 (1882.44–4052.93) | 1067.64 (718.13–1550.9) | 256.03 (152.7–401.7) | 236.86 (144.93–363.4) | –0.17 (–0.32 to –0.02) |
| Algeria | 4779.03 (3306.45–6534.22) | 3092.72 (1953.85–4563.84) | 44.69 (28.71–65.95) | 23.47 (13.97–36.91) | –1.99 (–2.08 to –1.89) |
| American Samoa | 5.11 (3.25–7.89) | 3.31 (2.03–5.13) | 27.64 (15.49–45.81) | 22.19 (12.37–36.5) | –0.74 (–0.95 to –0.52) |
| Andorra | 13 (8.79–18.91) | 11.57 (7.53–17.47) | 144.53 (90.44–227.09) | 122.22 (73.64–195.35) | –0.51 (–0.55 to –0.48) |
| Angola | 1619.81 (1103.56–2360.39) | 3026.56 (2032.55–4390.44) | 35.11 (21.58–54.06) | 20.13 (12.19–31.58) | –1.91 (–2.14 to –1.69) |
| Antigua and Barbuda | 21.55 (14.58–30.65) | 15.21 (9.9–22.47) | 117.9 (72.63–180.41) | 88.9 (51.29–141.53) | –0.88 (–0.94 to –0.82) |
| Argentina | 43713.57 (30970.25–61602.07) | 34478.06 (23393.49–49821.76) | 432.91 (282.42–647.93) | 340.07 (216.08–516.46) | –0.36 (–0.68 to –0.03) |
| Armenia | 1156.97 (719.27–1777.05) | 648.91 (401.45–1033.39) | 113.27 (62.55–186.38) | 105.93 (57.59–178.8) | –0.18 (–0.32 to –0.04) |
| Australia | 5537.05 (3647.65–8417.25) | 6123 (3961.35–9135.7) | 147.5 (87.74–236.41) | 130.65 (75.96–211.94) | –0.34 (–0.39 to –0.28) |
| Austria | 2253.4 (1603.7–3063) | 1906.72 (1317.71–2768.63) | 169.93 (108.09–253.47) | 149.05 (92.68–231.35) | –0.11 (–0.26 to 0.04) |
| Azerbaijan | 2950.26 (1878.64–4371.88) | 2613.02 (1585.32–3995.46) | 124.68 (72.14–195.86) | 105.87 (59.41–168.9) | –0.45 (–0.58 to –0.33) |
| Bahamas | 77.24 (50.66–115.86) | 68.19 (40.89–107.67) | 94.52 (54.62–151.78) | 81 (44.02–135.97) | –0.38 (–0.53 to –0.22) |
| Bahrain | 62.38 (39.52–94.15) | 94.98 (58.13–147.02) | 38.8 (21.9–63.94) | 31.45 (17.22–51.8) | –0.58 (–0.79 to –0.38) |
| Bangladesh | 8009.84 (5105.89–11808.85) | 5780.26 (3645.9–8676.13) | 16.79 (9.81–27.06) | 12.23 (7.1–19.43) | –1.06 (–1.19 to –0.93) |
| Barbados | 62.3 (40.98–92.75) | 43.42 (26.73–67.39) | 98.75 (57.34–157.93) | 90.39 (49.56–151.63) | –0.28 (–0.43 to –0.12) |
| Belarus | 4325.57 (2903.55–6270.53) | 2261.07 (1402.92–3427.59) | 177.59 (110.72–271.78) | 135.86 (80.06–213.11) | –0.95 (–1.16 to –0.74) |
| Belgium | 4578.84 (3305.1–6210.05) | 2836.94 (1922.27–4168.29) | 256.91 (170.47–379.49) | 152.11 (94.1–238.65) | –1.17 (–1.61 to –0.73) |
| Belize | 129.77 (95.28–178.03) | 94.4 (59.02–143.21) | 158.17 (107.21–226.41) | 75.03 (42.48–122.95) | –2.49 (–2.73 to –2.24) |
| Benin | 948.77 (636.04–1395.91) | 1940.35 (1308.59–2764.44) | 39.66 (24.22–62.43) | 32.24 (19.64–49.11) | –0.7 (–0.83 to –0.58) |
| Bermuda | 17.17 (11.72–24.51) | 8.53 (5.35–12.95) | 144.65 (90.27–220.23) | 99.41 (54.89–161.33) | –1.06 (–1.2 to –0.92) |
| Bhutan | 46.11 (28.65–69.47) | 25.21 (15.26–39.09) | 17.75 (10.05–28.99) | 13.17 (7.4–21.8) | –1.1 (–1.26 to –0.94) |
| Bolivia (Plurinational State of) | 2747.95 (1941.04–3853.34) | 2261.55 (1462.98–3370.35) | 103.98 (66.11–155.17) | 64.4 (37.68–102.06) | –1.64 (–1.68 to –1.6) |
| Bosnia and Herzegovina | 5200.4 (3896.37–7160.2) | 1389.75 (876.27–2088.97) | 466.57 (310.17–673.2) | 277.92 (156.22–449.02) | –1.61 (–1.8 to –1.41) |
| Botswana | 195.74 (134.05–281.69) | 259.62 (181.83–364.42) | 33.3 (20.49–51.62) | 37.14 (23.99–55.37) | 0.48 (0.41 to 0.56) |
| Brazil | 25142.04 (15575.85–39198.99) | 14019.93 (9253.43–20934.65) | 47.69 (26.54–79.18) | 29.02 (16.77–46.77) | –2.72 (–3.16 to –2.27) |
| Brunei Darussalam | 148.84 (102.03–213.08) | 122.36 (81–179.55) | 163.05 (100.56–253.01) | 130.04 (76.9–207.55) | –0.81 (–0.91 to –0.7) |
| Bulgaria | 5625.32 (3904.74–7874.3) | 2352.91 (1522.33–3489.77) | 321.96 (211.2–471.65) | 235.89 (137.29–377.94) | –1.09 (–1.16 to –1.02) |
| Burkina Faso | 1820.15 (1211.03–2651.07) | 3371.27 (2274.75–4789.51) | 38.9 (23.27–61.1) | 32.87 (20.03–49.38) | –0.6 (–0.66 to –0.55) |
| Burundi | 1136.3 (770.58–1637.02) | 1682.08 (1161.53–2412.67) | 44.42 (27.02–68.62) | 29.1 (18.27–45.09) | –1.43 (–1.57 to –1.28) |
| Cabo Verde | 60.2 (36.36–93.23) | 42.37 (25.92–65.03) | 38.72 (21.06–65.9) | 28.91 (15.65–48.03) | –0.88 (–1.07 to –0.69) |
| Cambodia | 1071.24 (690.95–1600.27) | 821.05 (499.98–1257.53) | 23.87 (13.93–37.49) | 15.89 (8.73–25.86) | –1.31 (–1.4 to –1.22) |
| Cameroon | 1584.2 (1046.29–2316.78) | 3640.64 (2459.56–5232.9) | 33.41 (19.91–52.72) | 27.17 (16.87–41.46) | –0.72 (–0.89 to –0.55) |
| Canada | 7845.63 (5262.79–11631.98) | 6680.22 (4390.94–9771.95) | 136.85 (82.74–212.48) | 109.27 (65.39–171.56) | –0.7 (–0.74 to –0.66) |
| Central African Republic | 342.12 (230.65–502.21) | 530.89 (360.54–760.99) | 28.69 (17.3–44.45) | 23.42 (14.43–34.94) | –0.69 (–0.78 to –0.6) |
| Chad | 1038.64 (684.06–1523.07) | 2825.6 (1966.93–3975.5) | 36.48 (22.21–56.83) | 32.03 (20.55–48.01) | –0.46 (–0.58 to –0.34) |
| Chile | 10212.13 (6844.85–14674.63) | 11332.19 (7777.91–15401.1) | 256.77 (155.57–399.74) | 313.17 (200.69–458.61) | 0.82 (0.72 to 0.91) |
| China | 107876.04 (71973.76–157894.54) | 68767.79 (45953.07–100650.31) | 33.86 (20.31–52.99) | 25.41 (15.63–39.44) | –0.57 (–0.77 to –0.36) |
| Colombia | 20751.79 (14091.29–29710.29) | 13778.85 (8923.67–21131.79) | 178.22 (110.46–272.2) | 130.56 (76.3–216.96) | –0.94 (–1.02 to –0.85) |
| Comoros | 81.21 (54.06–119.01) | 68.62 (45.17–99.73) | 38.86 (23.47–60.69) | 28.34 (17.21–43.81) | –1.13 (–1.26 to –0.99) |
| Congo | 295.78 (190.63–435.9) | 352.74 (224.25–533.58) | 28.38 (16.61–44.83) | 18.02 (10.42–29.39) | –1.52 (–1.68 to –1.36) |
| Cook Islands | 2 (1.27–3.12) | 1.06 (0.64–1.66) | 30.08 (16.91–50.54) | 26.83 (14.37–45.9) | –0.26 (–0.45 to –0.06) |
| Costa Rica | 1955.08 (1221.5–2918.79) | 1543.65 (992.95–2320.61) | 173.49 (99.8–276.15) | 152.18 (88.95–247.56) | –0.4 (–0.52 to –0.29) |
| Coted'Ivoire | 1975.52 (1318.35–2863.69) | 3355.76 (2251.08–4858) | 35.37 (21.46–55.39) | 29.32 (18.2–44.73) | –0.63 (–0.74 to –0.52) |
| Croatia | 2659.75 (1729.12–3840.02) | 1432.9 (928.3–2133.44) | 263.4 (156.55–414.72) | 236.75 (139.36–374.92) | 0.06 (–0.14 to 0.26) |
| Cuba | 2514.09 (1650.72–3726.04) | 2485.74 (1584.99–3856.5) | 101.03 (60.41–162.96) | 137.02 (76.71–225.18) | 1.51 (1.18 to 1.84) |
| Cyprus | 287.64 (198.97–413.06) | 261.82 (175.71–391.61) | 148.71 (93.57–229.84) | 120.31 (72.47–190.41) | –0.52 (–0.61 to –0.43) |
| Czechia | 9306.4 (6315.34–13567.99) | 5497.56 (3555.03–8232.33) | 409.72 (253.28–634.97) | 317.93 (177.68–512.84) | –0.35 (–0.61 to –0.09) |
| Democratic People's Republic of Korea | 1371.8 (904.35–2017.09) | 888.3 (572.12–1316) | 23.44 (13.71–36.51) | 17.84 (10.41–28.16) | –0.71 (–0.82 to –0.61) |
| Democratic Republic of the Congo | 5240.61 (3493.77–7647.48) | 7095.58 (4626.83–10289.48) | 30.22 (17.95–46.63) | 18.77 (11.35–28.83) | –1.59 (–1.77 to –1.4) |
| Denmark | 1188.06 (803.66–1728.71) | 1203.89 (810.32–1795.25) | 137.58 (84.58–213.16) | 128.52 (77.75–204.55) | –0.19 (–0.24 to –0.14) |
| Djibouti | 67.73 (44.31–97.66) | 120.96 (80.73–177.25) | 39.28 (23.18–60.75) | 29.32 (17.65–45.15) | –1.03 (–1.2 to –0.85) |
| Dominica | 46.14 (33.61–63.02) | 15.9 (10.83–22.75) | 186.1 (124.81–267.42) | 112.42 (69.59–166.97) | –1.75 (–1.99 to –1.5) |
| Dominican Republic | 2650.2 (1809.01–3820.29) | 2296.92 (1513.83–3497.07) | 99.29 (60.79–151.96) | 78.24 (46.4–126.38) | –0.65 (–0.75 to –0.54) |
| Ecuador | 3416.54 (2327.93–4921.21) | 3694.2 (2340.17–5613.27) | 88.24 (54.58–135.98) | 71.58 (40.76–115) | –0.58 (–0.67 to –0.48) |
| Egypt | 5449.65 (3292.71–8396.88) | 7839.81 (4592.43–12139.35) | 25.37 (14.39–41.76) | 21.4 (11.39–35.58) | –0.43 (–0.65 to –0.2) |
| El Salvador | 3360.86 (2132.53–5002.93) | 2459.71 (1513.89–3757.5) | 156.46 (90.29–247.6) | 135.1 (76.06–220.33) | –0.45 (–0.57 to –0.33) |
| Equatorial Guinea | 63.35 (41.65–92.08) | 105.51 (65.66–161.42) | 33.16 (20.15–52.2) | 17.64 (9.95–29.11) | –2.2 (–2.36 to –2.03) |
| Eritrea | 640.11 (420.67–926.81) | 752.82 (501.71–1097.13) | 40.94 (24.85–64.08) | 30.05 (18.72–46.4) | –1.1 (–1.23 to –0.97) |
| Estonia | 593.87 (388.96–892.02) | 392.77 (242.31–612.28) | 169.1 (101.3–265.2) | 175.9 (96.47–289.72) | 0.37 (0.17 to 0.56) |
| Eswatini | 139.31 (96.46–197.67) | 125.25 (87.99–174.79) | 36.29 (22.73–55.38) | 30.26 (19.5–44.68) | –0.48 (–0.55 to –0.4) |
| Ethiopia | 13597.77 (9537.04–19380.78) | 11342.82 (7689.66–16462.5) | 56.7 (34.73–86.76) | 25.69 (16.01–39.72) | –2.73 (–2.87 to –2.59) |
| Fiji | 68.61 (42.6–106.02) | 51.86 (32.65–80.28) | 24.17 (13.64–39.43) | 18.82 (10.52–31.18) | –0.82 (–1.05 to –0.59) |
| Finland | 1196.76 (804.21–1743.02) | 907.66 (593.96–1382) | 126.75 (76.67–199.53) | 112.03 (67.03–178.58) | –0.51 (–0.78 to –0.24) |
| France | 17166.64 (11769.12–25315.58) | 14960.83 (10008.84–22130.75) | 148.43 (91.53–231.79) | 132.68 (81.14–207.23) | –0.21 (–0.29 to –0.14) |
| Gabon | 123.26 (81.47–176.18) | 126.69 (79.91–194.74) | 30.63 (18.19–48.17) | 19.62 (10.93–32.24) | –1.39 (–1.52 to –1.26) |
| Gambia | 173.63 (116.05–257.32) | 255.01 (167.37–380.04) | 38.36 (22.76–61.6) | 25.83 (15.54–40.2) | –1.42 (–1.63 to –1.21) |
| Georgia | 2242.6 (1406.68–3417.64) | 1304.26 (874.62–1917.4) | 162.35 (93.9–256.09) | 174.65 (105.98–270.75) | 0.71 (0.49 to 0.94) |
| Germany | 17306.3 (11644.13–25846.25) | 14748.34 (9846.92–22091.02) | 134.16 (81.16–213.85) | 124.24 (73.87–198.82) | –0.19 (–0.24 to –0.14) |
| Ghana | 2400.82 (1628.22–3486.63) | 3510.81 (2376.9–5128.84) | 36.32 (21.85–55.77) | 27.39 (16.84–42.4) | –0.9 (–0.99 to –0.8) |
| Greece | 2598.37 (1732.46–3858.34) | 1574.55 (1067.7–2335.32) | 135.94 (84.06–216.07) | 116.85 (71–185.74) | –0.43 (–0.49 to –0.37) |
| Greenland | 22.62 (16.57–30.51) | 10.7 (7.34–15.3) | 158.36 (106.05–229.88) | 91.11 (56.78–139.41) | –2.11 (–2.23 to –1.98) |
| Grenada | 47.91 (33.71–67.33) | 18.43 (11.65–28.14) | 143.49 (91.28–213.73) | 83.19 (46.26–135.65) | –1.54 (–1.78 to –1.29) |
| Guam | 11.99 (7.6–18.18) | 9.15 (5.66–14.24) | 29.31 (16.43–48.4) | 24.91 (13.66–42.2) | –0.51 (–0.7 to –0.32) |
| Guatemala | 7631.89 (5232.09–10937.52) | 7395.59 (5030.8–10492.67) | 188.38 (116.92–290.59) | 148.41 (93.08–223.57) | –0.62 (–0.71 to –0.52) |
| Guinea | 1139.3 (754.63–1685) | 2036.34 (1402.1–2916.66) | 42.05 (25.06–65.27) | 33.87 (21.5–51.92) | –0.73 (–0.79 to –0.67) |
| Guinea–Bissau | 190.13 (129.91–275.04) | 226.43 (155.83–320.54) | 39.83 (24.3–61.33) | 25.47 (16.07–39.15) | –1.56 (–1.73 to –1.39) |
| Guyana | 252.5 (166.71–358.82) | 158.54 (102.28–242.61) | 87.7 (53.64–137.04) | 74.28 (41.95–122.11) | –0.45 (–0.61 to –0.3) |
| Haiti | 3112.39 (2184.15–4517.2) | 3681.51 (2590.58–5222.37) | 115.81 (73.9–174.77) | 85 (54.24–125.59) | –0.98 (–1.09 to –0.87) |
| Honduras | 4608.92 (3292.52–6400.19) | 4591.08 (3020.4–6823.43) | 207.9 (136.91–303.53) | 140.53 (83.29–222.86) | –1.18 (–1.27 to –1.09) |
| Hungary | 6495.47 (4268.43–9702.25) | 3730.62 (2383.69–5597.92) | 297.66 (178.33–470.86) | 266.16 (154.55–432.39) | –0.21 (–0.32 to –0.09) |
| Iceland | 99.31 (67.54–143.42) | 88.65 (60.57–129.99) | 158.45 (97.74–247.17) | 133.66 (81.57–207.86) | –0.44 (–0.55 to –0.34) |
| India | 62116.27 (39769.24–93913.02) | 41429.99 (25703.27–64659.57) | 19.13 (11.27–30.83) | 10.85 (6.15–17.91) | –1.91 (–2.13 to –1.7) |
| Indonesia | 17150.27 (10360.21–26842.52) | 12998.51 (7839.73–20189.71) | 24.82 (13.88–41.44) | 18.76 (10.41–31.3) | –0.83 (–1.12 to –0.54) |
| Iran (Islamic Republic of) | 12515.86 (8611.85–17827.57) | 3743.68 (2338.91–5660.09) | 49.55 (31.18–75.84) | 17.9 (10.37–28.59) | –3.49 (–3.82 to –3.15) |
| Iraq | 2162.66 (1388.35–3319.67) | 3117.55 (1889.47–4790.35) | 27.04 (15.21–44.89) | 22.63 (12.32–37.71) | –0.45 (–0.66 to –0.25) |
| Ireland | 1409.9 (945.17–2069.22) | 1289.78 (858.72–1915.26) | 148.73 (90.38–230.8) | 133.29 (80.72–211.8) | –0.32 (–0.35 to –0.29) |
| Israel | 2270.68 (1557.01–3248.77) | 3187.64 (2164.19–4695.43) | 149.8 (93.25–230.89) | 121.28 (73.79–193.31) | –0.48 (–0.57 to –0.39) |
| Italy | 20488.12 (13551.54–30610.82) | 9863.08 (7162.91–13475.44) | 228.63 (138.57–363.04) | 134.05 (88.21–197.8) | –2.12 (–2.51 to –1.73) |
| Jamaica | 931.38 (600.53–1402.79) | 561.69 (352.19–864.71) | 110.85 (62.52–180.96) | 94.51 (51.56–154.88) | –0.35 (–0.5 to –0.21) |
| Japan | 43514.67 (28667.13–64540.84) | 21605.55 (14000.02–32469.92) | 188.56 (112.15–300.07) | 139.37 (80.68–224.92) | –0.94 (–1.1 to –0.77) |
| Jordan | 697.49 (481.13–958.82) | 967.49 (613.02–1455.66) | 43.5 (27.59–63.71) | 25.41 (15.01–40.55) | –1.92 (–2.12 to –1.71) |
| Kazakhstan | 10025.94 (7176.86–14129.59) | 7210.05 (4856.57–10213.79) | 194.82 (129.98–283.6) | 134.28 (84.99–200.19) | –1.47 (–1.72 to –1.23) |
| Kenya | 3564.78 (2300.81–5412) | 4109.88 (2690.73–6231.51) | 32.67 (18.76–53.11) | 21.44 (12.48–34.24) | –2.03 (–2.35 to –1.7) |
| Kiribati | 9.53 (6.65–13.36) | 11.2 (8–15.87) | 34.09 (21.81–51.51) | 26.39 (17.38–38.8) | –0.66 (–0.77 to –0.56) |
| Kuwait | 201.4 (131.35–297.12) | 242.66 (156.61–358.36) | 36.85 (21.53–59.21) | 27.93 (15.76–45.61) | –0.88 (–1.03 to –0.73) |
| Kyrgyzstan | 2465.8 (1674.73–3544.13) | 2399.81 (1481.16–3625.92) | 151.58 (93.99–228.75) | 106.03 (61.31–167.51) | –1.05 (–1.15 to –0.96) |
| Lao People's Democratic Republic | 509.24 (334.1–755.44) | 365.37 (227.4–562.25) | 28.44 (16.96–44.33) | 16.07 (9.07–26.09) | –1.88 (–1.97 to –1.8) |
| Latvia | 900.99 (577.45–1350.02) | 495.01 (299.49–778.41) | 159.79 (95.46–247.68) | 160.93 (87.18–267.75) | 0.28 (0.11 to 0.46) |
| Lebanon | 360.25 (233.14–543.11) | 359.87 (226.62–554.42) | 34.93 (20.17–57.02) | 27.46 (15.58–44.7) | –0.78 (–0.94 to –0.63) |
| Lesotho | 212.16 (147.55–299.87) | 194.4 (141.49–269.78) | 31.22 (19.71–47.86) | 30.57 (19.99–45.28) | 0.06 (–0.01 to 0.13) |
| Liberia | 433 (288.24–633.9) | 513.93 (340.73–770.98) | 38.77 (23.38–60.67) | 23.52 (14.28–36.8) | –1.76 (–1.83 to –1.68) |
| Libya | 666.25 (443.22–973.47) | 386.41 (241.77–583.98) | 36.79 (22.16–57.11) | 24.39 (14.06–38.78) | –1.29 (–1.41 to –1.16) |
| Lithuania | 1551.43 (1072.84–2277.42) | 584.53 (358.51–908.65) | 186.36 (119.04–279.28) | 139.22 (79.63–219.68) | –0.87 (–1.02 to –0.72) |
| Luxembourg | 102.94 (70.42–147.57) | 122.91 (81.99–183.67) | 156.24 (97.61–241.02) | 123.37 (73.15–196.78) | –0.62 (–0.72 to –0.52) |
| Madagascar | 2449.21 (1665.64–3501.02) | 2984.31 (1969.08–4358.54) | 45.95 (28.48–70.42) | 25.42 (15.08–39.5) | –1.86 (–1.97 to –1.75) |
| Malawi | 1908.51 (1321.34–2716.49) | 2134.3 (1438.46–3172.06) | 42.8 (26.21–66.94) | 26 (15.89–39.81) | –1.73 (–1.83 to –1.63) |
| Malaysia | 1902.5 (1137.96–2985.53) | 1753.32 (1043.49–2737.31) | 29.25 (15.43–49.76) | 22.52 (11.85–37.78) | –0.89 (–1.01 to –0.77) |
| Maldives | 26.5 (16.3–41.1) | 25.33 (14.84–40.26) | 26.43 (14.1–44.78) | 24.59 (12.87–42.5) | –0.18 (–0.36 to 0) |
| Mali | 1766.53 (1215.79–2602.51) | 4026.56 (2759.78–5804.54) | 43.38 (26.29–67.95) | 35.22 (21.68–53.44) | –0.72 (–0.75 to –0.69) |
| Malta | 126.4 (86.64–185.75) | 82.35 (54.48–122.36) | 147.52 (89.74–230.03) | 129.2 (77.06–205.8) | –0.11 (–0.3 to 0.08) |
| Marshall Islands | 5.55 (3.64–8.15) | 3.57 (2.35–5.27) | 25.27 (14.91–39.55) | 19.94 (11.88–31.54) | –0.81 (–0.98 to –0.65) |
| Mauritania | 294.39 (194.42–437.53) | 443.08 (278.52–649.5) | 32.66 (19.43–52.52) | 23.98 (13.75–38.83) | –1.07 (–1.24 to –0.91) |
| Mauritius | 92.56 (56.89–142.97) | 52.17 (32.81–79.54) | 27.46 (14.93–45.57) | 24.44 (13.92–39.6) | –0.37 (–0.44 to –0.3) |
| Mexico | 57361.2 (38627.92–81776.44) | 36341.04 (23846.5–53850.98) | 172.05 (105.61–267.48) | 112.24 (66.02–179.73) | –0.78 (–1.42 to –0.14) |
| Micronesia (Federated States of) | 12.48 (8.38–17.93) | 6.27 (3.99–9.32) | 27.02 (16.34–42.25) | 19.71 (11.55–31.06) | –1.04 (–1.18 to –0.9) |
| Monaco | 8.28 (5.97–11.3) | 6.73 (4.49–9.9) | 237.44 (155.06–351.03) | 136.94 (83.11–216.58) | –1.65 (–1.92 to –1.38) |
| Mongolia | 2381.69 (1777.64–3191.57) | 1260.67 (838.97–1760.03) | 271.58 (186.29–376.45) | 119.26 (74.14–178.25) | –2.89 (–3.1 to –2.67) |
| Montenegro | 475.39 (313.03–713.54) | 288.2 (185.4–435.18) | 290.78 (174.58–460.17) | 255.6 (145.81–411.17) | –0.25 (–0.37 to –0.13) |
| Morocco | 3237.36 (2178.8–4669.85) | 2228.17 (1400.96–3334.58) | 33.43 (20.24–51.76) | 22.31 (12.84–35.59) | –1.34 (–1.47 to –1.21) |
| Mozambique | 2376.53 (1630.76–3458.37) | 3418.79 (2329.99–4907.71) | 38.71 (23.44–60.82) | 24.24 (14.75–37.11) | –1.6 (–1.69 to –1.51) |
| Myanmar | 8150.68 (5832.04–11541.24) | 6464.72 (4804.61–9006.34) | 54.79 (35.38–80.35) | 41.01 (27.14–60.61) | –1.04 (–1.11 to –0.97) |
| Namibia | 199.2 (134.5–290.13) | 230.01 (155.68–331.61) | 33.4 (20.2–52.34) | 27.74 (17.41–42.69) | –0.47 (–0.57 to –0.36) |
| Nauru | 1.1 (0.73–1.59) | 0.81 (0.55–1.19) | 26.94 (16.69–41.61) | 20.47 (12.58–31.93) | –1.03 (–1.24 to –0.82) |
| Nepal | 2616 (1845.29–3597.13) | 1945.79 (1329.01–2768.87) | 32.14 (20.48–47.03) | 20.74 (13.01–31.04) | –1.47 (–1.54 to –1.39) |
| Netherlands | 3735.66 (2570.11–5395.97) | 3130.58 (2081.78–4610.61) | 138.15 (86.08–214.98) | 119.46 (71.94–190.29) | 0.1 (–0.34 to 0.54) |
| New Zealand | 1610.39 (1110.85–2280.53) | 1606.21 (1143.5–2227) | 201.94 (125.6–311.39) | 167.45 (109.31–246.64) | –0.67 (–0.76 to –0.58) |
| Nicaragua | 3483.35 (2365.66–5011.03) | 2699.54 (1721.98–3929.74) | 189.97 (118.59–292.5) | 135.91 (80.52–214.08) | –1.11 (–1.14 to –1.07) |
| Niger | 1610.79 (1052.96–2419.45) | 3909.57 (2652.39–5617.66) | 40.32 (24.07–63.91) | 31.22 (18.54–48.21) | –0.92 (–1.03 to –0.81) |
| Nigeria | 15073.7 (10046.92–22139.26) | 25136.16 (16873.41–36435.06) | 39.5 (23.63–62.54) | 24.93 (15.19–38.59) | –1.57 (–1.78 to –1.36) |
| Niue | 0.23 (0.15–0.34) | 0.09 (0.06–0.14) | 28.47 (16.95–44.82) | 22.93 (13.77–36.96) | –0.74 (–0.88 to –0.61) |
| North Macedonia | 1416.62 (925.57–2099.63) | 823.17 (532.99–1267) | 264.87 (155.78–420.41) | 244.92 (143.97–395.41) | –0.09 (–0.25 to 0.06) |
| Northern Mariana Islands | 3.55 (2.23–5.54) | 2.94 (1.77–4.59) | 29.72 (16.27–50.57) | 24.95 (13.36–41.98) | –0.63 (–0.82 to –0.44) |
| Norway | 2909.25 (2172.75–3876.75) | 4513.9 (3375.68–5932.03) | 367.75 (248.48–537.33) | 510.67 (357.14–707.34) | 1.19 (0.8 to 1.57) |
| Oman | 306.1 (200.08–452.45) | 322.15 (195.95–487.09) | 37.49 (22.1–60.28) | 26.48 (14.64–43.57) | –1.07 (–1.19 to –0.96) |
| Pakistan | 9878.46 (6351.1–15146.09) | 12614 (8605.04–18213.89) | 20.32 (11.84–32.98) | 14.75 (9.05–22.6) | –1.19 (–1.35 to –1.02) |
| Palau | 2.56 (1.83–3.58) | 1.38 (0.99–1.89) | 54.74 (35.61–81.32) | 39.59 (26.58–57.28) | –0.81 (–0.92 to –0.69) |
| Palestine | 295.52 (177.5–460.67) | 516.92 (301.9–839.06) | 32.11 (16.92–55.11) | 27.18 (14.21–47.49) | –0.45 (–0.73 to –0.16) |
| Panama | 1531.78 (1031.98–2262) | 1628.65 (1045.68–2396.56) | 183.97 (114.27–283.65) | 141.25 (83.93–223.78) | –0.97 (–1.11 to –0.83) |
| Papua New Guinea | 399.17 (268.98–579.11) | 813.92 (567.36–1146.42) | 24.19 (14.71–36.94) | 21.61 (13.55–32.27) | –0.42 (–0.58 to –0.26) |
| Paraguay | 905 (607.66–1323.08) | 748.83 (471.25–1127.14) | 54.37 (32.56–85.33) | 36.98 (20.39–60.24) | –1.21 (–1.27 to –1.15) |
| Peru | 9231.82 (6505.8–12821.47) | 6699.73 (4393.69–10169.84) | 111.38 (71.18–164.75) | 69.98 (40.67–111) | –1.45 (–1.51 to –1.4) |
| Philippines | 6745.13 (4184.37–10338.18) | 6877.64 (4204.2–10758.09) | 27.14 (15.31–44.68) | 19.73 (10.78–32.89) | –0.41 (–0.77 to –0.04) |
| Poland | 29717.22 (20286.35–42249.19) | 10338 (6532.69–15881.17) | 299.84 (191.42–449.17) | 170.94 (96.93–277.75) | –1.63 (–1.8 to –1.45) |
| Portugal | 3747.33 (2599.42–5376.17) | 1782.09 (1210.45–2571.09) | 187.14 (120.13–285.55) | 134.76 (82.99–209.2) | –0.84 (–0.98 to –0.7) |
| Puerto Rico | 1236.25 (808.51–1865.7) | 486.1 (292.96–759.82) | 123.7 (70.68–196.26) | 106.17 (56.72–175.59) | –0.5 (–0.59 to –0.41) |
| Qatar | 41.2 (25.96–62.43) | 136.57 (84.1–209.33) | 34.16 (18.83–56.38) | 28.16 (15.54–47.57) | –0.54 (–0.71 to –0.37) |
| Republic of Korea | 26095.32 (18997.2–35616.02) | 7462.06 (4714.95–11303.21) | 229.81 (150.03–341.04) | 124.78 (72.67–200.33) | –2.12 (–2.29 to –1.96) |
| Republic of Moldova | 3158.78 (2386.81–4125.49) | 897.88 (647.88–1247.03) | 255.05 (180.34–349.63) | 163.4 (110.62–236.19) | –1.68 (–1.79 to –1.57) |
| Romania | 29570.5 (22444.95–38954.19) | 7856.51 (5378.16–11114.73) | 534.53 (376.78–738.19) | 255.85 (159.99–382.77) | –2.63 (–2.76 to –2.5) |
| Russian Federation | 75860.85 (54263.83–106510.66) | 32147.23 (22365.54–44959.05) | 215.75 (138.81–319.54) | 115.47 (75.58–167.24) | –2.24 (–2.42 to –2.05) |
| Rwanda | 1422.4 (971.95–2036.86) | 1337.15 (894.35–1950.77) | 42.75 (26.43–66.22) | 26.93 (16.08–41.65) | –1.63 (–1.8 to –1.46) |
| Saint Kitts and Nevis | 14.18 (9.58–20.76) | 8.35 (5.26–13.1) | 99.84 (61.61–154.09) | 83.46 (46.09–137.43) | –0.42 (–0.53 to –0.3) |
| Saint Lucia | 51.89 (34.9–74.24) | 25.71 (16.04–39.64) | 100.49 (60.89–156.98) | 84.8 (46.29–142.54) | –0.43 (–0.57 to –0.29) |
| Saint Vincent and the Grenadines | 38.09 (25.19–57.57) | 20.83 (12.76–32.56) | 91.22 (54.21–146.16) | 81.1 (43.94–136.43) | –0.18 (–0.34 to –0.02) |
| Samoa | 19.03 (12.46–28.15) | 16.19 (10.15–24.31) | 26.76 (16.16–42.41) | 20.52 (11.66–33.33) | –0.82 (–0.98 to –0.66) |
| San Marino | 7.55 (5.36–10.5) | 6.56 (4.51–9.35) | 195.72 (126.37–295.9) | 157.92 (99.73–244.39) | –0.7 (–0.84 to –0.56) |
| Sao Tome and Principe | 24.06 (16.18–34.71) | 22.12 (13.48–33.54) | 42.56 (25.98–65.22) | 27.95 (15.72–46.08) | –1.32 (–1.42 to –1.23) |
| Saudi Arabia | 2277.77 (1505.01–3358.83) | 1829.61 (1125.51–2872.28) | 35.3 (20.81–56.45) | 23.62 (12.72–39.67) | –1.34 (–1.45 to –1.23) |
| Senegal | 1515.6 (1004.99–2213.54) | 1686.04 (1117.28–2458.72) | 42.05 (25.5–65.15) | 26.62 (16.44–40.84) | –1.61 (–1.76 to –1.47) |
| Serbia | 6538.38 (4391.71–9434.71) | 3671.74 (2303.57–5614.28) | 297.11 (183.23–455.01) | 268.79 (152.09–434.48) | –0.04 (–0.18 to 0.1) |
| Seychelles | 7.26 (4.51–11.2) | 5.61 (3.43–8.71) | 30.35 (16.45–50.86) | 23.73 (12.95–39.53) | –0.85 (–0.99 to –0.71) |
| Sierra Leone | 687.21 (456.41–1027.85) | 1067.03 (734.53–1509.15) | 38.54 (22.98–61) | 30.08 (18.61–45.24) | –0.84 (–0.94 to –0.73) |
| Singapore | 1071.21 (705.37–1563.6) | 1230.19 (819.02–1818.19) | 166.77 (99.09–265.07) | 151.32 (90.75–238.13) | –0.32 (–0.38 to –0.27) |
| Slovakia | 3899.97 (2635.13–5740.61) | 2215.05 (1448.32–3327.59) | 287.89 (171.63–455.77) | 256.8 (148–409.01) | –0.28 (–0.39 to –0.16) |
| Slovenia | 1279.81 (832.94–1895.84) | 948.42 (611.46–1444.89) | 302.6 (173.98–484.49) | 298.91 (169.83–481.6) | 0.15 (–0.2 to 0.5) |
| Solomon Islands | 41.57 (29.14–58.49) | 74.23 (52.43–103.81) | 27.5 (17.44–41.68) | 29.04 (18.61–43.39) | 0.25 (0.08 to 0.41) |
| Somalia | 1452.45 (960.86–2131.65) | 3100.04 (2138.01–4300.97) | 38.03 (22.92–59.84) | 30.9 (19.53–46.79) | –0.69 (–0.77 to –0.6) |
| South Africa | 5063.14 (3460.53–7285.74) | 3291.13 (2196.02–4714.1) | 37.29 (23.25–57.65) | 21.55 (13.11–33.41) | –1.87 (–2.07 to –1.68) |
| South Sudan | 1064.74 (721.57–1554.92) | 1228.46 (818.46–1779.51) | 41.05 (24.26–63.84) | 28.83 (17.49–44.47) | –1.19 (–1.33 to –1.04) |
| Spain | 11868.59 (8084.69–17312.82) | 8051.58 (5397.52–11963.51) | 161.18 (101.45–250.43) | 129.47 (78.29–204.43) | –0.65 (–0.71 to –0.59) |
| Sri Lanka | 1664.34 (1076.27–2499.68) | 1112.89 (677.18–1749.39) | 29.44 (16.95–47.23) | 21.12 (11.31–35.99) | –1.01 (–1.14 to –0.89) |
| Sudan | 3673.02 (2479.73–5434.86) | 4429.81 (2935.96–6463.08) | 42.23 (26.05–67.65) | 26.46 (16.16–41.2) | –1.56 (–1.66 to –1.47) |
| Suriname | 131.28 (90.07–192.49) | 106.25 (65.89–163.6) | 100.11 (61.56–154.3) | 72.56 (40.43–119.01) | –0.91 (–1.02 to –0.79) |
| Sweden | 3770.28 (2694.02–5287.34) | 3085.79 (2098.42–4447.14) | 241.89 (151.4–369.06) | 172.47 (105.91–269.21) | –1.09 (–1.24 to –0.94) |
| Switzerland | 1411.62 (951.19–2077.29) | 1615.16 (1094.08–2400.05) | 122.77 (74.96–194.53) | 122.6 (74.13–196.05) | 0.69 (0.42 to 0.96) |
| Syrian Arab Republic | 1849.74 (1193.09–2693.82) | 935.42 (600.02–1411.14) | 31.62 (18.9–49.54) | 23.58 (14.03–37.09) | –0.94 (–1.05 to –0.83) |
| Taiwan (Province of China) | 1390.73 (890.85–2100.27) | 815.24 (488.79–1314.56) | 24.35 (13.7–39.3) | 26.94 (14.18–46.26) | 0.54 (0.32 to 0.75) |
| Tajikistan | 2726.08 (1745.62–4037.63) | 3693.78 (2258.49–5844.57) | 125.38 (74.39–198.6) | 106.23 (58.12–176.1) | –0.38 (–0.5 to –0.27) |
| Thailand | 4077.99 (2568.13–6312.1) | 1848.38 (1098.5–2919.9) | 23.33 (12.96–38.13) | 17.92 (9.48–30.39) | –0.79 (–0.94 to –0.64) |
| Timor–Leste | 78.97 (49.65–118.66) | 84.7 (52.49–133.58) | 25.3 (14.32–40.91) | 16.27 (8.84–27.03) | –1.37 (–1.49 to –1.25) |
| Togo | 639.7 (425.72–924.01) | 830.38 (554.12–1214.94) | 36.79 (22.2–57.63) | 25.15 (15.16–38.86) | –1.35 (–1.49 to –1.21) |
| Tokelau | 0.18 (0.12–0.26) | 0.09 (0.06–0.14) | 29.59 (17.78–46.56) | 22.11 (12.47–36.68) | –0.9 (–1.06 to –0.74) |
| Tonga | 14.28 (9.92–19.93) | 10.96 (7.8–15.74) | 34.51 (21.97–51.97) | 28.64 (18.5–43.27) | –0.76 (–0.9 to –0.62) |
| Trinidad and Tobago | 383.51 (250.05–566.56) | 221.04 (137.96–341.28) | 93.32 (55.83–149.39) | 79.12 (43.21–133.81) | –0.49 (–0.62 to –0.37) |
| Tunisia | 1283.59 (866.61–1845.04) | 701.84 (445.75–1057.37) | 41.19 (25.29–63.19) | 24.83 (14.29–39.86) | –1.65 (–1.76 to –1.55) |
| Turkey | 8261.38 (5460.32–12144.9) | 5030.08 (3210.59–7650.7) | 39.95 (24.13–62.7) | 25.87 (14.81–41.27) | –1.42 (–1.52 to –1.32) |
| Turkmenistan | 1949.87 (1311.37–2846.07) | 1668.15 (1031.89–2493.81) | 135.27 (82.32–208.04) | 110.12 (64.83–171.92) | –0.55 (–0.66 to –0.44) |
| Tuvalu | 0.88 (0.6–1.25) | 0.66 (0.42–1.02) | 27.56 (17.35–41.82) | 17.59 (10.03–29) | –1.37 (–1.48 to –1.25) |
| Uganda | 3049.48 (2029.19–4531.3) | 5365.41 (3564.84–7715.93) | 37.67 (21.91–60.31) | 27.4 (16.63–42.4) | –1.08 (–1.23 to –0.93) |
| Ukraine | 14798.01 (9578.66–22035.54) | 6498.44 (4150.63–9700.73) | 127.34 (77.17–198.39) | 90.08 (54.75–137.2) | –1.15 (–1.27 to –1.02) |
| United Arab Emirates | 200.98 (122.86–317.2) | 399.32 (239.75–644.42) | 35.24 (18.81–59.85) | 29.45 (15.87–50.71) | –0.51 (–0.68 to –0.35) |
| United Kingdom | 22389.59 (15691.65–31344.05) | 16668.96 (11253.77–24328.12) | 204.88 (128.47–313.48) | 144.01 (87.26–223.79) | –1.25 (–1.29 to –1.2) |
| United Republic of Tanzania | 5175 (3497.43–7426.81) | 8103.03 (5476.65–11757.05) | 43.66 (26.4–67.35) | 33.4 (20.27–51.86) | –0.82 (–0.93 to –0.72) |
| United States of America | 104181.4 (69137.02–156450.44) | 66428.95 (44293.25–98317.97) | 186.2 (111.72–296.72) | 112.29 (67.94–176.66) | –1.87 (–3.18 to –0.55) |
| United States Virgin Islands | 35.91 (23.63–53.32) | 13.47 (8.54–20.3) | 112.38 (66.9–179.18) | 98.67 (54.8–161.51) | –0.31 (–0.45 to –0.18) |
| Uruguay | 2418.31 (1636.27–3465.89) | 1736.74 (1150.2–2571.51) | 296.65 (181.51–457.6) | 262.96 (156.62–416.75) | –0.37 (–0.47 to –0.27) |
| Uzbekistan | 10508.79 (6599.34–15996.05) | 10273.7 (6410.1–15045.1) | 128.54 (75.3–201.51) | 105.64 (61.96–167.09) | –0.49 (–0.6 to –0.37) |
| Vanuatu | 16.5 (10.86–24.31) | 23.14 (15.52–34.4) | 25.36 (15.22–40.06) | 20.13 (12.02–31.58) | –0.73 (–0.87 to –0.59) |
| Venezuela (Bolivarian Republic of) | 13770.43 (9124.02–20326.32) | 10309.59 (6856.12–15203.9) | 194.56 (117.5–301.85) | 156.17 (93.56–247.63) | –0.76 (–0.88 to –0.63) |
| Viet Nam | 7503.55 (4871.81–11047.7) | 4731.45 (2938.94–7301.27) | 28.5 (16.67–45.12) | 18.83 (10.53–31.37) | –1.45 (–1.56 to –1.34) |
| Yemen | 2567.65 (1710.87–3775.82) | 3167.8 (2101.29–4554.68) | 37.2 (22.7–57.5) | 22.77 (13.99–34.53) | –1.59 (–1.66 to –1.51) |
| Zambia | 1544.54 (1044.15–2260.71) | 2156.78 (1450.72–3212.56) | 41.86 (25.4–65.08) | 26.15 (15.95–40.94) | –1.61 (–1.75 to –1.47) |
| Zimbabwe | 1694.08 (1165.29–2448.77) | 1915.12 (1344.38–2650.84) | 35.21 (21.94–54.57) | 30.44 (19.34–45.32) | –0.47 (–0.56 to –0.37) |

**Supplementary Table 5.** Mortality Cases and Age–Standardized Mortality Rate (ASMR) of Childhood Poisoning in 204 Countries and Territories, 1990–2021

| location | Death cases_1990 | Death cases_2021 | ASMR_1990 | ASMR_2021 | EAPC |
| --- | --- | --- | --- | --- | --- |
| Afghanistan | 209.79 (82.82–371.66) | 230 (112.53–430.05) | 4.53 (1.68–8.94) | 1.56 (0.71–3.08) | –3.32 (–3.63 to –3.02) |
| Albania | 8.98 (4.73–12.99) | 0.73 (0.43–1.21) | 0.79 (0.39–1.21) | 0.17 (0.09–0.3) | –4.93 (–5.15 to –4.71) |
| Algeria | 165.88 (70.5–263.23) | 44.2 (22.98–78.06) | 1.55 (0.64–2.66) | 0.33 (0.17–0.65) | –4.77 (–4.93 to –4.6) |
| American Samoa | 0.04 (0.03–0.07) | 0.02 (0.01–0.03) | 0.23 (0.12–0.4) | 0.14 (0.08–0.27) | –1.66 (–1.95 to –1.36) |
| Andorra | 0 (0–0) | 0 (0–0) | 0 (0–0) | 0 (0–0) | –4.73 (–4.95 to –4.51) |
| Angola | 168.95 (95.09–258.69) | 134.42 (70.11–318.78) | 3.13 (1.66–4.99) | 0.85 (0.41–2.06) | –4.19 (–4.52 to –3.86) |
| Antigua and Barbuda | 0.15 (0.12–0.19) | 0.02 (0.02–0.02) | 0.87 (0.68–1.09) | 0.13 (0.11–0.16) | –5.87 (–6.74 to –4.98) |
| Argentina | 77.27 (71.25–84.27) | 24.93 (21.33–28.76) | 0.78 (0.69–0.87) | 0.26 (0.21–0.3) | –2.71 (–3.07 to –2.34) |
| Armenia | 4.13 (3.51–4.81) | 0.59 (0.46–0.75) | 0.38 (0.32–0.45) | 0.1 (0.08–0.13) | –7.58 (–9.27 to –5.85) |
| Australia | 3.63 (3.29–4) | 1.25 (1.06–1.43) | 0.1 (0.08–0.11) | 0.03 (0.02–0.03) | –4.09 (–4.59 to –3.59) |
| Austria | 2.16 (1.99–2.35) | 0.25 (0.21–0.28) | 0.16 (0.14–0.19) | 0.02 (0.02–0.02) | –5.96 (–6.6 to –5.31) |
| Azerbaijan | 16.11 (12.22–21.25) | 6.12 (4.38–8.56) | 0.64 (0.46–0.87) | 0.27 (0.18–0.42) | –3.89 (–4.57 to –3.2) |
| Bahamas | 0.54 (0.44–0.66) | 0.04 (0.03–0.05) | 0.71 (0.56–0.88) | 0.05 (0.04–0.08) | –8.24 (–9.44 to –7.02) |
| Bahrain | 0.42 (0.31–0.52) | 0.2 (0.15–0.27) | 0.25 (0.18–0.35) | 0.07 (0.05–0.1) | –3.9 (–4.11 to –3.69) |
| Bangladesh | 709.4 (32.36–1238.6) | 149.02 (9.04–288.64) | 1.39 (0.06–2.74) | 0.33 (0.02–0.73) | –3.98 (–4.29 to –3.66) |
| Barbados | 0.3 (0.25–0.35) | 0.02 (0.01–0.03) | 0.5 (0.4–0.61) | 0.05 (0.03–0.07) | –7.18 (–8.5 to –5.83) |
| Belarus | 38.84 (33.67–44.23) | 4.13 (3.16–5.26) | 1.65 (1.38–1.93) | 0.28 (0.2–0.38) | –5.95 (–6.79 to –5.1) |
| Belgium | 4.89 (4.49–5.32) | 0.66 (0.57–0.77) | 0.27 (0.24–0.31) | 0.03 (0.03–0.04) | –6.54 (–6.95 to –6.13) |
| Belize | 3.35 (2.91–3.86) | 0.19 (0.15–0.23) | 4 (3.39–4.7) | 0.16 (0.13–0.2) | –9.77 (–10.73 to –8.81) |
| Benin | 44.97 (24.89–69.1) | 73.89 (29.52–142.87) | 1.66 (0.87–2.75) | 1.14 (0.45–2.33) | –1.33 (–1.67 to –0.99) |
| Bermuda | 0.08 (0.07–0.1) | 0 (0–0) | 0.7 (0.55–0.88) | 0.04 (0.03–0.05) | –9.19 (–10.51 to –7.85) |
| Bhutan | 0.42 (0.22–0.99) | 0.12 (0.04–0.42) | 0.16 (0.07–0.38) | 0.06 (0.02–0.24) | –3.46 (–3.86 to –3.07) |
| Bolivia (Plurinational State of) | 51.34 (32.32–73.54) | 13.05 (8.54–21.22) | 1.84 (1.04–2.86) | 0.38 (0.21–0.65) | –5.1 (–5.18 to –5.01) |
| Bosnia and Herzegovina | 27.84 (11.1–39.79) | 1.82 (1.29–2.6) | 2.57 (1–4.18) | 0.38 (0.22–0.6) | –5.9 (–6.37 to –5.43) |
| Botswana | 7.24 (4.82–11.39) | 6.79 (3.94–11.77) | 1.22 (0.65–2.14) | 0.99 (0.48–1.81) | –0.09 (–0.39 to 0.2) |
| Brazil | 94.84 (83.77–108.8) | 12.23 (9.78–14.66) | 0.19 (0.17–0.22) | 0.03 (0.02–0.03) | –5.93 (–6.21 to –5.64) |
| Brunei Darussalam | 0.09 (0.06–0.12) | 0.03 (0.02–0.04) | 0.1 (0.06–0.16) | 0.03 (0.02–0.05) | –3.39 (–3.69 to –3.1) |
| Bulgaria | 17.52 (15.76–18.92) | 0.77 (0.65–0.92) | 1.06 (0.93–1.2) | 0.08 (0.06–0.1) | –8.46 (–8.85 to –8.08) |
| Burkina Faso | 181.85 (109.32–294.06) | 219.64 (110.96–517.49) | 3.47 (1.94–5.78) | 1.92 (0.91–4.65) | –1.7 (–1.88 to –1.52) |
| Burundi | 86.42 (49.7–128.79) | 73.68 (28.24–173.08) | 2.93 (1.59–4.66) | 1.22 (0.45–3.02) | –2.02 (–2.47 to –1.57) |
| Cabo Verde | 0.04 (0.01–0.16) | 0.18 (0.03–0.3) | 0.02 (0.01–0.1) | 0.13 (0.02–0.26) | 3.23 (0.8 to 5.72) |
| Cambodia | 15.84 (6.72–26.67) | 5.5 (3.43–9.04) | 0.32 (0.12–0.58) | 0.11 (0.06–0.19) | –3.83 (–4 to –3.66) |
| Cameroon | 60.48 (36.37–92.99) | 106.09 (44.02–188.71) | 1.11 (0.61–1.87) | 0.77 (0.32–1.46) | –1.15 (–1.58 to –0.72) |
| Canada | 9.73 (9.03–10.44) | 3.61 (3.18–4.05) | 0.17 (0.15–0.19) | 0.06 (0.05–0.07) | –2.34 (–2.73 to –1.94) |
| Central African Republic | 45.18 (26.72–69.93) | 45.11 (24.54–96.72) | 3.26 (1.77–5.37) | 1.91 (1–4.32) | –1.61 (–1.7 to –1.51) |
| Chad | 54.37 (25.86–91.81) | 150.82 (73.91–243.16) | 1.62 (0.72–2.86) | 1.53 (0.72–2.64) | –0.3 (–0.62 to 0.04) |
| Chile | 19.16 (17.96–20.49) | 2.75 (2.4–3.12) | 0.48 (0.43–0.53) | 0.08 (0.06–0.09) | –5.01 (–5.45 to –4.56) |
| China | 5225.83 (4016.23–8972.59) | 1088.03 (545.68–1461.57) | 1.64 (1.21–2.84) | 0.43 (0.21–0.59) | –3.98 (–4.65 to –3.31) |
| Colombia | 39.62 (34.52–45.31) | 5.82 (4.64–7.27) | 0.34 (0.28–0.4) | 0.05 (0.04–0.07) | –4.56 (–5.18 to –3.93) |
| Comoros | 5.39 (3.07–8.61) | 2.67 (1.49–4.95) | 2.34 (1.25–3.91) | 1.14 (0.59–2.25) | –2.24 (–2.44 to –2.04) |
| Congo | 17.11 (11.01–28.74) | 9.44 (4.57–24.99) | 1.54 (0.93–2.69) | 0.51 (0.23–1.44) | –3.71 (–4.01 to –3.42) |
| Cook Islands | 0.01 (0.01–0.02) | 0 (0–0) | 0.17 (0.08–0.3) | 0.03 (0.01–0.08) | –7.09 (–7.84 to –6.32) |
| Costa Rica | 1.67 (1.52–1.81) | 0.52 (0.45–0.6) | 0.15 (0.13–0.17) | 0.05 (0.04–0.06) | –3.06 (–3.37 to –2.75) |
| Coted'Ivoire | 63.72 (35.81–104.65) | 96.73 (38.66–176.04) | 1.02 (0.55–1.74) | 0.8 (0.32–1.56) | –0.71 (–1.08 to –0.33) |
| Croatia | 3.41 (3.16–3.66) | 0.17 (0.14–0.2) | 0.36 (0.32–0.41) | 0.03 (0.02–0.04) | –7.8 (–8.12 to –7.47) |
| Cuba | 9.05 (8.12–10.24) | 0.59 (0.48–0.72) | 0.36 (0.31–0.42) | 0.04 (0.03–0.04) | –7.46 (–8.49 to –6.42) |
| Cyprus | 0.23 (0.16–0.32) | 0.06 (0.04–0.08) | 0.12 (0.07–0.19) | 0.03 (0.01–0.04) | –4.47 (–5.05 to –3.89) |
| Czechia | 17.05 (15.78–18.33) | 0.78 (0.64–0.92) | 0.82 (0.73–0.91) | 0.05 (0.04–0.06) | –8.94 (–9.28 to –8.59) |
| Democratic People's Republic of Korea | 45.11 (25.58–94.36) | 19.73 (10.02–41.64) | 0.73 (0.36–1.55) | 0.42 (0.19–0.91) | –1.27 (–1.64 to –0.9) |
| Democratic Republic of the Congo | 470.68 (274.83–715.41) | 319.9 (155.87–780.31) | 2.34 (1.27–3.79) | 0.83 (0.37–2.18) | –2.85 (–3.19 to –2.52) |
| Denmark | 0.53 (0.49–0.58) | 0.06 (0.05–0.07) | 0.06 (0.05–0.07) | 0.01 (0–0.01) | –7.2 (–7.81 to –6.6) |
| Djibouti | 3.23 (1.94–5.36) | 3.57 (1.89–7.04) | 1.77 (1.03–3.07) | 0.86 (0.43–1.82) | –2.04 (–2.5 to –1.58) |
| Dominica | 0.6 (0.29–0.85) | 0.09 (0.05–0.2) | 2.42 (1.14–3.64) | 0.81 (0.41–1.8) | –3.5 (–4.48 to –2.52) |
| Dominican Republic | 35.85 (16.51–51.17) | 7.38 (3.63–14.8) | 1.28 (0.6–1.88) | 0.25 (0.12–0.54) | –4.93 (–5.7 to –4.16) |
| Ecuador | 56.09 (49.23–62.98) | 10.75 (8.58–13.43) | 1.45 (1.23–1.68) | 0.22 (0.17–0.28) | –6.21 (–6.74 to –5.67) |
| Egypt | 208.69 (124.28–285.06) | 61.17 (42.03–88.38) | 0.91 (0.49–1.34) | 0.17 (0.1–0.26) | –4.96 (–5.14 to –4.79) |
| El Salvador | 29.95 (22.39–37.71) | 5 (3.58–7.54) | 1.38 (0.97–1.86) | 0.28 (0.18–0.44) | –4.45 (–4.82 to –4.08) |
| Equatorial Guinea | 5.3 (3.26–8.19) | 2.32 (0.83–5.6) | 2.37 (1.37–3.88) | 0.42 (0.14–1.13) | –6.42 (–6.76 to –6.07) |
| Eritrea | 43.45 (26.29–66.78) | 39.71 (19.81–80.59) | 2.52 (1.4–4.08) | 1.53 (0.73–3.32) | –1.41 (–1.61 to –1.21) |
| Estonia | 3.01 (2.74–3.31) | 0.14 (0.12–0.18) | 0.87 (0.76–0.99) | 0.07 (0.05–0.09) | –8.13 (–8.55 to –7.71) |
| Eswatini | 6.11 (3.45–10.22) | 4.58 (2.92–7.2) | 1.55 (0.75–2.78) | 1.12 (0.61–1.9) | –0.65 (–0.83 to –0.47) |
| Ethiopia | 1047.54 (622.81–1582.77) | 556.28 (344.6–909.5) | 3.91 (2.24–6.07) | 1.23 (0.75–2.03) | –3.82 (–4.02 to –3.61) |
| Fiji | 0.85 (0.55–1.32) | 0.52 (0.33–0.78) | 0.3 (0.17–0.56) | 0.19 (0.1–0.33) | –2.19 (–2.68 to –1.7) |
| Finland | 3.34 (2.97–3.71) | 0.51 (0.43–0.6) | 0.35 (0.29–0.41) | 0.06 (0.05–0.07) | –5.71 (–6.07 to –5.35) |
| France | 14.4 (13.38–15.43) | 2.11 (1.88–2.35) | 0.12 (0.11–0.14) | 0.02 (0.02–0.02) | –5.8 (–6.43 to –5.16) |
| Gabon | 4.46 (2.59–7.59) | 2.61 (1.06–6.5) | 1.03 (0.55–1.88) | 0.42 (0.16–1.13) | –2.26 (–2.57 to –1.95) |
| Gambia | 6.59 (3.88–10.56) | 7.04 (3.67–15.03) | 1.3 (0.7–2.26) | 0.7 (0.32–1.68) | –2.31 (–2.67 to –1.95) |
| Georgia | 4.9 (4.22–5.63) | 0.61 (0.49–0.77) | 0.36 (0.3–0.43) | 0.09 (0.07–0.11) | –7.94 (–9.66 to –6.18) |
| Germany | 15.71 (13.78–17.69) | 2.58 (2.27–2.91) | 0.12 (0.1–0.14) | 0.02 (0.02–0.03) | –5.36 (–5.83 to –4.89) |
| Ghana | 110.42 (65.96–171.01) | 88.48 (38.62–193.9) | 1.52 (0.88–2.52) | 0.68 (0.28–1.55) | –2.17 (–2.37 to –1.97) |
| Greece | 1.97 (1.78–2.18) | 0.22 (0.19–0.26) | 0.1 (0.09–0.11) | 0.02 (0.01–0.02) | –4.87 (–5.54 to –4.19) |
| Greenland | 0.12 (0.06–0.15) | 0.03 (0.02–0.04) | 0.79 (0.42–1.14) | 0.23 (0.14–0.35) | –4.45 (–4.86 to –4.04) |
| Grenada | 0.56 (0.45–0.7) | 0.01 (0.01–0.01) | 1.65 (1.29–2.11) | 0.05 (0.04–0.07) | –9.43 (–10.42 to –8.43) |
| Guam | 0.08 (0.04–0.11) | 0.03 (0.02–0.04) | 0.19 (0.1–0.3) | 0.07 (0.04–0.12) | –4.25 (–4.98 to –3.52) |
| Guatemala | 91.83 (82.53–102.24) | 14.53 (11.53–18.01) | 2.18 (1.9–2.5) | 0.3 (0.23–0.39) | –5.27 (–6.01 to –4.52) |
| Guinea | 63.39 (33.79–101.06) | 73.73 (31.57–126.78) | 2.01 (0.98–3.45) | 1.17 (0.49–2.15) | –1.49 (–1.9 to –1.09) |
| Guinea–Bissau | 10.23 (5.6–16.91) | 8.12 (3.71–16.55) | 1.98 (1–3.43) | 0.88 (0.38–1.92) | –2.53 (–3.1 to –1.96) |
| Guyana | 4.04 (3.29–4.87) | 0.26 (0.2–0.33) | 1.31 (1.04–1.63) | 0.12 (0.09–0.16) | –6.27 (–7.19 to –5.34) |
| Haiti | 111.7 (34.39–208.01) | 41.36 (17.26–84.52) | 3.78 (1.11–7.21) | 0.93 (0.36–1.9) | –4.32 (–4.75 to –3.88) |
| Honduras | 28.23 (19.18–39.44) | 8.64 (4.1–20.2) | 1.25 (0.77–1.98) | 0.27 (0.11–0.67) | –4.96 (–5.02 to –4.89) |
| Hungary | 10.41 (9.44–11.38) | 0.47 (0.39–0.55) | 0.52 (0.46–0.59) | 0.03 (0.03–0.04) | –7.96 (–8.28 to –7.64) |
| Iceland | 0.15 (0.13–0.16) | 0.02 (0.02–0.03) | 0.23 (0.19–0.27) | 0.03 (0.02–0.04) | –6.14 (–6.83 to –5.45) |
| India | 764.19 (247.54–1052.21) | 256.41 (74.85–447.67) | 0.23 (0.07–0.34) | 0.07 (0.02–0.13) | –3.62 (–3.89 to –3.35) |
| Indonesia | 130.49 (33.54–185.89) | 73.25 (17.37–106.01) | 0.2 (0.05–0.3) | 0.11 (0.03–0.17) | –1.1 (–1.41 to –0.8) |
| Iran (Islamic Republic of) | 673.32 (399.6–894.11) | 42.11 (32.17–71.43) | 2.66 (1.58–3.64) | 0.2 (0.15–0.35) | –7.57 (–8.41 to –6.72) |
| Iraq | 42.1 (28.88–59.21) | 18.07 (12.05–29.03) | 0.5 (0.3–0.77) | 0.14 (0.08–0.24) | –3.92 (–4.27 to –3.57) |
| Ireland | 1.44 (1.33–1.57) | 0.12 (0.1–0.14) | 0.15 (0.13–0.17) | 0.01 (0.01–0.01) | –7.37 (–7.61 to –7.14) |
| Israel | 1.4 (1.3–1.5) | 0.24 (0.21–0.28) | 0.09 (0.08–0.1) | 0.01 (0.01–0.01) | –6.52 (–6.97 to –6.06) |
| Italy | 13.31 (12.8–13.83) | 1.13 (1.01–1.27) | 0.15 (0.14–0.15) | 0.01 (0.01–0.02) | –7.84 (–8.58 to –7.09) |
| Jamaica | 2.24 (1.89–2.62) | 0.26 (0.21–0.34) | 0.28 (0.23–0.33) | 0.05 (0.04–0.07) | –5.13 (–5.46 to –4.8) |
| Japan | 14.5 (14.09–14.93) | 2.09 (1.93–2.24) | 0.06 (0.06–0.07) | 0.01 (0.01–0.01) | –4.06 (–4.68 to –3.44) |
| Jordan | 21.15 (15.34–26.83) | 12.39 (9.42–16.64) | 1.28 (0.86–1.77) | 0.36 (0.24–0.5) | –4.53 (–4.87 to –4.19) |
| Kazakhstan | 112.41 (98.03–128.18) | 30.29 (25.22–36.76) | 2.12 (1.82–2.45) | 0.55 (0.45–0.68) | –4.84 (–5.82 to –3.84) |
| Kenya | 238.41 (135.73–384.78) | 159.38 (64.95–315.06) | 1.98 (1.1–3.25) | 0.91 (0.34–1.87) | –1.68 (–2.02 to –1.34) |
| Kiribati | 0.47 (0.26–0.68) | 0.39 (0.19–0.71) | 1.58 (0.75–2.68) | 0.92 (0.39–1.95) | –1.55 (–1.68 to –1.41) |
| Kuwait | 4.53 (3.95–5.27) | 1.88 (1.55–2.23) | 0.81 (0.68–0.97) | 0.23 (0.18–0.28) | –3.72 (–4.53 to –2.89) |
| Kyrgyzstan | 15.63 (13.4–18.1) | 5.32 (4.68–6.07) | 0.88 (0.75–1.04) | 0.23 (0.2–0.27) | –7.99 (–9.71 to –6.25) |
| Lao People's Democratic Republic | 7.07 (2.43–13.14) | 2.6 (1.54–4.23) | 0.37 (0.11–0.72) | 0.11 (0.06–0.2) | –3.88 (–4.03 to –3.74) |
| Latvia | 5.92 (5.38–6.49) | 0.23 (0.19–0.27) | 1.03 (0.9–1.17) | 0.08 (0.06–0.1) | –8.08 (–8.66 to –7.5) |
| Lebanon | 2.37 (1.56–3.45) | 0.76 (0.52–1.1) | 0.22 (0.13–0.37) | 0.06 (0.04–0.1) | –4.36 (–4.64 to –4.08) |
| Lesotho | 8.95 (5.81–14.12) | 8.41 (5.72–12.77) | 1.3 (0.71–2.15) | 1.34 (0.77–2.21) | 0.83 (0.55 to 1.12) |
| Liberia | 24.42 (12.22–39.26) | 18.66 (7.14–41.52) | 1.92 (0.86–3.24) | 0.85 (0.32–2.03) | –3.19 (–3.56 to –2.82) |
| Libya | 15.36 (6.69–23.1) | 6.36 (3.18–10.07) | 0.85 (0.36–1.48) | 0.46 (0.21–0.82) | –1.64 (–1.98 to –1.3) |
| Lithuania | 11.07 (9.88–12.32) | 0.62 (0.5–0.75) | 1.34 (1.17–1.52) | 0.15 (0.12–0.19) | –7.22 (–7.84 to –6.59) |
| Luxembourg | 0.1 (0.09–0.12) | 0.01 (0.01–0.02) | 0.16 (0.13–0.18) | 0.01 (0.01–0.02) | –7.81 (–8.68 to –6.94) |
| Madagascar | 141.04 (82.54–205.58) | 129.76 (69.36–215.62) | 2.35 (1.27–3.62) | 1.11 (0.56–1.91) | –1.92 (–2.11 to –1.74) |
| Malawi | 180.41 (107.23–265.59) | 77.12 (37.3–167.97) | 3.43 (1.96–5.24) | 0.98 (0.45–2.25) | –3.8 (–4 to –3.6) |
| Malaysia | 13.33 (9.06–18.89) | 4.47 (3.19–6.74) | 0.2 (0.12–0.32) | 0.06 (0.03–0.1) | –3.67 (–3.97 to –3.36) |
| Maldives | 0.16 (0.08–0.27) | 0.03 (0.02–0.05) | 0.14 (0.06–0.26) | 0.03 (0.01–0.06) | –4.84 (–5.06 to –4.61) |
| Mali | 95.64 (45.3–163.47) | 140.32 (64.76–241.39) | 2.03 (0.89–3.66) | 1.13 (0.49–2.02) | –1.96 (–2.32 to –1.6) |
| Malta | 0.07 (0.05–0.08) | 0.02 (0.01–0.02) | 0.08 (0.06–0.1) | 0.03 (0.02–0.03) | –4.75 (–5.65 to –3.84) |
| Marshall Islands | 0.08 (0.04–0.15) | 0.05 (0.03–0.1) | 0.37 (0.15–0.81) | 0.3 (0.12–0.61) | –1.05 (–1.54 to –0.56) |
| Mauritania | 7.97 (4.82–13.03) | 8.27 (3.89–17.39) | 0.79 (0.44–1.38) | 0.44 (0.2–0.96) | –2.25 (–2.8 to –1.69) |
| Mauritius | 0.06 (0.06–0.07) | 0.06 (0.05–0.07) | 0.02 (0.02–0.02) | 0.03 (0.03–0.04) | 0.16 (–1.6 to 1.94) |
| Mexico | 333.98 (303.48–374.36) | 44.59 (36.22–54.54) | 1 (0.9–1.12) | 0.14 (0.11–0.18) | –5.58 (–5.84 to –5.31) |
| Micronesia (Federated States of) | 0.3 (0.18–0.48) | 0.09 (0.05–0.17) | 0.65 (0.32–1.21) | 0.27 (0.13–0.58) | –2.93 (–3.01 to –2.85) |
| Monaco | 0.01 (0.01–0.02) | 0 (0–0.01) | 0.3 (0.13–0.64) | 0.1 (0.04–0.28) | –5.05 (–5.95 to –4.14) |
| Mongolia | 56.92 (27.68–92.17) | 8.19 (5.13–13.53) | 5.99 (2.83–10.11) | 0.75 (0.44–1.29) | –7.25 (–7.71 to –6.79) |
| Montenegro | 0.51 (0.37–0.67) | 0.08 (0.05–0.13) | 0.33 (0.21–0.46) | 0.07 (0.04–0.12) | –4.84 (–5.32 to –4.37) |
| Morocco | 208.06 (80–348.3) | 41.88 (17.95–92.66) | 2.09 (0.75–4.01) | 0.43 (0.18–0.98) | –4.77 (–5.03 to –4.51) |
| Mozambique | 258.47 (153.07–383.44) | 187.66 (89.05–437.29) | 3.82 (2.11–5.87) | 1.28 (0.59–3.1) | –3.21 (–3.37 to –3.05) |
| Myanmar | 50.63 (21.28–160.08) | 17.78 (9.03–44.1) | 0.34 (0.11–1.13) | 0.11 (0.05–0.31) | –3.92 (–4.15 to –3.69) |
| Namibia | 6.67 (4.17–10.25) | 6.88 (4.5–10.89) | 1.09 (0.57–1.82) | 0.84 (0.47–1.45) | –0.36 (–0.66 to –0.06) |
| Nauru | 0.02 (0.01–0.03) | 0.01 (0.01–0.02) | 0.51 (0.24–0.94) | 0.37 (0.19–0.69) | –1.07 (–1.72 to –0.4) |
| Nepal | 654.4 (340.87–1051.9) | 181.73 (115.91–290.32) | 7.44 (3.31–12.39) | 1.96 (1.05–3.54) | –4 (–4.17 to –3.82) |
| Netherlands | 1.65 (1.5–1.8) | 0.2 (0.17–0.23) | 0.06 (0.05–0.07) | 0.01 (0.01–0.01) | –6.44 (–6.93 to –5.93) |
| New Zealand | 0.7 (0.63–0.77) | 0.3 (0.27–0.34) | 0.09 (0.07–0.1) | 0.03 (0.02–0.04) | –2.65 (–3.68 to –1.61) |
| Nicaragua | 12.49 (7.17–17.45) | 1.65 (1.05–2.73) | 0.67 (0.37–0.99) | 0.08 (0.05–0.15) | –5.93 (–6.53 to –5.31) |
| Niger | 117.8 (50.26–204.21) | 176.4 (71.09–378.98) | 2.56 (0.97–4.73) | 1.27 (0.5–2.8) | –2.61 (–2.98 to –2.23) |
| Nigeria | 789.6 (384.01–1605.03) | 1034.62 (706.38–1527.98) | 1.81 (0.88–3.68) | 0.99 (0.66–1.5) | –1.72 (–1.87 to –1.57) |
| Niue | 0 (0–0.01) | 0 (0–0.01) | 0.44 (0.22–0.84) | 0.87 (0.42–1.68) | –0.63 (–1.6 to 0.34) |
| North Macedonia | 2.68 (1.69–3.49) | 0.18 (0.13–0.28) | 0.53 (0.33–0.74) | 0.05 (0.03–0.09) | –6.49 (–6.99 to –5.98) |
| Northern Mariana Islands | 0.02 (0.01–0.03) | 0.02 (0.01–0.03) | 0.16 (0.08–0.29) | 0.16 (0.07–0.28) | –0.1 (–0.57 to 0.37) |
| Norway | 0.69 (0.65–0.74) | 0.32 (0.29–0.36) | 0.09 (0.08–0.09) | 0.03 (0.03–0.04) | –1.53 (–3.09 to 0.06) |
| Oman | 0.86 (0.53–1.27) | 0.24 (0.18–0.32) | 0.1 (0.05–0.17) | 0.02 (0.01–0.03) | –4.16 (–4.56 to –3.75) |
| Pakistan | 83.45 (43.05–241.09) | 78.87 (39.35–253.9) | 0.16 (0.08–0.47) | 0.09 (0.04–0.3) | –1.2 (–1.46 to –0.95) |
| Palau | 0.06 (0.03–0.08) | 0.02 (0.01–0.03) | 1.21 (0.59–2.14) | 0.57 (0.3–1.06) | –1.6 (–2.03 to –1.17) |
| Palestine | 3.61 (2.27–5.15) | 1.84 (1.22–2.82) | 0.35 (0.2–0.58) | 0.1 (0.06–0.17) | –3.42 (–3.73 to –3.12) |
| Panama | 2.97 (2.52–3.46) | 0.91 (0.73–1.11) | 0.36 (0.29–0.44) | 0.08 (0.06–0.1) | –4.22 (–4.66 to –3.76) |
| Papua New Guinea | 16.98 (8.16–27.1) | 25.07 (14.16–42.43) | 1.01 (0.39–1.96) | 0.65 (0.29–1.27) | –1.3 (–1.48 to –1.13) |
| Paraguay | 7.19 (4.65–9.21) | 2.36 (1.58–3.75) | 0.42 (0.27–0.57) | 0.12 (0.07–0.2) | –3.58 (–3.82 to –3.34) |
| Peru | 92.42 (57.55–123.27) | 16.28 (10.74–25.13) | 1.11 (0.67–1.59) | 0.17 (0.1–0.29) | –6.04 (–6.4 to –5.67) |
| Philippines | 28.95 (16.9–39.83) | 15.43 (10.4–19.82) | 0.11 (0.06–0.16) | 0.05 (0.03–0.06) | –2.23 (–2.41 to –2.05) |
| Poland | 81.39 (78.79–83.92) | 3.15 (2.84–3.44) | 0.87 (0.83–0.91) | 0.05 (0.05–0.06) | –8.47 (–8.91 to –8.03) |
| Portugal | 16.74 (15.44–18.14) | 0.94 (0.81–1.07) | 0.83 (0.72–0.94) | 0.07 (0.06–0.08) | –8.23 (–8.91 to –7.54) |
| Puerto Rico | 7.89 (7.1–8.64) | 0.03 (0.02–0.03) | 0.83 (0.72–0.96) | 0.01 (0.01–0.01) | –12.79 (–13.92 to –11.63) |
| Qatar | 0.72 (0.51–1.01) | 0.61 (0.41–0.95) | 0.56 (0.34–0.89) | 0.12 (0.07–0.21) | –4.33 (–4.73 to –3.92) |
| Republic of Korea | 81.58 (36.76–109.91) | 2.24 (1.58–4) | 0.7 (0.3–1.01) | 0.04 (0.02–0.06) | –9.19 (–9.77 to –8.61) |
| Republic of Moldova | 40.48 (35.35–46.85) | 3.29 (2.63–4.09) | 3.28 (2.78–3.89) | 0.67 (0.52–0.87) | –5.42 (–5.77 to –5.06) |
| Romania | 157.42 (136.15–176.82) | 7.58 (6.51–8.86) | 3 (2.56–3.46) | 0.26 (0.21–0.32) | –7.9 (–8.42 to –7.38) |
| Russian Federation | 795.38 (767.95–822.93) | 167.32 (155.36–176.49) | 2.34 (2.24–2.43) | 0.66 (0.6–0.72) | –4.06 (–4.53 to –3.59) |
| Rwanda | 97.74 (60.77–151.42) | 42.49 (21.17–97.79) | 2.63 (1.58–4.28) | 0.85 (0.4–2.08) | –4.06 (–4.47 to –3.65) |
| Saint Kitts and Nevis | 0.1 (0.08–0.11) | 0.01 (0.01–0.01) | 0.71 (0.59–0.84) | 0.07 (0.05–0.1) | –6.86 (–7.94 to –5.76) |
| Saint Lucia | 0.48 (0.37–0.58) | 0.03 (0.02–0.03) | 0.94 (0.72–1.17) | 0.09 (0.07–0.13) | –7.37 (–8.66 to –6.07) |
| Saint Vincent and the Grenadines | 0.28 (0.22–0.34) | 0.01 (0.01–0.02) | 0.73 (0.56–0.92) | 0.05 (0.04–0.07) | –7.74 (–8.58 to –6.89) |
| Samoa | 0.33 (0.2–0.54) | 0.16 (0.09–0.29) | 0.46 (0.23–0.85) | 0.2 (0.09–0.41) | –2.75 (–2.87 to –2.63) |
| San Marino | 0.01 (0.01–0.01) | 0 (0–0) | 0.26 (0.15–0.42) | 0.05 (0.03–0.09) | –4.81 (–5.14 to –4.48) |
| Sao Tome and Principe | 0.88 (0.49–1.52) | 0.19 (0.07–0.58) | 1.53 (0.74–3.03) | 0.24 (0.08–0.81) | –5.55 (–5.97 to –5.13) |
| Saudi Arabia | 49.97 (31.54–73.95) | 7.79 (4.61–13.16) | 0.75 (0.41–1.22) | 0.1 (0.05–0.19) | –6.11 (–6.3 to –5.93) |
| Senegal | 57.81 (32.78–91.56) | 42.63 (18.99–97.39) | 1.44 (0.76–2.43) | 0.66 (0.28–1.58) | –2.47 (–2.95 to –1.99) |
| Serbia | 8.93 (5.39–11.67) | 0.53 (0.37–0.91) | 0.43 (0.24–0.62) | 0.04 (0.03–0.07) | –8.17 (–8.8 to –7.53) |
| Seychelles | 0.02 (0.02–0.03) | 0.01 (0.01–0.02) | 0.1 (0.07–0.16) | 0.05 (0.03–0.07) | –1.86 (–2.17 to –1.55) |
| Sierra Leone | 42.92 (21.07–68.1) | 46.46 (20.48–81.82) | 2.04 (0.92–3.39) | 1.24 (0.54–2.27) | –1.94 (–2.33 to –1.54) |
| Singapore | 0.35 (0.31–0.38) | 0.02 (0.02–0.03) | 0.05 (0.05–0.06) | 0 (0–0) | –7.83 (–8.57 to –7.08) |
| Slovakia | 4.1 (2.76–5.19) | 0.65 (0.48–1) | 0.32 (0.21–0.44) | 0.08 (0.05–0.12) | –4.71 (–4.89 to –4.53) |
| Slovenia | 1.05 (0.94–1.15) | 0.03 (0.03–0.04) | 0.27 (0.23–0.31) | 0.01 (0.01–0.01) | –9.24 (–9.65 to –8.83) |
| Solomon Islands | 1.18 (0.62–2.39) | 1.12 (0.55–2.54) | 0.77 (0.34–1.73) | 0.43 (0.18–1.08) | –1.81 (–1.99 to –1.64) |
| Somalia | 136.7 (75.92–215.18) | 224.76 (106.64–466.79) | 3.17 (1.68–5.23) | 1.99 (0.88–4.3) | –1.02 (–1.41 to –0.64) |
| South Africa | 204.57 (134.23–267.46) | 119.14 (86.45–148.72) | 1.49 (0.87–2.11) | 0.79 (0.56–1.03) | –1.93 (–2.14 to –1.73) |
| South Sudan | 88.98 (44.14–148.89) | 120.81 (64.95–200.38) | 3.1 (1.46–5.35) | 2.73 (1.41–4.73) | –0.11 (–0.66 to 0.45) |
| Spain | 13.67 (12.54–14.87) | 1.34 (1.2–1.5) | 0.18 (0.15–0.2) | 0.02 (0.02–0.02) | –6.77 (–7.25 to –6.3) |
| Sri Lanka | 7.25 (4.78–9.24) | 1.23 (0.85–1.83) | 0.13 (0.08–0.19) | 0.02 (0.02–0.04) | –5.84 (–6.35 to –5.33) |
| Sudan | 396.65 (145.37–688.07) | 156.87 (74.93–260.35) | 4.15 (1.33–7.96) | 0.96 (0.43–1.72) | –4.55 (–4.77 to –4.32) |
| Suriname | 1.41 (0.51–2.08) | 0.24 (0.14–0.5) | 1.11 (0.39–1.71) | 0.18 (0.09–0.38) | –5.67 (–6.43 to –4.9) |
| Sweden | 0.94 (0.87–1.01) | 0.3 (0.27–0.34) | 0.06 (0.05–0.07) | 0.02 (0.01–0.02) | –2.96 (–3.98 to –1.93) |
| Switzerland | 0.33 (0.31–0.36) | 0.05 (0.04–0.05) | 0.03 (0.03–0.03) | 0 (0–0) | –6.77 (–7.34 to –6.2) |
| Syrian Arab Republic | 36.55 (23.97–50) | 7.78 (5.29–10.76) | 0.61 (0.35–0.9) | 0.22 (0.14–0.33) | –2.39 (–3 to –1.77) |
| Taiwan (Province of China) | 34.98 (32.04–37.85) | 3.51 (3.06–4) | 0.64 (0.56–0.73) | 0.12 (0.1–0.14) | –3.54 (–4.4 to –2.67) |
| Tajikistan | 29.32 (19.95–40.96) | 22.4 (11.52–51.66) | 1.13 (0.73–1.63) | 0.6 (0.3–1.38) | –3.54 (–4.13 to –2.94) |
| Thailand | 5.34 (2.75–15.64) | 3.53 (2.42–4.9) | 0.03 (0.01–0.1) | 0.04 (0.02–0.06) | 2.2 (1.63 to 2.78) |
| Timor–Leste | 1.17 (0.4–2.17) | 0.64 (0.41–1.05) | 0.32 (0.1–0.62) | 0.12 (0.07–0.23) | –3.25 (–3.5 to –3) |
| Togo | 21.83 (13.63–32.56) | 23.24 (9.54–49.6) | 1.15 (0.63–1.88) | 0.7 (0.28–1.55) | –1.73 (–2.09 to –1.37) |
| Tokelau | 0 (0–0.01) | 0 (0–0.01) | 0.47 (0.22–0.98) | 1.06 (0.49–2.59) | –1.6 (–3.14 to –0.04) |
| Tonga | 0.51 (0.33–0.76) | 0.29 (0.19–0.46) | 1.2 (0.69–2) | 0.74 (0.42–1.3) | –1.57 (–1.85 to –1.3) |
| Trinidad and Tobago | 2.83 (2.41–3.26) | 0.15 (0.12–0.2) | 0.71 (0.58–0.85) | 0.06 (0.04–0.08) | –8.24 (–9.11 to –7.37) |
| Tunisia | 43.42 (18.16–67.84) | 6.34 (3.03–12.72) | 1.41 (0.58–2.48) | 0.23 (0.11–0.5) | –5.56 (–5.63 to –5.48) |
| Turkey | 158.15 (99.91–299.95) | 38.59 (27.99–55.58) | 0.78 (0.44–1.52) | 0.22 (0.14–0.34) | –3.07 (–3.54 to –2.6) |
| Turkmenistan | 10.14 (8.71–11.8) | 1.95 (1.62–2.35) | 0.62 (0.53–0.73) | 0.13 (0.1–0.16) | –8.69 (–10.29 to –7.06) |
| Tuvalu | 0.03 (0.02–0.05) | 0.01 (0.01–0.02) | 0.94 (0.4–1.77) | 0.29 (0.13–0.62) | –3.58 (–3.68 to –3.48) |
| Uganda | 205.46 (121.95–306.69) | 221.57 (100.38–458.8) | 2.08 (1.16–3.25) | 1.08 (0.48–2.28) | –1.84 (–2.05 to –1.63) |
| Ukraine | 126.83 (109.81–146.11) | 17.62 (15.06–20.64) | 1.14 (0.96–1.37) | 0.31 (0.24–0.39) | –4.74 (–5.19 to –4.3) |
| United Arab Emirates | 2.15 (1.35–3.1) | 0.82 (0.58–1.15) | 0.36 (0.21–0.6) | 0.06 (0.04–0.1) | –4.87 (–5.17 to –4.57) |
| United Kingdom | 10.79 (10.48–11.08) | 1.8 (1.69–1.9) | 0.1 (0.09–0.1) | 0.02 (0.01–0.02) | –7.15 (–7.71 to –6.59) |
| United Republic of Tanzania | 394.06 (237.16–589.42) | 289.22 (138.99–640.16) | 2.94 (1.72–4.54) | 1.15 (0.52–2.62) | –2.52 (–2.71 to –2.33) |
| United States of America | 86.58 (84.66–88.58) | 48.33 (43.88–52.7) | 0.15 (0.15–0.16) | 0.08 (0.08–0.09) | –1.05 (–1.42 to –0.68) |
| United States Virgin Islands | 0.12 (0.06–0.18) | 0.01 (0–0.02) | 0.37 (0.19–0.63) | 0.07 (0.03–0.14) | –4.95 (–5.51 to –4.39) |
| Uruguay | 5.53 (5.09–6) | 1.1 (0.93–1.32) | 0.69 (0.62–0.78) | 0.17 (0.14–0.22) | –4.22 (–4.68 to –3.75) |
| Uzbekistan | 60.98 (53.21–70.05) | 31.26 (26.04–37.72) | 0.66 (0.56–0.76) | 0.3 (0.25–0.37) | –4.9 (–6.01 to –3.78) |
| Vanuatu | 0.37 (0.21–0.73) | 0.4 (0.2–0.78) | 0.57 (0.27–1.19) | 0.35 (0.16–0.71) | –1.56 (–1.89 to –1.22) |
| Venezuela (Bolivarian Republic of) | 22.62 (20.31–25.02) | 10.41 (7.67–13.42) | 0.32 (0.27–0.37) | 0.16 (0.11–0.21) | –2.19 (–2.8 to –1.58) |
| Viet Nam | 26.12 (14.11–60.06) | 7.58 (3.99–18.4) | 0.1 (0.04–0.26) | 0.03 (0.01–0.08) | –3.48 (–3.68 to –3.28) |
| Yemen | 217.45 (76.08–381.2) | 120.44 (55.23–224.46) | 2.87 (0.96–5.66) | 0.88 (0.39–1.79) | –4.06 (–4.21 to –3.91) |
| Zambia | 122.2 (74.9–183.94) | 74.83 (41.48–146.07) | 2.9 (1.71–4.5) | 0.9 (0.47–1.86) | –3.53 (–3.84 to –3.22) |
| Zimbabwe | 105.8 (41.58–168.43) | 144.59 (75.83–222.91) | 2.14 (0.81–3.63) | 2.29 (1.09–3.95) | 1.12 (0.51 to 1.74) |

**Supplementary Table 6.** Disability–Adjusted Life Years (DALYs) and Age–Standardized DALYs Rates(ASDR) of Childhood Poisoning in 204 Countries and Territories, 1990–2021

| location | Dalys_1990 | Dalys_2021 | ASDR_1990 | ASDR_2021 | EAPC |
| --- | --- | --- | --- | --- | --- |
| Afghanistan | 18172.97 (7151.65–32320.91) | 19761.84 (9630.7–36977.61) | 391.26 (146.7–771.48) | 133.63 (60.76–262.47) | –3.35 (–3.65 to –3.05) |
| Albania | 906.11 (537.08–1249.44) | 105.87 (73.88–153.38) | 80.07 (45.1–118.12) | 24.24 (14.66–37.95) | –3.97 (–4.14 to –3.79) |
| Algeria | 14391.18 (6200.84–22865.83) | 3845.09 (2035.86–6660.99) | 134.39 (56.82–229.87) | 28.87 (14.85–55.37) | –4.76 (–4.92 to –4.59) |
| American Samoa | 3.82 (2.43–5.75) | 1.88 (1.25–3) | 20.19 (11.47–35) | 12.71 (7.27–22.72) | –1.62 (–1.88 to –1.37) |
| Andorra | 0.98 (0.63–1.43) | 0.87 (0.52–1.27) | 9.86 (5.71–15.17) | 7.91 (4.41–12.21) | –0.62 (–0.67 to –0.56) |
| Angola | 14860.39 (8364.07–22699.55) | 11846.63 (6191.69–28090.02) | 275 (147.2–436.89) | 74.72 (36.61–181.24) | –4.19 (–4.52 to –3.85) |
| Antigua and Barbuda | 14.41 (11.77–17.33) | 2.52 (2.1–3.02) | 81.45 (64.44–101.67) | 15.61 (12.06–19.86) | –5.19 (–5.96 to –4.41) |
| Argentina | 9360.08 (8215.03–10656.44) | 4158.19 (3374.86–5206.89) | 93.28 (79.31–109.63) | 40.93 (31.5–53.05) | –1.98 (–2.29 to –1.68) |
| Armenia | 400.13 (345.87–459.92) | 72.37 (59.14–89.73) | 37.42 (31.38–43.77) | 12.48 (9.42–16.54) | –6.56 (–8.02 to –5.07) |
| Australia | 638.57 (502.55–810) | 475.57 (331.7–656.62) | 16.81 (12.88–21.87) | 9.78 (6.5–14.46) | –1.7 (–1.88 to –1.51) |
| Austria | 347.38 (278.19–434.84) | 153.18 (99.79–220.68) | 25.82 (20.3–33.23) | 11.69 (7.4–17.62) | –2.03 (–2.47 to –1.59) |
| Azerbaijan | 1513.25 (1171.13–1965.58) | 610.59 (459.02–817.68) | 60.19 (44.34–80.64) | 27.02 (18.52–39.62) | –3.65 (–4.27 to –3.02) |
| Bahamas | 50.96 (42–61.22) | 6.72 (5.03–8.83) | 66.87 (53.45–82.33) | 8.51 (5.76–12.29) | –6.39 (–7.42 to –5.36) |
| Bahrain | 39.27 (30.02–48.23) | 21.04 (16.52–27.04) | 23.54 (16.94–32.02) | 7.16 (5.17–9.78) | –3.61 (–3.79 to –3.43) |
| Bangladesh | 61123.79 (3180.04–106907.93) | 12595.58 (1019.52–24693.68) | 119.22 (6.19–234.72) | 27.77 (2.03–61.65) | –4.04 (–4.34 to –3.74) |
| Barbados | 28.66 (24.23–33.67) | 3.93 (2.99–5.22) | 48.02 (38.54–58.36) | 8.43 (5.69–12.11) | –5.27 (–6.24 to –4.29) |
| Belarus | 3467.62 (3003.83–3931.31) | 412.05 (328.57–520.27) | 146.96 (124.08–171.69) | 27.2 (19.94–36.77) | –5.69 (–6.5 to –4.88) |
| Belgium | 714.34 (590.99–874.2) | 244.78 (172.06–333.26) | 39.48 (31.97–49.07) | 12.52 (8.29–18.05) | –3.27 (–3.56 to –2.98) |
| Belize | 298.78 (260.9–343.19) | 20.76 (17.33–24.92) | 356.56 (303.55–417.88) | 17.79 (13.78–22.53) | –9.22 (–10.17 to –8.26) |
| Benin | 3968.58 (2238.2–6088.85) | 6509.64 (2657.58–12511.16) | 146.73 (78.12–240.53) | 100.4 (40.4–203.71) | –1.34 (–1.68 to –0.99) |
| Bermuda | 8.27 (6.74–10.02) | 0.63 (0.46–0.83) | 68.09 (53.63–85.64) | 7.59 (5.06–10.77) | –7.08 (–8.26 to –5.87) |
| Bhutan | 39.25 (20.84–89.07) | 10.87 (4.3–36.72) | 14.64 (6.77–34.25) | 5.89 (2.03–20.75) | –3.42 (–3.79 to –3.04) |
| Bolivia (Plurinational State of) | 4588.21 (2957.9–6499.07) | 1211.86 (818.41–1907.28) | 164.17 (95.65–252.52) | 35.06 (20.8–58.72) | –4.98 (–5.06 to –4.9) |
| Bosnia and Herzegovina | 2558.22 (1185.51–3543.32) | 210.75 (157.44–278.68) | 235.73 (106.59–372.2) | 43 (28.25–63.97) | –5.33 (–5.77 to –4.89) |
| Botswana | 622.14 (415.09–962.91) | 590.69 (345.2–1018.84) | 104.5 (56.39–181.57) | 85.86 (42.96–157.13) | –0.04 (–0.33 to 0.26) |
| Brazil | 9621.15 (8667.46–10860.95) | 1771.67 (1460.46–2142.61) | 19.4 (16.91–22.5) | 3.68 (2.9–4.64) | –5.18 (–5.37 to –4.98) |
| Brunei Darussalam | 16.74 (12.54–21.61) | 9.73 (7.02–13.66) | 18.84 (13.02–26) | 10.16 (6.65–14.88) | –1.82 (–1.92 to –1.72) |
| Bulgaria | 1767.54 (1596.3–1947.21) | 163.35 (124.67–213.47) | 105.81 (91.92–120.6) | 16.42 (11.98–22.3) | –6.38 (–6.59 to –6.16) |
| Burkina Faso | 16026.68 (9645.2–25942.96) | 19399.5 (9851.59–45625.79) | 305.27 (172–508.94) | 169.63 (81.43–408.54) | –1.69 (–1.87 to –1.51) |
| Burundi | 7607.97 (4394.38–11364.4) | 6474.62 (2523.83–15219.58) | 257.71 (141.89–405.96) | 106.9 (40.33–264.13) | –2.02 (–2.48 to –1.57) |
| Cabo Verde | 6.58 (3.68–17.49) | 17.1 (4.71–27.43) | 4.18 (2.03–11.36) | 12.12 (3.06–23.35) | 1.88 (–0.01 to 3.8) |
| Cambodia | 1411.97 (619.32–2330.62) | 498.38 (325.54–801.06) | 28.73 (11.38–51.03) | 9.81 (5.64–16.99) | –3.77 (–3.93 to –3.62) |
| Cameroon | 5334.07 (3237.84–8184.71) | 9337.26 (4023.23–16567.37) | 98.16 (55.04–163.27) | 68.03 (28.95–126.68) | –1.16 (–1.59 to –0.73) |
| Canada | 1276.56 (1098.28–1505.61) | 701.1 (550.73–907.6) | 22.26 (18.27–27.25) | 11.26 (8.21–15.26) | –1.58 (–1.8 to –1.36) |
| Central African Republic | 3970.42 (2344.02–6149.75) | 3948.95 (2154.59–8446.99) | 286.15 (156–468.48) | 167.1 (88.52–375.61) | –1.62 (–1.71 to –1.52) |
| Chad | 4783.31 (2293.1–8075.67) | 13186.17 (6535.85–21204.78) | 142.85 (64.57–249.22) | 133.82 (64.21–228.96) | –0.32 (–0.65 to 0.01) |
| Chile | 2225.75 (1978.96–2525.17) | 878.63 (633.39–1203.97) | 55.57 (47.53–66.01) | 23.53 (16.48–32.98) | –2.2 (–2.55 to –1.86) |
| China | 452808.96 (348778.09–777949.96) | 92477.92 (47867.68–124075.07) | 142.07 (105.01–244.53) | 36.44 (18.2–50.46) | –4.05 (–4.72 to –3.38) |
| Colombia | 4748.17 (4105.91–5452.81) | 1260.18 (961.35–1651.36) | 40.55 (33.57–48.4) | 11.68 (8.41–16.03) | –3.36 (–3.75 to –2.96) |
| Comoros | 476.87 (272.01–762.7) | 234.71 (131.41–436.96) | 207.16 (111.6–344.82) | 100.01 (52.15–196.49) | –2.25 (–2.45 to –2.05) |
| Congo | 1510.96 (974.62–2524.63) | 830.17 (406.25–2184.24) | 136.22 (82.33–236.04) | 44.74 (20.31–125.22) | –3.73 (–4.03 to –3.43) |
| Cook Islands | 1.03 (0.63–1.48) | 0.15 (0.09–0.29) | 15.39 (8.24–26.3) | 3.72 (2.02–8.12) | –5.76 (–6.32 to –5.2) |
| Costa Rica | 257.43 (215.38–315.3) | 128.49 (95.95–170.92) | 22.91 (18.59–29.22) | 12.3 (8.76–16.77) | –1.82 (–1.94 to –1.71) |
| Coted'Ivoire | 5642.84 (3177.56–9202.58) | 8512.83 (3518.62–15462.88) | 90.09 (49.55–152.13) | 70.61 (29.19–135.6) | –0.73 (–1.1 to –0.36) |
| Croatia | 401.67 (350.54–460.99) | 75.48 (53.19–106.31) | 41.38 (34.91–49.33) | 12.16 (8.2–17.6) | –3.68 (–3.95 to –3.4) |
| Cuba | 905.87 (812.34–1029.46) | 159.69 (118.16–213.96) | 35.85 (30.63–42.44) | 8.9 (6.07–12.73) | –4.51 (–5.27 to –3.75) |
| Cyprus | 40.84 (30.27–52.36) | 21.52 (15.03–29.81) | 20.64 (13.94–28.6) | 9.79 (6.21–14.29) | –2.21 (–2.37 to –2.05) |
| Czechia | 1840.84 (1651.16–2044.64) | 289.52 (202.13–398.47) | 86.37 (75.53–98.64) | 16.47 (11.05–23.27) | –5.1 (–5.43 to –4.77) |
| Democratic People's Republic of Korea | 3919.3 (2241.45–8113.32) | 1679.88 (868.48–3510.76) | 62.96 (32.27–132.11) | 35.92 (16.54–77.27) | –1.31 (–1.68 to –0.95) |
| Democratic Republic of the Congo | 41520.74 (24292.06–63173.22) | 27996.96 (13762.88–68028.08) | 205.93 (112.32–332.48) | 72.78 (32.82–189.95) | –2.88 (–3.22 to –2.55) |
| Denmark | 127.12 (94.31–165.1) | 84.67 (52.67–122.49) | 14.24 (10.16–19.58) | 8.69 (4.79–13.79) | –1.4 (–1.58 to –1.22) |
| Djibouti | 286.14 (174.14–471.41) | 315.64 (168.58–617.81) | 157.15 (92.7–270.62) | 76.03 (38.33–158.79) | –2.06 (–2.51 to –1.6) |
| Dominica | 53.98 (27.29–76.17) | 8.77 (5.46–18.38) | 218.32 (108.51–323.77) | 75.38 (41.14–160.85) | –3.39 (–4.33 to –2.45) |
| Dominican Republic | 3284.13 (1645.95–4596.75) | 744.57 (425.45–1399.51) | 117.28 (57.93–170.3) | 25.31 (13.34–50.54) | –4.57 (–5.29 to –3.85) |
| Ecuador | 4968.42 (4379.87–5558.31) | 1068.46 (877.31–1312.96) | 128.86 (109.49–148.49) | 21.41 (16.6–27.63) | –5.84 (–6.34 to –5.34) |
| Egypt | 18031.1 (10799.2–24635.01) | 5458.98 (3896.35–7803.79) | 78.42 (42.37–114.77) | 14.82 (9.45–22.5) | –4.86 (–5.03 to –4.69) |
| El Salvador | 2763.28 (2124.1–3442.1) | 558.83 (428.94–768.78) | 127.24 (92.61–168.84) | 30.65 (21.3–45.5) | –3.93 (–4.25 to –3.61) |
| Equatorial Guinea | 465.61 (286.22–720.13) | 205.4 (78.28–490.38) | 207.57 (120.91–338.14) | 37.05 (13.04–98.92) | –6.38 (–6.71 to –6.04) |
| Eritrea | 3817.64 (2327.12–5851.93) | 3483.47 (1747.45–7040.58) | 220.84 (124.46–354.9) | 134.32 (64.59–289.02) | –1.41 (–1.61 to –1.22) |
| Estonia | 276.52 (251.95–302.95) | 24.74 (19.29–31.8) | 79.67 (69.67–90.88) | 11.32 (8.28–15.52) | –6.53 (–6.79 to –6.27) |
| Eswatini | 527.78 (301.26–888.29) | 392.68 (254.16–610.33) | 133.41 (65.78–237.54) | 95.79 (52.91–161.9) | –0.66 (–0.85 to –0.48) |
| Ethiopia | 92435.73 (55424.56–139307.72) | 48886.39 (30384.19–79861.71) | 344.87 (200.07–531.42) | 108.07 (65.8–178.27) | –3.83 (–4.03 to –3.63) |
| Fiji | 73.15 (48.44–112.58) | 45.28 (29.67–65.74) | 25.81 (14.77–46.86) | 16.48 (9.06–28.02) | –2.13 (–2.6 to –1.66) |
| Finland | 361.36 (318.46–412.9) | 105.94 (80.1–138.85) | 37.39 (31.44–44.63) | 12.09 (8.64–16.64) | –3.42 (–3.64 to –3.21) |
| France | 2384.77 (1899.98–2975.65) | 1174.3 (772.32–1669.47) | 20.35 (15.72–26.34) | 9.86 (5.92–14.82) | –1.94 (–2.25 to –1.64) |
| Gabon | 396.41 (231.22–675.55) | 230.39 (97.73–569.6) | 91.19 (49.68–165.06) | 37.02 (14.27–98.26) | –2.28 (–2.59 to –1.96) |
| Gambia | 581.01 (346.7–926.14) | 617.19 (322.74–1315.14) | 114.48 (63.61–196.7) | 61.15 (29.25–145.47) | –2.33 (–2.68 to –1.97) |
| Georgia | 500.9 (439.54–571.53) | 99.69 (77.01–123.76) | 36.9 (30.5–44.61) | 13.66 (9.89–18.36) | –5.94 (–7.37 to –4.49) |
| Germany | 2480.27 (1994.03–3100.09) | 1179.05 (784.85–1711.27) | 19.15 (14.66–24.88) | 9.79 (6.22–14.65) | –1.95 (–2.18 to –1.73) |
| Ghana | 9712.69 (5870.21–15010.18) | 7801.29 (3486.52–16923.79) | 133.69 (78.51–219.26) | 59.54 (24.94–134.63) | –2.16 (–2.36 to –1.96) |
| Greece | 354.91 (277.7–451.49) | 127.71 (84.8–185.91) | 17.26 (13.05–22.92) | 8.87 (5.46–13.61) | –1.79 (–2.03 to –1.55) |
| Greenland | 11.06 (6.86–14.12) | 2.86 (2.2–3.79) | 76.07 (44.99–106.08) | 24.4 (16.52–35.41) | –4.05 (–4.4 to –3.69) |
| Grenada | 51.36 (41.43–63.53) | 1.85 (1.46–2.36) | 151.2 (119.39–191.34) | 8.48 (6.14–11.57) | –8.07 (–8.95 to –7.18) |
| Guam | 6.97 (4.25–9.43) | 2.47 (1.63–3.74) | 17.22 (10.12–26.04) | 6.54 (3.85–10.92) | –3.77 (–4.36 to –3.18) |
| Guatemala | 8384.69 (7552.94–9328) | 1650.35 (1376.39–1999.99) | 199.47 (174.33–227.47) | 33.93 (26.76–42.99) | –4.76 (–5.42 to –4.09) |
| Guinea | 5587.67 (3002.09–8868.51) | 6489.59 (2820.5–11098.41) | 176.85 (88.29–300.91) | 103.25 (43.93–186.95) | –1.5 (–1.9 to –1.1) |
| Guinea–Bissau | 896.42 (493.29–1471.29) | 707.04 (326.96–1422.59) | 173.18 (89.1–297.4) | 76.64 (33.38–165.62) | –2.55 (–3.12 to –1.98) |
| Guyana | 363.81 (298.07–436.73) | 31.3 (25.05–38.45) | 118.38 (94.64–145.12) | 14.68 (10.84–19.25) | –5.53 (–6.37 to –4.67) |
| Haiti | 9951.24 (3194.46–18328.1) | 3818.13 (1722.45–7611.61) | 337.11 (104.67–634.76) | 86.01 (35.92–171.52) | –4.21 (–4.63 to –3.78) |
| Honduras | 2727.47 (1967.91–3691.54) | 1040.36 (625.14–2015.41) | 121.28 (80.19–183.09) | 31.84 (17.71–65.79) | –4.29 (–4.33 to –4.25) |
| Hungary | 1192.91 (1063.36–1348.65) | 187.86 (133.08–267.95) | 58 (49.89–67.75) | 13.23 (9.04–19.42) | –4.49 (–4.74 to –4.24) |
| Iceland | 18.62 (16–22.08) | 7.6 (5.28–10.38) | 29.3 (23.9–36.24) | 11 (7.15–15.91) | –2.74 (–3.16 to –2.32) |
| India | 67851 (24705.61–92013.2) | 23223.83 (8465.73–39642.98) | 20.81 (7.49–29.41) | 6.42 (2.27–11.58) | –3.58 (–3.83 to –3.32) |
| Indonesia | 11827.04 (3680.08–16548.03) | 6660.01 (2033.71–9478.24) | 17.79 (5.36–26.47) | 10.05 (2.97–15.21) | –1.14 (–1.41 to –0.86) |
| Iran (Islamic Republic of) | 58624.44 (34931.24–77868.67) | 3562.81 (2710.34–5968.16) | 231.58 (138.16–315.52) | 17.37 (12.53–29.41) | –7.64 (–8.49 to –6.78) |
| Iraq | 3665.25 (2538.22–5119.59) | 1653.04 (1133.22–2591.89) | 43.51 (26.67–66.17) | 12.61 (7.77–21.31) | –3.79 (–4.12 to –3.45) |
| Ireland | 218.8 (177.66–269.68) | 96.92 (64.23–139.02) | 22.21 (17.62–28.63) | 9.41 (5.72–14.27) | –2.54 (–2.76 to –2.31) |
| Israel | 281.7 (218.55–367.3) | 222.83 (143.84–322.88) | 18.37 (13.8–24.5) | 8.48 (4.93–13.14) | –2.1 (–2.4 to –1.8) |
| Italy | 2481.89 (1971.77–3090.47) | 739.59 (505.67–1010.97) | 26.42 (21.13–33.18) | 9.3 (6.41–12.98) | –3.73 (–4.27 to –3.19) |
| Jamaica | 243.62 (211.43–284.13) | 52.08 (40.46–68.44) | 29.82 (24.39–36.64) | 8.97 (6.38–12.26) | –3.52 (–3.78 to –3.27) |
| Japan | 3653.88 (2794.5–4774.71) | 1320.14 (904.31–1843.76) | 15.45 (11.85–20.19) | 8.2 (5.63–11.58) | –1.76 (–2 to –1.51) |
| Jordan | 1827 (1334.82–2313.93) | 1076.34 (827.38–1427.14) | 110.02 (74.9–151.1) | 30.92 (21.46–43.12) | –4.49 (–4.82 to –4.16) |
| Kazakhstan | 10163.16 (8933.81–11499.06) | 2825.49 (2388.5–3400.99) | 191.65 (165.17–221.61) | 51.59 (42.01–62.96) | –4.76 (–5.72 to –3.8) |
| Kenya | 21086.54 (12046.86–33914.65) | 14012.74 (5763.58–27640.55) | 175.28 (98.31–285.25) | 79.9 (30.75–163.84) | –1.72 (–2.05 to –1.38) |
| Kiribati | 40.27 (22.54–57.67) | 32.58 (16.34–59.32) | 134.03 (64.83–225.19) | 77.49 (33.35–162.45) | –1.58 (–1.71 to –1.45) |
| Kuwait | 391.5 (342.52–455.63) | 166.55 (138.92–197.68) | 69.58 (58.57–83.7) | 20.18 (16.21–24.73) | –3.64 (–4.44 to –2.84) |
| Kyrgyzstan | 1452.44 (1262.85–1663.9) | 538.25 (474.39–612.37) | 82.52 (69.85–97.16) | 23.7 (19.98–28.37) | –7.46 (–9.05 to –5.84) |
| Lao People's Democratic Republic | 633 (237.87–1161.44) | 234.27 (146.24–374.81) | 32.85 (11.14–63.24) | 10.13 (5.65–17.71) | –3.87 (–4 to –3.73) |
| Latvia | 534.02 (485.42–582.14) | 34.82 (27.02–43.49) | 93.11 (81.5–105.12) | 11.82 (8.64–15.87) | –6.9 (–7.32 to –6.48) |
| Lebanon | 217.98 (149.29–310.89) | 76.66 (55.64–103.77) | 20.49 (12.2–32.6) | 6.07 (3.91–9.32) | –4.08 (–4.34 to –3.83) |
| Lesotho | 770.31 (503.96–1212.5) | 713.66 (489.69–1077.39) | 111.89 (62.51–182.93) | 114.39 (65.94–186.03) | 0.79 (0.51 to 1.07) |
| Liberia | 2156.11 (1078.83–3457.5) | 1629.7 (641.17–3627.29) | 169.4 (77.88–283.56) | 74.5 (28.53–176.07) | –3.22 (–3.58 to –2.85) |
| Libya | 1345.12 (598.73–2008.91) | 549.89 (284.2–865.78) | 74.15 (32.31–127.68) | 40 (18.44–70.15) | –1.67 (–2 to –1.34) |
| Lithuania | 1001.12 (905.13–1104.12) | 68.45 (57.82–80.98) | 120.88 (106.03–137.52) | 16.88 (13.47–20.99) | –6.7 (–7.24 to –6.16) |
| Luxembourg | 15.43 (12.79–19.04) | 9.29 (6.09–12.92) | 23.35 (18.35–30.13) | 9.04 (5.49–13.37) | –2.74 (–3.2 to –2.29) |
| Madagascar | 12494.56 (7322.11–18220.39) | 11425.82 (6154.55–18913.72) | 207.95 (114.09–318.53) | 97.7 (50.23–167.62) | –1.95 (–2.13 to –1.76) |
| Malawi | 15956.3 (9498.57–23451.42) | 6798.01 (3338.32–14855.26) | 303.03 (174.5–460.49) | 86.19 (40.7–197.41) | –3.81 (–4.01 to –3.62) |
| Malaysia | 1213.1 (854.21–1682.49) | 446.92 (332.69–635) | 18.36 (11.25–28.75) | 5.88 (3.67–9.12) | –3.41 (–3.67 to –3.15) |
| Maldives | 14.75 (7.63–24.5) | 3.26 (2.19–5.07) | 13.34 (6.41–23.68) | 3.31 (1.86–5.93) | –4.19 (–4.38 to –4) |
| Mali | 8420.39 (4014.37–14285.69) | 12353.89 (5823.95–21033.53) | 178.89 (79.75–319.01) | 99.02 (44.82–176.1) | –1.97 (–2.32 to –1.61) |
| Malta | 14.66 (11.12–18.95) | 6.59 (4.66–9.04) | 16.71 (11.94–22.48) | 10.24 (6.71–15.01) | –1.33 (–1.58 to –1.07) |
| Marshall Islands | 6.94 (3.4–12.51) | 4.56 (2.28–8.01) | 31.65 (13.89–67.48) | 25.5 (11.11–50.73) | –1.07 (–1.55 to –0.59) |
| Mauritania | 706.48 (433.06–1146.77) | 730.89 (354.57–1524.85) | 70.28 (39.63–120.36) | 39.15 (18.22–83.64) | –2.25 (–2.8 to –1.69) |
| Mauritius | 10.17 (8.2–12.88) | 7.77 (6.51–9.35) | 3.07 (2.28–4.17) | 3.8 (2.88–4.96) | –0.21 (–1.5 to 1.1) |
| Mexico | 31897.69 (28986.2–35680.32) | 5766.44 (4755.66–6899.69) | 95.2 (85.37–108.13) | 18.13 (14.19–23.05) | –4.7 (–4.94 to –4.46) |
| Micronesia (Federated States of) | 25.03 (15.35–39.56) | 7.23 (4.15–13.78) | 54.32 (27.79–100.16) | 22.55 (11.12–47.68) | –2.9 (–2.98 to –2.82) |
| Monaco | 1.41 (0.96–2.14) | 0.82 (0.56–1.48) | 40.03 (24.04–69.66) | 16.53 (9.9–34.71) | –3.26 (–3.83 to –2.68) |
| Mongolia | 5042.76 (2505.88–8098.74) | 739.43 (467.77–1203) | 531.23 (258.49–885.67) | 67.64 (40.9–114.32) | –7.19 (–7.65 to –6.74) |
| Montenegro | 63.68 (48.33–78.77) | 17.69 (12.77–24.04) | 39.92 (28–53.55) | 15.5 (10.15–23.03) | –3.04 (–3.29 to –2.8) |
| Morocco | 18070.75 (7003.27–30109.15) | 3626.63 (1595.27–7976.37) | 180.95 (66.02–345.86) | 37.66 (16.08–84.06) | –4.78 (–5.04 to –4.52) |
| Mozambique | 22812.01 (13543.53–33796.22) | 16541.02 (7958.87–38547.71) | 336.66 (187.72–515.2) | 112.92 (53.01–272.22) | –3.22 (–3.37 to –3.06) |
| Myanmar | 4715.39 (2238.11–14249.98) | 1813.9 (1082.37–4089.7) | 32.07 (12.26–100.22) | 11.61 (5.94–28.29) | –3.62 (–3.83 to –3.41) |
| Namibia | 578.09 (368.91–889.93) | 592.03 (394.43–930.4) | 93.9 (51.07–156.25) | 72.52 (41.26–123.28) | –0.37 (–0.67 to –0.07) |
| Nauru | 1.79 (1.06–2.84) | 1.25 (0.75–2) | 43.41 (21.27–78.28) | 31.42 (16.01–57.27) | –1.09 (–1.74 to –0.44) |
| Nepal | 55914.19 (28887.48–90143.21) | 15138.61 (9616.05–24416.23) | 632.83 (282.64–1054.42) | 163.5 (87.35–296.44) | –4.06 (–4.23 to –3.89) |
| Netherlands | 403.23 (306.1–540.81) | 230.97 (147.31–335.53) | 14.74 (10.56–20.27) | 8.43 (4.94–13.06) | –1.48 (–1.7 to –1.25) |
| New Zealand | 156.14 (119.78–206.05) | 131.63 (91.36–181.97) | 19.3 (14.46–25.9) | 13.07 (8.75–18.49) | –1.22 (–1.59 to –0.84) |
| Nicaragua | 1309.86 (845.27–1743.79) | 289.88 (208.49–398.21) | 70.82 (42.68–98.21) | 14.6 (9.6–21.31) | –4.72 (–5.19 to –4.26) |
| Niger | 10336.97 (4402.46–17844.85) | 15469.98 (6356.75–33053.98) | 224.13 (86.46–412.23) | 111.32 (45.25–243.8) | –2.61 (–2.98 to –2.24) |
| Nigeria | 69487.31 (34104.71–140677.77) | 90879.11 (62322.83–133650.39) | 159.62 (78.59–322.27) | 86.89 (58.01–130.36) | –1.73 (–1.88 to –1.58) |
| Niue | 0.3 (0.19–0.53) | 0.29 (0.17–0.49) | 37.44 (19.5–70.09) | 72.5 (35.47–138.71) | –0.64 (–1.58 to 0.31) |
| North Macedonia | 297.44 (208.94–368.48) | 47.75 (34.24–64.85) | 58.07 (38.08–77.96) | 14.09 (9.47–20.44) | –4.19 (–4.51 to –3.87) |
| Northern Mariana Islands | 1.73 (1.06–2.65) | 1.73 (0.97–2.68) | 14.57 (8.24–25.14) | 13.63 (6.41–23.7) | –0.22 (–0.62 to 0.18) |
| Norway | 274.47 (199.65–368.39) | 366.91 (247.99–511.81) | 34.28 (24.62–46.06) | 38.65 (26.01–54.23) | 0.62 (0.32 to 0.92) |
| Oman | 87.9 (59.29–122.7) | 32.3 (24.46–41.04) | 10.24 (6.21–15.75) | 2.67 (1.76–3.92) | –3.59 (–3.9 to –3.28) |
| Pakistan | 7784.36 (4313.66–21480.16) | 7287.1 (3894.07–22084.13) | 15.36 (7.96–42.05) | 8.55 (4.04–26.31) | –1.28 (–1.52 to –1.05) |
| Palau | 4.71 (2.87–6.95) | 1.61 (1.08–2.59) | 102.29 (51.13–179.12) | 48.69 (26.06–88.99) | –1.61 (–2.02 to –1.2) |
| Palestine | 321.55 (207.97–457.15) | 174.73 (122.68–257.72) | 31.34 (18.34–50.64) | 9.5 (5.96–15.22) | –3.27 (–3.56 to –2.97) |
| Panama | 348.46 (299.71–405.9) | 171.09 (133.7–220.53) | 41.92 (34.11–51.06) | 14.82 (10.89–19.93) | –3.04 (–3.32 to –2.77) |
| Papua New Guinea | 1430.63 (693.68–2275.16) | 2119.95 (1208.55–3539.76) | 84.38 (33.5–162.75) | 54.42 (24.61–105.48) | –1.3 (–1.48 to –1.12) |
| Paraguay | 671.75 (455.47–851.26) | 240.43 (168.69–357.7) | 39.21 (26.09–52.44) | 12.21 (7.89–19.65) | –3.37 (–3.58 to –3.17) |
| Peru | 8454.14 (5483.09–11224.53) | 1632.08 (1123.35–2394.03) | 101.48 (63.66–143.53) | 17.14 (10.64–26.97) | –5.76 (–6.09 to –5.43) |
| Philippines | 2775.33 (1738.33–3700.91) | 1581.46 (1190.17–2005.74) | 10.83 (6.37–14.74) | 4.71 (3.34–6.07) | –2.03 (–2.22 to –1.83) |
| Poland | 8031.56 (7570.13–8589.97) | 623.81 (490.97–801.18) | 85.26 (79.32–92.14) | 10.22 (7.88–13.27) | –6.38 (–6.79 to –5.96) |
| Portugal | 1669.66 (1521.67–1836) | 197.53 (147.97–261.33) | 81.86 (70.71–93.32) | 14.24 (10.27–19.92) | –5.59 (–6.21 to –4.96) |
| Puerto Rico | 746.75 (673.05–825.16) | 25.95 (16.49–37.57) | 78.44 (68.01–89.69) | 5.35 (3.24–8.12) | –8.15 (–9.04 to –7.24) |
| Qatar | 63.28 (45.03–88.01) | 56.23 (39.24–85.79) | 49.05 (30.09–76.6) | 11.3 (6.89–18.85) | –4.19 (–4.57 to –3.82) |
| Republic of Korea | 8252.24 (4531.37–10547.1) | 643.83 (442.41–912.73) | 71.23 (38.5–96.79) | 10.11 (6.35–14.82) | –6.21 (–6.78 to –5.63) |
| Republic of Moldova | 3611.93 (3156.92–4139.91) | 307.95 (250.6–378.43) | 292.58 (248.73–346.68) | 62.33 (48.56–80.76) | –5.31 (–5.64 to –4.97) |
| Romania | 15160.15 (13232.36–17140.41) | 949.37 (802–1136.81) | 286.66 (244.11–331.12) | 31.79 (25.47–39.37) | –7.2 (–7.64 to –6.77) |
| Russian Federation | 70627.51 (68006.52–73433.87) | 14758.69 (13719.96–15713.81) | 207.77 (197.97–217.57) | 58.52 (52.49–63.55) | –4.1 (–4.56 to –3.64) |
| Rwanda | 8604.12 (5422.58–13328.8) | 3756.99 (1903.02–8640.8) | 230.99 (139.72–373.71) | 75.31 (35.61–182.4) | –4.04 (–4.45 to –3.62) |
| Saint Kitts and Nevis | 9.26 (8.07–10.64) | 1.01 (0.81–1.27) | 67.45 (56.05–79.87) | 10.58 (7.46–14.54) | –5.63 (–6.55 to –4.7) |
| Saint Lucia | 44.3 (35.55–52.72) | 3.53 (2.8–4.41) | 87.12 (67.93–107.93) | 12.07 (8.75–16.51) | –6.15 (–7.25 to –5.05) |
| Saint Vincent and the Grenadines | 26.18 (21.41–31.97) | 2.15 (1.71–2.78) | 67.97 (53.13–85.9) | 8.62 (6.29–11.8) | –6.02 (–6.74 to –5.3) |
| Samoa | 27.93 (17.35–44.89) | 13.43 (7.88–23.96) | 39.09 (20.72–70.66) | 17 (8.34–34.19) | –2.69 (–2.79 to –2.58) |
| San Marino | 1.44 (1.06–1.88) | 0.67 (0.47–0.93) | 35.68 (23.7–51.42) | 14.78 (9.48–21.98) | –2.75 (–3.04 to –2.47) |
| Sao Tome and Principe | 76.34 (43.79–132.92) | 16.64 (6.94–49.56) | 132.64 (65.76–260.21) | 21.88 (7.81–70.02) | –5.48 (–5.89 to –5.06) |
| Saudi Arabia | 4380.87 (2786.92–6495.31) | 713.13 (443.28–1150.78) | 65.39 (37.27–105.39) | 9.47 (5.3–16.81) | –5.99 (–6.16 to –5.83) |
| Senegal | 5102.58 (2920.75–8041.24) | 3741.97 (1701.11–8489.27) | 126.98 (68.75–211.6) | 58.21 (25.45–136.44) | –2.49 (–2.97 to –2.01) |
| Serbia | 1073.33 (753.82–1352.94) | 199.58 (134.81–278.35) | 50.66 (32.01–68.53) | 14.13 (9.21–21.05) | –4.4 (–4.68 to –4.12) |
| Seychelles | 2.48 (1.89–3.33) | 1.23 (0.95–1.63) | 10.52 (7.31–15.21) | 5.32 (3.71–7.67) | –1.76 (–2.01 to –1.51) |
| Sierra Leone | 3789.11 (1878.11–5998.32) | 4093.36 (1835.11–7159.24) | 179.65 (82.89–296.96) | 109.23 (48.73–197.91) | –1.95 (–2.33 to –1.55) |
| Singapore | 95.39 (70.98–128.99) | 69.93 (43.78–105.8) | 14.41 (10.11–20.41) | 8.7 (5–13.83) | –1.34 (–1.52 to –1.16) |
| Slovakia | 522.66 (402.86–627.14) | 141.46 (103.84–189.18) | 39.86 (28.07–51.39) | 16.36 (11.19–23.76) | –2.94 (–3.04 to –2.85) |
| Slovenia | 144.06 (121.55–172.79) | 42.1 (27.15–62.85) | 35.19 (28.8–43.35) | 12.96 (8.04–19.64) | –2.81 (–3.07 to –2.55) |
| Solomon Islands | 98.97 (52.99–199.77) | 93.99 (47.74–209) | 64.73 (29.48–142.08) | 36.62 (15.65–89.42) | –1.79 (–1.97 to –1.62) |
| Somalia | 12048.85 (6762.67–18996.68) | 19758.22 (9469.72–41075.12) | 279.61 (149.76–458.43) | 174.38 (78.29–374.37) | –1.03 (–1.41 to –0.65) |
| South Africa | 17735.66 (11658.23–23247.14) | 10185.34 (7431.26–12631.93) | 129.1 (76.42–181.82) | 68.05 (48.36–88.26) | –1.93 (–2.14 to –1.72) |
| South Sudan | 7880.86 (3937.35–13174.76) | 10602.49 (5730.45–17623.82) | 274.91 (130.83–471.09) | 239.6 (125.16–413.09) | –0.13 (–0.68 to 0.41) |
| Spain | 2000.7 (1657.96–2404.68) | 669.05 (452.99–953.58) | 25.24 (20.39–31.52) | 9.91 (6.26–14.88) | –2.79 (–3.11 to –2.46) |
| Sri Lanka | 690.69 (488.29–862.62) | 150.17 (110.47–208.27) | 12.68 (8.37–17.33) | 2.94 (1.96–4.43) | –5.06 (–5.48 to –4.63) |
| Sudan | 34540.79 (12683.15–60006.56) | 13519.47 (6527.2–22464.59) | 361.02 (116.5–686.42) | 82.5 (37.32–147.6) | –4.57 (–4.8 to –4.35) |
| Suriname | 130.27 (51.64–189.08) | 26.54 (16.86–50.13) | 102.3 (39.9–154.2) | 19.45 (10.93–37.55) | –5.16 (–5.84 to –4.46) |
| Sweden | 327.94 (238.53–436.51) | 229.33 (155.34–328.42) | 21.27 (14.94–28.99) | 12.31 (8.04–18.47) | –1.65 (–1.99 to –1.32) |
| Switzerland | 123.32 (89.87–168) | 108.84 (69.19–156.97) | 10.64 (7.05–15.28) | 8.06 (4.66–12.41) | –0.29 (–0.6 to 0.03) |
| Syrian Arab Republic | 3190.16 (2099.57–4357.76) | 679.72 (470.23–930.53) | 53.16 (31.04–77.67) | 19.2 (12.33–28.38) | –2.38 (–2.97 to –1.78) |
| Taiwan (Province of China) | 2943.49 (2708.27–3181.02) | 320.76 (279.99–366.38) | 53.9 (47.52–61.15) | 10.95 (8.92–13.06) | –3.36 (–4.18 to –2.52) |
| Tajikistan | 2659.1 (1824.83–3685.24) | 2069.03 (1126.83–4567.36) | 102.94 (67.81–146.99) | 55.7 (29.51–122.13) | –3.41 (–3.96 to –2.85) |
| Thailand | 631.89 (383.85–1501.69) | 366.55 (275.46–483.58) | 3.65 (1.92–9.06) | 3.82 (2.46–5.81) | 1.39 (0.96 to 1.83) |
| Timor–Leste | 104.51 (37.97–192.03) | 57.91 (38.37–92.79) | 28.41 (9.99–54.61) | 11.07 (6.26–20.3) | –3.22 (–3.46 to –2.99) |
| Togo | 1925.08 (1194.76–2862.02) | 2037.16 (858.53–4314.57) | 101.8 (56.11–164.49) | 61.14 (24.9–134.43) | –1.76 (–2.11 to –1.4) |
| Tokelau | 0.24 (0.14–0.46) | 0.35 (0.19–0.78) | 40.34 (20.07–80.67) | 88.64 (40.95–214.37) | –1.56 (–3.06 to –0.03) |
| Tonga | 43.19 (28.78–64.34) | 24.71 (16.08–38.86) | 102.42 (59.04–169.6) | 62.99 (36.23–108.67) | –1.59 (–1.87 to –1.32) |
| Trinidad and Tobago | 263.69 (228.17–303.19) | 24.08 (18.86–30.93) | 66.24 (54.87–78.83) | 9.07 (6.46–12.6) | –6.68 (–7.43 to –5.92) |
| Tunisia | 3785.37 (1608.99–5915.94) | 558.43 (277.59–1099.53) | 122.98 (50.96–213.75) | 20.58 (10.02–43.1) | –5.53 (–5.6 to –5.46) |
| Turkey | 13866.07 (8902.05–26121.39) | 3408.59 (2504.57–4820.47) | 68.51 (39.75–131.85) | 19.24 (12.65–29.35) | –3.08 (–3.54 to –2.61) |
| Turkmenistan | 964.01 (831.1–1109.21) | 224.64 (187.36–267.27) | 60.02 (50.7–70.92) | 14.71 (11.55–18.8) | –7.86 (–9.35 to –6.35) |
| Tuvalu | 2.71 (1.52–4.19) | 0.91 (0.49–1.67) | 79.44 (34.25–147.07) | 24.25 (11.11–51.1) | –3.58 (–3.67 to –3.48) |
| Uganda | 18219.77 (10885.81–27186.34) | 19534.95 (8983.23–40537.6) | 184.13 (104.19–286.78) | 94.92 (43.15–199.84) | –1.85 (–2.06 to –1.65) |
| Ukraine | 11316.79 (9817.32–13011.07) | 1660.22 (1425.08–1928.66) | 102.02 (86.46–121.2) | 29.07 (22.6–35.8) | –4.59 (–5.02 to –4.17) |
| United Arab Emirates | 191.46 (124.52–272.51) | 85.86 (63.45–115.3) | 32.17 (18.96–51.95) | 6.51 (4.24–9.88) | –4.46 (–4.73 to –4.2) |
| United Kingdom | 2313.7 (1825.54–2921.49) | 1212.23 (844.94–1677.15) | 21.17 (16.59–27.12) | 10 (6.87–13.96) | –2.96 (–3.19 to –2.72) |
| United Republic of Tanzania | 34908.35 (21057.69–52251.67) | 25683.96 (12505.85–56289.44) | 260.04 (153.21–401.25) | 102.51 (47.2–229.22) | –2.52 (–2.71 to –2.33) |
| United States of America | 13245.99 (11109.05–16028.21) | 7977.31 (6613.68–9722.74) | 23.65 (19.68–28.81) | 13.53 (11–16.66) | –1.5 (–2.13 to –0.87) |
| United States Virgin Islands | 11.99 (7.43–17.53) | 1.42 (0.94–2.14) | 37.69 (21.61–59.95) | 11.11 (6.01–19.02) | –3.74 (–4.14 to –3.33) |
| Uruguay | 617.46 (547.33–698.91) | 189.78 (150.28–237.91) | 76.69 (66.38–89.18) | 28.87 (21.62–38.32) | –3.05 (–3.36 to –2.73) |
| Uzbekistan | 5714.36 (4994.78–6547.97) | 3028.03 (2558.3–3619.06) | 61.97 (52.93–72.24) | 29.3 (23.98–35.83) | –4.62 (–5.63 to –3.59) |
| Vanuatu | 31.31 (17.93–60.73) | 33.46 (17.69–64.76) | 47.61 (23.44–98.38) | 29.14 (14.03–58.79) | –1.55 (–1.88 to –1.21) |
| Venezuela (Bolivarian Republic of) | 2828.25 (2409.2–3323.19) | 1489.2 (1182.93–1864.53) | 39.74 (32.78–47.58) | 22.48 (16.94–29.54) | –1.78 (–2.11 to –1.46) |
| Viet Nam | 2542.79 (1478.86–5452.85) | 798.95 (469.34–1720.26) | 9.6 (4.73–23.13) | 3.21 (1.67–7.32) | –3.28 (–3.45 to –3.11) |
| Yemen | 18957.46 (6666.22–33236.15) | 10369.8 (4821.27–19138.59) | 249.93 (85.21–489.5) | 75.99 (34.29–152.52) | –4.09 (–4.24 to –3.93) |
| Zambia | 10825.77 (6658.31–16281.44) | 6607.88 (3723.3–12845.89) | 256.73 (152.89–398.07) | 79.31 (42.08–163.15) | –3.54 (–3.84 to –3.23) |
| Zimbabwe | 9275.66 (3686.81–14804.51) | 12474.09 (6549.75–19418.15) | 187.43 (72.23–316.96) | 197.91 (94.24–341.39) | 1.04 (0.45 to 1.63) |

**Supplementary Table 7.** Frontier of DALY–related Burden of Childhood Poisoning and Effective Difference by Country or Territory

| Location | SDI | Rate of DALYs | Frontier DALYs | Effective difference | Effective difference rank (Age–standardized DALYs rank) |
| --- | --- | --- | --- | --- | --- |
| Afghanistan | 0.3372 | 133.63 (60.76 to 262.47) | 5.09 | 128.54 | 198 (196) |
| Albania | 0.70685 | 24.24 (14.66 to 37.95) | 2.7 | 21.54 | 119 (116) |
| Algeria | 0.659501 | 28.87 (14.85 to 55.37) | 2.89 | 25.98 | 127 (125) |
| American Samoa | 0.723728 | 12.71 (7.27 to 22.72) | 2.66 | 10.06 | 79 (76) |
| Andorra | 0.869444 | 7.91 (4.41 to 12.21) | 2.64 | 5.27 | 24 (20) |
| Angola | 0.453722 | 74.72 (36.61 to 181.24) | 4.73 | 69.99 | 168 (167) |
| Antigua and Barbuda | 0.749887 | 15.61 (12.06 to 19.86) | 2.65 | 12.97 | 95 (93) |
| Argentina | 0.723123 | 40.93 (31.5 to 53.05) | 2.72 | 38.21 | 147 (145) |
| Armenia | 0.701833 | 12.48 (9.42 to 16.54) | 2.62 | 9.85 | 76 (73) |
| Australia | 0.844253 | 9.78 (6.5 to 14.46) | 2.64 | 7.14 | 47 (41) |
| Austria | 0.853837 | 11.69 (7.4 to 17.62) | 2.69 | 9 | 68 (64) |
| Azerbaijan | 0.694851 | 27.02 (18.52 to 39.62) | 2.93 | 24.08 | 124 (121) |
| Bahamas | 0.805021 | 8.51 (5.76 to 12.29) | 2.63 | 5.89 | 33 (27) |
| Bahrain | 0.753043 | 7.16 (5.17 to 9.78) | 3.07 | 4.09 | 21 (18) |
| Bangladesh | 0.492421 | 27.77 (2.03 to 61.65) | 4.9 | 22.87 | 123 (123) |
| Barbados | 0.746749 | 8.43 (5.69 to 12.11) | 2.67 | 5.76 | 29 (24) |
| Belarus | 0.784485 | 27.2 (19.94 to 36.77) | 2.61 | 24.59 | 126 (122) |
| Belgium | 0.853654 | 12.52 (8.29 to 18.05) | 2.62 | 9.9 | 78 (74) |
| Belize | 0.610229 | 17.79 (13.78 to 22.53) | 2.87 | 14.92 | 105 (103) |
| Benin | 0.373487 | 100.4 (40.4 to 203.71) | 4.72 | 95.68 | 189 (187) |
| Bermuda | 0.821365 | 7.59 (5.06 to 10.77) | 2.62 | 4.97 | 22 (19) |
| Bhutan | 0.473062 | 5.89 (2.03 to 20.75) | 4.6 | 1.29 | 11 (13) |
| Bolivia (Plurinational State of) | 0.599011 | 35.06 (20.8 to 58.72) | 2.87 | 32.19 | 137 (135) |
| Bosnia and Herzegovina | 0.723078 | 43 (28.25 to 63.97) | 2.75 | 40.25 | 148 (146) |
| Botswana | 0.642722 | 85.86 (42.96 to 157.13) | 2.93 | 82.92 | 182 (177) |
| Brazil | 0.653044 | 3.68 (2.9 to 4.64) | 2.97 | 0.71 | 7 (5) |
| Brunei Darussalam | 0.810234 | 10.16 (6.65 to 14.88) | 2.63 | 7.53 | 56 (51) |
| Bulgaria | 0.768151 | 16.42 (11.98 to 22.3) | 2.66 | 13.76 | 97 (95) |
| Burkina Faso | 0.285118 | 169.63 (81.43 to 408.54) | 4.66 | 164.97 | 202 (201) |
| Burundi | 0.289374 | 106.9 (40.33 to 264.13) | 4.46 | 102.44 | 193 (190) |
| Cabo Verde | 0.533535 | 12.12 (3.06 to 23.35) | 4.05 | 8.07 | 59 (68) |
| Cambodia | 0.473621 | 9.81 (5.64 to 16.99) | 4.41 | 5.4 | 25 (44) |
| Cameroon | 0.479691 | 68.03 (28.95 to 126.68) | 4.41 | 63.62 | 161 (160) |
| Canada | 0.873171 | 11.26 (8.21 to 15.26) | 2.7 | 8.56 | 63 (59) |
| Central African Republic | 0.309168 | 167.1 (88.52 to 375.61) | 4.49 | 162.61 | 201 (200) |
| Chad | 0.240436 | 133.82 (64.21 to 228.96) | 33.12 | 100.7 | 192 (197) |
| Chile | 0.771515 | 23.53 (16.48 to 32.98) | 2.86 | 20.67 | 116 (114) |
| China | 0.72163 | 36.44 (18.2 to 50.46) | 2.77 | 33.67 | 140 (137) |
| Colombia | 0.655443 | 11.68 (8.41 to 16.03) | 2.89 | 8.79 | 66 (63) |
| Comoros | 0.475979 | 100.01 (52.15 to 196.49) | 4.34 | 95.67 | 188 (186) |
| Congo | 0.583075 | 44.74 (20.31 to 125.22) | 2.93 | 41.81 | 149 (147) |
| Cook Islands | 0.77911 | 3.72 (2.02 to 8.12) | 2.63 | 1.09 | 10 (6) |
| Costa Rica | 0.70034 | 12.3 (8.76 to 16.77) | 2.73 | 9.57 | 73 (71) |
| Coted'Ivoire | 0.425942 | 70.61 (29.19 to 135.6) | 4.39 | 66.23 | 164 (162) |
| Croatia | 0.798341 | 12.16 (8.2 to 17.6) | 2.64 | 9.52 | 72 (69) |
| Cuba | 0.66873 | 8.9 (6.07 to 12.73) | 2.94 | 5.97 | 35 (33) |
| Cyprus | 0.835631 | 9.79 (6.21 to 14.29) | 2.65 | 7.14 | 46 (42) |
| Czechia | 0.82845 | 16.47 (11.05 to 23.27) | 2.63 | 13.83 | 99 (96) |
| Democratic People's Republic of Korea | 0.569855 | 35.92 (16.54 to 77.27) | 3 | 32.92 | 139 (136) |
| Democratic Republic of the Congo | 0.38318 | 72.78 (32.82 to 189.95) | 4.66 | 68.12 | 165 (165) |
| Denmark | 0.896424 | 8.69 (4.79 to 13.79) | 2.72 | 5.96 | 34 (30) |
| Djibouti | 0.487958 | 76.03 (38.33 to 158.79) | 4.83 | 71.2 | 171 (171) |
| Dominica | 0.746967 | 75.38 (41.14 to 160.85) | 2.91 | 72.46 | 174 (169) |
| Dominican Republic | 0.619388 | 25.31 (13.34 to 50.54) | 2.88 | 22.43 | 121 (119) |
| Ecuador | 0.661017 | 21.41 (16.6 to 27.63) | 2.88 | 18.53 | 113 (110) |
| Egypt | 0.606787 | 14.82 (9.45 to 22.5) | 2.9 | 11.92 | 91 (90) |
| El Salvador | 0.563775 | 30.65 (21.3 to 45.5) | 3.13 | 27.53 | 132 (129) |
| Equatorial Guinea | 0.657857 | 37.05 (13.04 to 98.92) | 2.86 | 34.19 | 142 (140) |
| Eritrea | 0.403864 | 134.32 (64.59 to 289.02) | 4.4 | 129.93 | 199 (198) |
| Estonia | 0.844918 | 11.32 (8.28 to 15.52) | 2.65 | 8.67 | 64 (61) |
| Eswatini | 0.58546 | 95.79 (52.91 to 161.9) | 2.91 | 92.88 | 186 (183) |
| Ethiopia | 0.358823 | 108.07 (65.8 to 178.27) | 4.88 | 103.19 | 194 (191) |
| Fiji | 0.675052 | 16.48 (9.06 to 28.02) | 2.9 | 13.58 | 96 (97) |
| Finland | 0.859831 | 12.09 (8.64 to 16.64) | 2.59 | 9.5 | 71 (67) |
| France | 0.838365 | 9.86 (5.92 to 14.82) | 2.64 | 7.23 | 50 (45) |
| Gabon | 0.634691 | 37.02 (14.27 to 98.26) | 2.88 | 34.14 | 141 (139) |
| Gambia | 0.409714 | 61.15 (29.25 to 145.47) | 4.28 | 56.87 | 158 (156) |
| Georgia | 0.732474 | 13.66 (9.89 to 18.36) | 2.74 | 10.91 | 84 (82) |
| Germany | 0.902957 | 9.79 (6.22 to 14.65) | 2.61 | 7.18 | 49 (43) |
| Ghana | 0.56493 | 59.54 (24.94 to 134.63) | 3.07 | 56.46 | 156 (154) |
| Greece | 0.791854 | 8.87 (5.46 to 13.61) | 2.63 | 6.25 | 39 (32) |
| Greenland | 0.82621 | 24.4 (16.52 to 35.41) | 2.66 | 21.74 | 120 (118) |
| Grenada | 0.668993 | 8.48 (6.14 to 11.57) | 2.93 | 5.56 | 27 (26) |
| Guam | 0.803982 | 6.54 (3.85 to 10.92) | 2.61 | 3.93 | 20 (17) |
| Guatemala | 0.539972 | 33.93 (26.76 to 42.99) | 4.22 | 29.71 | 136 (134) |
| Guinea | 0.336401 | 103.25 (43.93 to 186.95) | 4.73 | 98.53 | 191 (189) |
| Guinea–Bissau | 0.35311 | 76.64 (33.38 to 165.62) | 4.72 | 71.92 | 173 (172) |
| Guyana | 0.650812 | 14.68 (10.84 to 19.25) | 2.88 | 11.8 | 89 (87) |
| Haiti | 0.448278 | 86.01 (35.92 to 171.52) | 4.41 | 81.59 | 179 (178) |
| Honduras | 0.513037 | 31.84 (17.71 to 65.79) | 4.46 | 27.38 | 131 (133) |
| Hungary | 0.790755 | 13.23 (9.04 to 19.42) | 2.74 | 10.49 | 82 (79) |
| Iceland | 0.876362 | 11 (7.15 to 15.91) | 2.69 | 8.31 | 60 (56) |
| India | 0.575402 | 6.42 (2.27 to 11.58) | 3.18 | 3.24 | 16 (15) |
| Indonesia | 0.656868 | 10.05 (2.97 to 15.21) | 2.89 | 7.16 | 48 (48) |
| Iran (Islamic Republic of) | 0.697207 | 17.37 (12.53 to 29.41) | 2.95 | 14.42 | 104 (102) |
| Iraq | 0.662626 | 12.61 (7.77 to 21.31) | 2.88 | 9.72 | 75 (75) |
| Ireland | 0.873754 | 9.41 (5.72 to 14.27) | 2.68 | 6.73 | 44 (38) |
| Israel | 0.809012 | 8.48 (4.93 to 13.14) | 2.63 | 5.86 | 32 (25) |
| Italy | 0.805774 | 9.3 (6.41 to 12.98) | 2.64 | 6.66 | 43 (37) |
| Jamaica | 0.683263 | 8.97 (6.38 to 12.26) | 2.92 | 6.06 | 37 (34) |
| Japan | 0.871242 | 8.2 (5.63 to 11.58) | 2.64 | 5.56 | 26 (22) |
| Jordan | 0.725307 | 30.92 (21.46 to 43.12) | 2.94 | 27.98 | 133 (130) |
| Kazakhstan | 0.725144 | 51.59 (42.01 to 62.96) | 2.92 | 48.67 | 151 (149) |
| Kenya | 0.523768 | 79.9 (30.75 to 163.84) | 4.28 | 75.62 | 177 (175) |
| Kiribati | 0.527187 | 77.49 (33.35 to 162.45) | 4.24 | 73.25 | 175 (173) |
| Kuwait | 0.846651 | 20.18 (16.21 to 24.73) | 2.71 | 17.47 | 111 (108) |
| Kyrgyzstan | 0.603979 | 23.7 (19.98 to 28.37) | 2.95 | 20.75 | 117 (115) |
| Lao People's Democratic Republic | 0.489136 | 10.13 (5.65 to 17.71) | 4.36 | 5.77 | 30 (50) |
| Latvia | 0.830664 | 11.82 (8.64 to 15.87) | 2.84 | 8.98 | 67 (65) |
| Lebanon | 0.744746 | 6.07 (3.91 to 9.32) | 2.73 | 3.34 | 17 (14) |
| Lesotho | 0.510393 | 114.39 (65.94 to 186.03) | 4.24 | 110.15 | 197 (195) |
| Liberia | 0.352442 | 74.5 (28.53 to 176.07) | 4.49 | 70.01 | 169 (166) |
| Libya | 0.725771 | 40 (18.44 to 70.15) | 2.68 | 37.32 | 146 (144) |
| Lithuania | 0.856484 | 16.88 (13.47 to 20.99) | 2.66 | 14.23 | 102 (99) |
| Luxembourg | 0.884429 | 9.04 (5.49 to 13.37) | 2.7 | 6.35 | 40 (35) |
| Madagascar | 0.400247 | 97.7 (50.23 to 167.62) | 4.34 | 93.35 | 187 (184) |
| Malawi | 0.384554 | 86.19 (40.7 to 197.41) | 4.32 | 81.87 | 180 (179) |
| Malaysia | 0.742524 | 5.88 (3.67 to 9.12) | 2.71 | 3.18 | 15 (12) |
| Maldives | 0.650887 | 3.31 (1.86 to 5.93) | 2.87 | 0.44 | 6 (4) |
| Mali | 0.26858 | 99.02 (44.82 to 176.1) | 15.61 | 83.4 | 183 (185) |
| Malta | 0.801585 | 10.24 (6.71 to 15.01) | 2.68 | 7.55 | 57 (53) |
| Marshall Islands | 0.574091 | 25.5 (11.11 to 50.73) | 3.07 | 22.43 | 122 (120) |
| Mauritania | 0.498945 | 39.15 (18.22 to 83.64) | 4.52 | 34.63 | 144 (143) |
| Mauritius | 0.71826 | 3.8 (2.88 to 4.96) | 2.9 | 0.91 | 9 (7) |
| Mexico | 0.664575 | 18.13 (14.19 to 23.05) | 2.95 | 15.18 | 106 (104) |
| Micronesia (Federated States of) | 0.587535 | 22.55 (11.12 to 47.68) | 2.86 | 19.69 | 115 (113) |
| Monaco | 0.908263 | 16.53 (9.9 to 34.71) | 2.59 | 13.94 | 100 (98) |
| Mongolia | 0.617622 | 67.64 (40.9 to 114.32) | 2.92 | 64.73 | 162 (159) |
| Montenegro | 0.795801 | 15.5 (10.15 to 23.03) | 2.62 | 12.87 | 94 (92) |
| Morocco | 0.562698 | 37.66 (16.08 to 84.06) | 3.2 | 34.46 | 143 (141) |
| Mozambique | 0.326463 | 112.92 (53.01 to 272.22) | 4.46 | 108.47 | 196 (194) |
| Myanmar | 0.533901 | 11.61 (5.94 to 28.29) | 4.14 | 7.46 | 54 (62) |
| Namibia | 0.617565 | 72.52 (41.26 to 123.28) | 2.85 | 69.67 | 166 (164) |
| Nauru | 0.625178 | 31.42 (16.01 to 57.27) | 2.9 | 28.52 | 134 (131) |
| Nepal | 0.433175 | 163.5 (87.35 to 296.44) | 4.37 | 159.13 | 200 (199) |
| Netherlands | 0.888464 | 8.43 (4.94 to 13.06) | 2.64 | 5.79 | 31 (23) |
| New Zealand | 0.849442 | 13.07 (8.75 to 18.49) | 2.65 | 10.43 | 81 (78) |
| Nicaragua | 0.523958 | 14.6 (9.6 to 21.31) | 4.74 | 9.86 | 77 (86) |
| Niger | 0.168073 | 111.32 (45.25 to 243.8) | 111.32 | 0 | 1.5 (193) |
| Nigeria | 0.503391 | 86.89 (58.01 to 130.36) | 4.4 | 82.48 | 181 (180) |
| Niue | 0.726222 | 72.5 (35.47 to 138.71) | 2.68 | 69.82 | 167 (163) |
| North Macedonia | 0.75063 | 14.09 (9.47 to 20.44) | 2.76 | 11.33 | 86 (83) |
| Northern Mariana Islands | 0.771535 | 13.63 (6.41 to 23.7) | 2.66 | 10.97 | 85 (81) |
| Norway | 0.916133 | 38.65 (26.01 to 54.23) | 2.64 | 36.01 | 145 (142) |
| Oman | 0.773392 | 2.67 (1.76 to 3.92) | 2.62 | 0.05 | 3 (1) |
| Pakistan | 0.504029 | 8.55 (4.04 to 26.31) | 4.74 | 3.81 | 18 (28) |
| Palau | 0.754047 | 48.69 (26.06 to 88.99) | 2.74 | 45.95 | 150 (148) |
| Palestine | 0.631012 | 9.5 (5.96 to 15.22) | 2.89 | 6.61 | 42 (40) |
| Panama | 0.708865 | 14.82 (10.89 to 19.93) | 2.68 | 12.13 | 92 (91) |
| Papua New Guinea | 0.417797 | 54.42 (24.61 to 105.48) | 4.36 | 50.06 | 152 (150) |
| Paraguay | 0.635718 | 12.21 (7.89 to 19.65) | 2.87 | 9.34 | 70 (70) |
| Peru | 0.662054 | 17.14 (10.64 to 26.97) | 2.88 | 14.26 | 103 (101) |
| Philippines | 0.651219 | 4.71 (3.34 to 6.07) | 2.89 | 1.82 | 12 (9) |
| Poland | 0.812043 | 10.22 (7.88 to 13.27) | 2.72 | 7.5 | 55 (52) |
| Portugal | 0.744152 | 14.24 (10.27 to 19.92) | 2.91 | 11.34 | 87 (85) |
| Puerto Rico | 0.825526 | 5.35 (3.24 to 8.12) | 2.65 | 2.7 | 14 (11) |
| Qatar | 0.846861 | 11.3 (6.89 to 18.85) | 2.63 | 8.67 | 65 (60) |
| Republic of Korea | 0.886675 | 10.11 (6.35 to 14.82) | 2.69 | 7.42 | 53 (49) |
| Republic of Moldova | 0.732215 | 62.33 (48.56 to 80.76) | 2.93 | 59.41 | 159 (157) |
| Romania | 0.768454 | 31.79 (25.47 to 39.37) | 2.69 | 29.11 | 135 (132) |
| Russian Federation | 0.808536 | 58.52 (52.49 to 63.55) | 2.75 | 55.77 | 155 (153) |
| Rwanda | 0.435589 | 75.31 (35.61 to 182.4) | 4.64 | 70.68 | 170 (168) |
| Saint Kitts and Nevis | 0.754987 | 10.58 (7.46 to 14.54) | 2.8 | 7.78 | 58 (54) |
| Saint Lucia | 0.67251 | 12.07 (8.75 to 16.51) | 2.88 | 9.2 | 69 (66) |
| Saint Vincent and the Grenadines | 0.637196 | 8.62 (6.29 to 11.8) | 2.88 | 5.74 | 28 (29) |
| Samoa | 0.593393 | 17 (8.34 to 34.19) | 2.88 | 14.12 | 101 (100) |
| San Marino | 0.888005 | 14.78 (9.48 to 21.98) | 2.61 | 12.17 | 93 (89) |
| Sao Tome and Principe | 0.505414 | 21.88 (7.81 to 70.02) | 4.46 | 17.42 | 110 (111) |
| Saudi Arabia | 0.815143 | 9.47 (5.3 to 16.81) | 2.73 | 6.74 | 45 (39) |
| Senegal | 0.408054 | 58.21 (25.45 to 136.44) | 4.26 | 53.96 | 154 (152) |
| Serbia | 0.792416 | 14.13 (9.21 to 21.05) | 2.62 | 11.51 | 88 (84) |
| Seychelles | 0.730151 | 5.32 (3.71 to 7.67) | 2.76 | 2.56 | 13 (10) |
| Sierra Leone | 0.358666 | 109.23 (48.73 to 197.91) | 4.73 | 104.5 | 195 (192) |
| Singapore | 0.856098 | 8.7 (5 to 13.83) | 2.71 | 5.99 | 36 (31) |
| Slovakia | 0.810611 | 16.36 (11.19 to 23.76) | 2.6 | 13.76 | 98 (94) |
| Slovenia | 0.842431 | 12.96 (8.04 to 19.64) | 2.61 | 10.35 | 80 (77) |
| Solomon Islands | 0.42936 | 36.62 (15.65 to 89.42) | 4.36 | 32.26 | 138 (138) |
| Somalia | 0.077688 | 174.38 (78.29 to 374.37) | 174.38 | 0 | 1.5 (202) |
| South Africa | 0.679627 | 68.05 (48.36 to 88.26) | 2.88 | 65.18 | 163 (161) |
| South Sudan | 0.278371 | 239.6 (125.16 to 413.09) | 9.24 | 230.36 | 204 (204) |
| Spain | 0.769284 | 9.91 (6.26 to 14.88) | 2.66 | 7.25 | 51 (46) |
| Sri Lanka | 0.701535 | 2.94 (1.96 to 4.43) | 2.73 | 0.21 | 4 (2) |
| Sudan | 0.54195 | 82.5 (37.32 to 147.6) | 4.42 | 78.08 | 178 (176) |
| Suriname | 0.633666 | 19.45 (10.93 to 37.55) | 2.91 | 16.54 | 109 (107) |
| Sweden | 0.88688 | 12.31 (8.04 to 18.47) | 2.72 | 9.6 | 74 (72) |
| Switzerland | 0.933059 | 8.06 (4.66 to 12.41) | 2.81 | 5.25 | 23 (21) |
| Syrian Arab Republic | 0.623004 | 19.2 (12.33 to 28.38) | 2.92 | 16.29 | 107 (105) |
| Taiwan (Province of China) | 0.874747 | 10.95 (8.92 to 13.06) | 2.63 | 8.32 | 61 (55) |
| Tajikistan | 0.541511 | 55.7 (29.51 to 122.13) | 4.03 | 51.67 | 153 (151) |
| Thailand | 0.682548 | 3.82 (2.46 to 5.81) | 3.04 | 0.79 | 8 (8) |
| Timor–Leste | 0.444668 | 11.07 (6.26 to 20.3) | 4.53 | 6.54 | 41 (57) |
| Togo | 0.408534 | 61.14 (24.9 to 134.43) | 4.34 | 56.8 | 157 (155) |
| Tokelau | 0.686426 | 88.64 (40.95 to 214.37) | 2.93 | 85.71 | 184 (181) |
| Tonga | 0.62635 | 62.99 (36.23 to 108.67) | 2.9 | 60.09 | 160 (158) |
| Trinidad and Tobago | 0.768763 | 9.07 (6.46 to 12.6) | 2.84 | 6.23 | 38 (36) |
| Tunisia | 0.682432 | 20.58 (10.02 to 43.1) | 2.98 | 17.6 | 112 (109) |
| Turkey | 0.712693 | 19.24 (12.65 to 29.35) | 2.91 | 16.33 | 108 (106) |
| Turkmenistan | 0.682161 | 14.71 (11.55 to 18.8) | 2.86 | 11.85 | 90 (88) |
| Tuvalu | 0.576621 | 24.25 (11.11 to 51.1) | 3.08 | 21.17 | 118 (117) |
| Uganda | 0.423261 | 94.92 (43.15 to 199.84) | 4.36 | 90.56 | 185 (182) |
| Ukraine | 0.760774 | 29.07 (22.6 to 35.8) | 2.77 | 26.31 | 129 (126) |
| United Arab Emirates | 0.849318 | 6.51 (4.24 to 9.88) | 2.64 | 3.87 | 19 (16) |
| United Kingdom | 0.859 | 10 (6.87 to 13.96) | 2.7 | 7.31 | 52 (47) |
| United Republic of Tanzania | 0.446568 | 102.51 (47.2 to 229.22) | 4.58 | 97.93 | 190 (188) |
| United States Virgin Islands | 0.821831 | 11.11 (6.01 to 19.02) | 2.69 | 8.42 | 62 (58) |
| United States of America | 0.862448 | 13.53 (11 to 16.66) | 2.71 | 10.82 | 83 (80) |
| Uruguay | 0.719283 | 28.87 (21.62 to 38.32) | 2.89 | 25.98 | 128 (124) |
| Uzbekistan | 0.662622 | 29.3 (23.98 to 35.83) | 2.91 | 26.39 | 130 (128) |
| Vanuatu | 0.473101 | 29.14 (14.03 to 58.79) | 4.7 | 24.44 | 125 (127) |
| Venezuela (Bolivarian Republic of) | 0.596513 | 22.48 (16.94 to 29.54) | 2.87 | 19.61 | 114 (112) |
| Viet Nam | 0.627934 | 3.21 (1.67 to 7.32) | 2.85 | 0.36 | 5 (3) |
| Yemen | 0.450376 | 75.99 (34.29 to 152.52) | 4.73 | 71.26 | 172 (170) |
| Zambia | 0.505949 | 79.31 (42.08 to 163.15) | 4.33 | 74.99 | 176 (174) |
| Zimbabwe | 0.473819 | 197.91 (94.24 to 341.39) | 4.32 | 193.58 | 203 (203) |

**Supplementary Table 8.** Frontier of incidence–related Burden of Childhood Poisoning and Effective Difference by Country or Territory

| Location | SDI | Rate of Incidence | Frontier Incidence | Effective difference | Effective difference rank (Age–standardized Incidence rank) |
| --- | --- | --- | --- | --- | --- |
| Afghanistan | 0.3372 | 18.15 (10.83 to 29.03) | 15.29 | 2.86 | 8 (14) |
| Albania | 0.70685 | 236.86 (144.93 to 363.4) | 10.89 | 225.97 | 191 (191) |
| Algeria | 0.659501 | 23.47 (13.97 to 36.91) | 10.89 | 12.58 | 52 (41) |
| American Samoa | 0.723728 | 22.19 (12.37 to 36.5) | 10.85 | 11.34 | 46 (34) |
| Andorra | 0.869444 | 122.22 (73.64 to 195.35) | 10.89 | 111.33 | 148 (148) |
| Angola | 0.453722 | 20.13 (12.19 to 31.58) | 12.48 | 7.65 | 21 (24) |
| Antigua and Barbuda | 0.749887 | 88.9 (51.29 to 141.53) | 10.85 | 78.05 | 123 (123) |
| Argentina | 0.723123 | 340.07 (216.08 to 516.46) | 10.9 | 329.17 | 203 (203) |
| Armenia | 0.701833 | 105.93 (57.59 to 178.8) | 10.83 | 95.1 | 133 (132) |
| Australia | 0.844253 | 130.65 (75.96 to 211.94) | 10.89 | 119.76 | 158 (158) |
| Austria | 0.853837 | 149.05 (92.68 to 231.35) | 10.92 | 138.13 | 176 (176) |
| Azerbaijan | 0.694851 | 105.87 (59.41 to 168.9) | 10.89 | 94.98 | 132 (131) |
| Bahamas | 0.805021 | 81 (44.02 to 135.97) | 10.91 | 70.1 | 117 (117) |
| Bahrain | 0.753043 | 31.45 (17.22 to 51.8) | 10.91 | 20.54 | 104 (98) |
| Bangladesh | 0.492421 | 12.23 (7.1 to 19.43) | 12.18 | 0.05 | 2 (2) |
| Barbados | 0.746749 | 90.39 (49.56 to 151.63) | 10.9 | 79.5 | 125 (125) |
| Belarus | 0.784485 | 135.86 (80.06 to 213.11) | 10.83 | 125.03 | 167 (166) |
| Belgium | 0.853654 | 152.11 (94.1 to 238.65) | 10.91 | 141.2 | 178 (178) |
| Belize | 0.610229 | 75.03 (42.48 to 122.95) | 10.89 | 64.14 | 114 (114) |
| Benin | 0.373487 | 32.24 (19.64 to 49.11) | 15.08 | 17.16 | 93 (100) |
| Bermuda | 0.821365 | 99.41 (54.89 to 161.33) | 10.82 | 88.59 | 129 (129) |
| Bhutan | 0.473062 | 13.17 (7.4 to 21.8) | 12.34 | 0.82 | 5 (3) |
| Bolivia (Plurinational State of) | 0.599011 | 64.4 (37.68 to 102.06) | 10.91 | 53.49 | 109 (109) |
| Bosnia and Herzegovina | 0.723078 | 277.92 (156.22 to 449.02) | 10.9 | 267.01 | 199 (199) |
| Botswana | 0.642722 | 37.14 (23.99 to 55.37) | 10.86 | 26.28 | 106 (106) |
| Brazil | 0.653044 | 29.02 (16.77 to 46.77) | 10.84 | 18.18 | 97 (85) |
| Brunei Darussalam | 0.810234 | 130.04 (76.9 to 207.55) | 10.91 | 119.14 | 156 (156) |
| Bulgaria | 0.768151 | 235.89 (137.29 to 377.94) | 10.91 | 224.99 | 189 (189) |
| Burkina Faso | 0.285118 | 32.87 (20.03 to 49.38) | 16.02 | 16.86 | 89 (101) |
| Burundi | 0.289374 | 29.1 (18.27 to 45.09) | 16.09 | 13.01 | 60 (87) |
| Cabo Verde | 0.533535 | 28.91 (15.65 to 48.03) | 12.14 | 16.78 | 88 (84) |
| Cambodia | 0.473621 | 15.89 (8.73 to 25.86) | 12.39 | 3.5 | 9 (5) |
| Cameroon | 0.479691 | 27.17 (16.87 to 41.46) | 12.39 | 14.79 | 76 (72) |
| Canada | 0.873171 | 109.27 (65.39 to 171.56) | 10.92 | 98.35 | 136 (136) |
| Central African Republic | 0.309168 | 23.42 (14.43 to 34.94) | 15.88 | 7.53 | 18 (40) |
| Chad | 0.240436 | 32.03 (20.55 to 48.01) | 17.43 | 14.6 | 73 (99) |
| Chile | 0.771515 | 313.17 (200.69 to 458.61) | 10.91 | 302.26 | 201 (201) |
| China | 0.72163 | 25.41 (15.63 to 39.44) | 10.84 | 14.57 | 72 (56) |
| Colombia | 0.655443 | 130.56 (76.3 to 216.96) | 10.84 | 119.72 | 157 (157) |
| Comoros | 0.475979 | 28.34 (17.21 to 43.81) | 12.39 | 15.95 | 83 (81) |
| Congo | 0.583075 | 18.02 (10.42 to 29.39) | 10.9 | 7.12 | 17 (13) |
| Cook Islands | 0.77911 | 26.83 (14.37 to 45.9) | 10.88 | 15.95 | 84 (69) |
| Costa Rica | 0.70034 | 152.18 (88.95 to 247.56) | 10.89 | 141.28 | 179 (179) |
| Coted'Ivoire | 0.425942 | 29.32 (18.2 to 44.73) | 14.65 | 14.67 | 74 (89) |
| Croatia | 0.798341 | 236.75 (139.36 to 374.92) | 10.96 | 225.8 | 190 (190) |
| Cuba | 0.66873 | 137.02 (76.71 to 225.18) | 10.93 | 126.09 | 169 (169) |
| Cyprus | 0.835631 | 120.31 (72.47 to 190.41) | 10.88 | 109.43 | 146 (146) |
| Czechia | 0.82845 | 317.93 (177.68 to 512.84) | 10.89 | 307.04 | 202 (202) |
| Democratic People's Republic of Korea | 0.569855 | 17.84 (10.41 to 28.16) | 10.94 | 6.9 | 14 (10) |
| Democratic Republic of the Congo | 0.38318 | 18.77 (11.35 to 28.83) | 15.03 | 3.74 | 11 (16) |
| Denmark | 0.896424 | 128.52 (77.75 to 204.55) | 10.89 | 117.63 | 153 (153) |
| Djibouti | 0.487958 | 29.32 (17.65 to 45.15) | 12.38 | 16.93 | 91 (88) |
| Dominica | 0.746967 | 112.42 (69.59 to 166.97) | 10.84 | 101.58 | 141 (141) |
| Dominican Republic | 0.619388 | 78.24 (46.4 to 126.38) | 10.89 | 67.35 | 115 (115) |
| Ecuador | 0.661017 | 71.58 (40.76 to 115) | 10.89 | 60.69 | 111 (111) |
| Egypt | 0.606787 | 21.4 (11.39 to 35.58) | 10.96 | 10.45 | 39 (29) |
| El Salvador | 0.563775 | 135.1 (76.06 to 220.33) | 11.69 | 123.41 | 164 (165) |
| Equatorial Guinea | 0.657857 | 17.64 (9.95 to 29.11) | 10.88 | 6.75 | 13 (9) |
| Eritrea | 0.403864 | 30.05 (18.72 to 46.4) | 14.68 | 15.37 | 79 (91) |
| Estonia | 0.844918 | 175.9 (96.47 to 289.72) | 10.91 | 164.99 | 188 (188) |
| Eswatini | 0.58546 | 30.26 (19.5 to 44.68) | 10.85 | 19.4 | 102 (93) |
| Ethiopia | 0.358823 | 25.69 (16.01 to 39.72) | 15.15 | 10.55 | 41 (60) |
| Fiji | 0.675052 | 18.82 (10.52 to 31.18) | 10.91 | 7.91 | 24 (17) |
| Finland | 0.859831 | 112.03 (67.03 to 178.58) | 10.9 | 101.13 | 138 (138) |
| France | 0.838365 | 132.68 (81.14 to 207.23) | 10.91 | 121.77 | 159 (159) |
| Gabon | 0.634691 | 19.62 (10.93 to 32.24) | 10.92 | 8.7 | 28 (19) |
| Gambia | 0.409714 | 25.83 (15.54 to 40.2) | 13.96 | 11.87 | 50 (61) |
| Georgia | 0.732474 | 174.65 (105.98 to 270.75) | 10.89 | 163.76 | 187 (187) |
| Germany | 0.902957 | 124.24 (73.87 to 198.82) | 10.82 | 113.41 | 151 (151) |
| Ghana | 0.56493 | 27.39 (16.84 to 42.4) | 11.9 | 15.48 | 80 (74) |
| Greece | 0.791854 | 116.85 (71 to 185.74) | 10.89 | 105.96 | 143 (143) |
| Greenland | 0.82621 | 91.11 (56.78 to 139.41) | 10.9 | 80.2 | 126 (126) |
| Grenada | 0.668993 | 83.19 (46.26 to 135.65) | 10.91 | 72.28 | 120 (119) |
| Guam | 0.803982 | 24.91 (13.66 to 42.2) | 10.91 | 14 | 65 (52) |
| Guatemala | 0.539972 | 148.41 (93.08 to 223.57) | 12.23 | 136.18 | 175 (175) |
| Guinea | 0.336401 | 33.87 (21.5 to 51.92) | 15.29 | 18.58 | 100 (103) |
| Guinea–Bissau | 0.35311 | 25.47 (16.07 to 39.15) | 15.18 | 10.29 | 37 (59) |
| Guyana | 0.650812 | 74.28 (41.95 to 122.11) | 10.88 | 63.4 | 113 (113) |
| Haiti | 0.448278 | 85 (54.24 to 125.59) | 12.88 | 72.12 | 119 (122) |
| Honduras | 0.513037 | 140.53 (83.29 to 222.86) | 12.27 | 128.26 | 170 (172) |
| Hungary | 0.790755 | 266.16 (154.55 to 432.39) | 10.84 | 255.32 | 197 (197) |
| Iceland | 0.876362 | 133.66 (81.57 to 207.86) | 10.84 | 122.82 | 161 (161) |
| India | 0.575402 | 10.85 (6.15 to 17.91) | 10.84 | 0.01 | 1 (1) |
| Indonesia | 0.656868 | 18.76 (10.41 to 31.3) | 10.9 | 7.86 | 23 (15) |
| Iran (Islamic Republic of) | 0.697207 | 17.9 (10.37 to 28.59) | 10.91 | 6.99 | 15 (11) |
| Iraq | 0.662626 | 22.63 (12.32 to 37.71) | 10.88 | 11.75 | 49 (37) |
| Ireland | 0.873754 | 133.29 (80.72 to 211.8) | 10.91 | 122.38 | 160 (160) |
| Israel | 0.809012 | 121.28 (73.79 to 193.31) | 10.93 | 110.35 | 147 (147) |
| Italy | 0.805774 | 134.05 (88.21 to 197.8) | 10.85 | 123.2 | 162 (162) |
| Jamaica | 0.683263 | 94.51 (51.56 to 154.88) | 10.92 | 83.59 | 127 (127) |
| Japan | 0.871242 | 139.37 (80.68 to 224.92) | 10.85 | 128.51 | 172 (171) |
| Jordan | 0.725307 | 25.41 (15.01 to 40.55) | 10.86 | 14.55 | 71 (57) |
| Kazakhstan | 0.725144 | 134.28 (84.99 to 200.19) | 10.89 | 123.39 | 163 (163) |
| Kenya | 0.523768 | 21.44 (12.48 to 34.24) | 11.99 | 9.44 | 33 (30) |
| Kiribati | 0.527187 | 26.39 (17.38 to 38.8) | 12.08 | 14.31 | 70 (65) |
| Kuwait | 0.846651 | 27.93 (15.76 to 45.61) | 10.93 | 17 | 92 (78) |
| Kyrgyzstan | 0.603979 | 106.03 (61.31 to 167.51) | 10.89 | 95.13 | 134 (133) |
| Lao People's Democratic Republic | 0.489136 | 16.07 (9.07 to 26.09) | 12.4 | 3.68 | 10 (6) |
| Latvia | 0.830664 | 160.93 (87.18 to 267.75) | 10.89 | 150.04 | 182 (182) |
| Lebanon | 0.744746 | 27.46 (15.58 to 44.7) | 10.87 | 16.59 | 87 (76) |
| Lesotho | 0.510393 | 30.57 (19.99 to 45.28) | 12.32 | 18.24 | 98 (95) |
| Liberia | 0.352442 | 23.52 (14.28 to 36.8) | 15.25 | 8.26 | 26 (42) |
| Libya | 0.725771 | 24.39 (14.06 to 38.78) | 10.89 | 13.5 | 62 (48) |
| Lithuania | 0.856484 | 139.22 (79.63 to 219.68) | 10.88 | 128.35 | 171 (170) |
| Luxembourg | 0.884429 | 123.37 (73.15 to 196.78) | 10.86 | 112.51 | 150 (150) |
| Madagascar | 0.400247 | 25.42 (15.08 to 39.5) | 14.91 | 10.51 | 40 (58) |
| Malawi | 0.384554 | 26 (15.89 to 39.81) | 14.92 | 11.08 | 43 (63) |
| Malaysia | 0.742524 | 22.52 (11.85 to 37.78) | 10.89 | 11.63 | 47 (36) |
| Maldives | 0.650887 | 24.59 (12.87 to 42.5) | 10.84 | 13.75 | 64 (50) |
| Mali | 0.26858 | 35.22 (21.68 to 53.44) | 16.14 | 19.08 | 101 (104) |
| Malta | 0.801585 | 129.2 (77.06 to 205.8) | 10.87 | 118.33 | 154 (154) |
| Marshall Islands | 0.574091 | 19.94 (11.88 to 31.54) | 10.91 | 9.03 | 31 (22) |
| Mauritania | 0.498945 | 23.98 (13.75 to 38.83) | 12.25 | 11.73 | 48 (46) |
| Mauritius | 0.71826 | 24.44 (13.92 to 39.6) | 10.96 | 13.49 | 61 (49) |
| Mexico | 0.664575 | 112.24 (66.02 to 179.73) | 10.85 | 101.39 | 139 (139) |
| Micronesia (Federated States of) | 0.587535 | 19.71 (11.55 to 31.06) | 10.83 | 8.88 | 29 (20) |
| Monaco | 0.908263 | 136.94 (83.11 to 216.58) | 10.91 | 126.03 | 168 (168) |
| Mongolia | 0.617622 | 119.26 (74.14 to 178.25) | 10.88 | 108.38 | 144 (144) |
| Montenegro | 0.795801 | 255.6 (145.81 to 411.17) | 10.88 | 244.72 | 193 (193) |
| Morocco | 0.562698 | 22.31 (12.84 to 35.59) | 11.03 | 11.28 | 44 (35) |
| Mozambique | 0.326463 | 24.24 (14.75 to 37.11) | 15.93 | 8.31 | 27 (47) |
| Myanmar | 0.533901 | 41.01 (27.14 to 60.61) | 12.08 | 28.93 | 108 (108) |
| Namibia | 0.617565 | 27.74 (17.41 to 42.69) | 10.86 | 16.88 | 90 (77) |
| Nauru | 0.625178 | 20.47 (12.58 to 31.93) | 11.42 | 9.05 | 32 (25) |
| Nepal | 0.433175 | 20.74 (13.01 to 31.04) | 13.17 | 7.57 | 19 (27) |
| Netherlands | 0.888464 | 119.46 (71.94 to 190.29) | 10.86 | 108.6 | 145 (145) |
| New Zealand | 0.849442 | 167.45 (109.31 to 246.64) | 10.92 | 156.53 | 184 (184) |
| Nicaragua | 0.523958 | 135.91 (80.52 to 214.08) | 12.1 | 123.8 | 165 (167) |
| Niger | 0.168073 | 31.22 (18.54 to 48.21) | 30.9 | 0.32 | 4 (97) |
| Nigeria | 0.503391 | 24.93 (15.19 to 38.59) | 12.34 | 12.59 | 53 (53) |
| Niue | 0.726222 | 22.93 (13.77 to 36.96) | 10.89 | 12.04 | 51 (39) |
| North Macedonia | 0.75063 | 244.92 (143.97 to 395.41) | 10.85 | 234.07 | 192 (192) |
| Northern Mariana Islands | 0.771535 | 24.95 (13.36 to 41.98) | 10.9 | 14.04 | 68 (54) |
| Norway | 0.916133 | 510.67 (357.14 to 707.34) | 10.89 | 499.79 | 204 (204) |
| Oman | 0.773392 | 26.48 (14.64 to 43.57) | 10.91 | 15.57 | 81 (67) |
| Pakistan | 0.504029 | 14.75 (9.05 to 22.6) | 12.37 | 2.38 | 6 (4) |
| Palau | 0.754047 | 39.59 (26.58 to 57.28) | 10.91 | 28.68 | 107 (107) |
| Palestine | 0.631012 | 27.18 (14.21 to 47.49) | 11.02 | 16.16 | 86 (73) |
| Panama | 0.708865 | 141.25 (83.93 to 223.78) | 10.9 | 130.35 | 173 (173) |
| Papua New Guinea | 0.417797 | 21.61 (13.55 to 32.27) | 13.98 | 7.63 | 20 (32) |
| Paraguay | 0.635718 | 36.98 (20.39 to 60.24) | 10.84 | 26.14 | 105 (105) |
| Peru | 0.662054 | 69.98 (40.67 to 111) | 10.93 | 59.05 | 110 (110) |
| Philippines | 0.651219 | 19.73 (10.78 to 32.89) | 10.84 | 8.89 | 30 (21) |
| Poland | 0.812043 | 170.94 (96.93 to 277.75) | 10.92 | 160.02 | 185 (185) |
| Portugal | 0.744152 | 134.76 (82.99 to 209.2) | 10.91 | 123.85 | 166 (164) |
| Puerto Rico | 0.825526 | 106.17 (56.72 to 175.59) | 10.84 | 95.33 | 135 (134) |
| Qatar | 0.846861 | 28.16 (15.54 to 47.57) | 10.89 | 17.27 | 94 (80) |
| Republic of Korea | 0.886675 | 124.78 (72.67 to 200.33) | 10.89 | 113.89 | 152 (152) |
| Republic of Moldova | 0.732215 | 163.4 (110.62 to 236.19) | 10.92 | 152.48 | 183 (183) |
| Romania | 0.768454 | 255.85 (159.99 to 382.77) | 10.84 | 245.01 | 194 (194) |
| Russian Federation | 0.808536 | 115.47 (75.58 to 167.24) | 10.89 | 104.58 | 142 (142) |
| Rwanda | 0.435589 | 26.93 (16.08 to 41.65) | 14.11 | 12.82 | 59 (70) |
| Saint Kitts and Nevis | 0.754987 | 83.46 (46.09 to 137.43) | 10.91 | 72.55 | 121 (120) |
| Saint Lucia | 0.67251 | 84.8 (46.29 to 142.54) | 10.89 | 73.91 | 122 (121) |
| Saint Vincent and the Grenadines | 0.637196 | 81.1 (43.94 to 136.43) | 10.82 | 70.27 | 118 (118) |
| Samoa | 0.593393 | 20.52 (11.66 to 33.33) | 10.89 | 9.62 | 34 (26) |
| San Marino | 0.888005 | 157.92 (99.73 to 244.39) | 10.84 | 147.08 | 181 (181) |
| Sao Tome and Principe | 0.505414 | 27.95 (15.72 to 46.08) | 12.12 | 15.83 | 82 (79) |
| Saudi Arabia | 0.815143 | 23.62 (12.72 to 39.67) | 10.91 | 12.71 | 56 (44) |
| Senegal | 0.408054 | 26.62 (16.44 to 40.84) | 13.94 | 12.69 | 55 (68) |
| Serbia | 0.792416 | 268.79 (152.09 to 434.48) | 10.86 | 257.93 | 198 (198) |
| Seychelles | 0.730151 | 23.73 (12.95 to 39.53) | 10.92 | 12.81 | 58 (45) |
| Sierra Leone | 0.358666 | 30.08 (18.61 to 45.24) | 15.31 | 14.78 | 75 (92) |
| Singapore | 0.856098 | 151.32 (90.75 to 238.13) | 10.92 | 140.4 | 177 (177) |
| Slovakia | 0.810611 | 256.8 (148 to 409.01) | 10.88 | 245.92 | 195 (195) |
| Slovenia | 0.842431 | 298.91 (169.83 to 481.6) | 10.92 | 288 | 200 (200) |
| Solomon Islands | 0.42936 | 29.04 (18.61 to 43.39) | 13.87 | 15.17 | 78 (86) |
| Somalia | 0.077688 | 30.9 (19.53 to 46.79) | 30.81 | 0.08 | 3 (96) |
| South Africa | 0.679627 | 21.55 (13.11 to 33.41) | 10.84 | 10.71 | 42 (31) |
| South Sudan | 0.278371 | 28.83 (17.49 to 44.47) | 16.02 | 12.81 | 57 (83) |
| Spain | 0.769284 | 129.47 (78.29 to 204.43) | 10.85 | 118.62 | 155 (155) |
| Sri Lanka | 0.701535 | 21.12 (11.31 to 35.99) | 10.9 | 10.23 | 36 (28) |
| Sudan | 0.54195 | 26.46 (16.16 to 41.2) | 12.2 | 14.26 | 69 (66) |
| Suriname | 0.633666 | 72.56 (40.43 to 119.01) | 10.87 | 61.69 | 112 (112) |
| Sweden | 0.88688 | 172.47 (105.91 to 269.21) | 10.92 | 161.55 | 186 (186) |
| Switzerland | 0.933059 | 122.6 (74.13 to 196.05) | 10.84 | 111.77 | 149 (149) |
| Syrian Arab Republic | 0.623004 | 23.58 (14.03 to 37.09) | 10.91 | 12.67 | 54 (43) |
| Taiwan (Province of China) | 0.874747 | 26.94 (14.18 to 46.26) | 10.88 | 16.06 | 85 (71) |
| Tajikistan | 0.541511 | 106.23 (58.12 to 176.1) | 11.96 | 94.27 | 130 (135) |
| Thailand | 0.682548 | 17.92 (9.48 to 30.39) | 10.89 | 7.03 | 16 (12) |
| Timor–Leste | 0.444668 | 16.27 (8.84 to 27.03) | 13.56 | 2.71 | 7 (7) |
| Togo | 0.408534 | 25.15 (15.16 to 38.86) | 14.84 | 10.31 | 38 (55) |
| Tokelau | 0.686426 | 22.11 (12.47 to 36.68) | 10.82 | 11.29 | 45 (33) |
| Tonga | 0.62635 | 28.64 (18.5 to 43.27) | 10.93 | 17.71 | 95 (82) |
| Trinidad and Tobago | 0.768763 | 79.12 (43.21 to 133.81) | 10.85 | 68.27 | 116 (116) |
| Tunisia | 0.682432 | 24.83 (14.29 to 39.86) | 10.82 | 14.01 | 67 (51) |
| Turkey | 0.712693 | 25.87 (14.81 to 41.27) | 10.85 | 15.02 | 77 (62) |
| Turkmenistan | 0.682161 | 110.12 (64.83 to 171.92) | 10.85 | 99.28 | 137 (137) |
| Tuvalu | 0.576621 | 17.59 (10.03 to 29) | 10.99 | 6.6 | 12 (8) |
| Uganda | 0.423261 | 27.4 (16.63 to 42.4) | 13.87 | 13.53 | 63 (75) |
| Ukraine | 0.760774 | 90.08 (54.75 to 137.2) | 10.92 | 79.15 | 124 (124) |
| United Arab Emirates | 0.849318 | 29.45 (15.87 to 50.71) | 10.94 | 18.52 | 99 (90) |
| United Kingdom | 0.859 | 144.01 (87.26 to 223.79) | 10.84 | 133.16 | 174 (174) |
| United Republic of Tanzania | 0.446568 | 33.4 (20.27 to 51.86) | 13.51 | 19.89 | 103 (102) |
| United States Virgin Islands | 0.821831 | 98.67 (54.8 to 161.51) | 10.89 | 87.78 | 128 (128) |
| United States of America | 0.862448 | 112.29 (67.94 to 176.66) | 10.89 | 101.4 | 140 (140) |
| Uruguay | 0.719283 | 262.96 (156.62 to 416.75) | 10.91 | 252.05 | 196 (196) |
| Uzbekistan | 0.662622 | 105.64 (61.96 to 167.09) | 10.85 | 94.79 | 131 (130) |
| Vanuatu | 0.473101 | 20.13 (12.02 to 31.58) | 12.35 | 7.78 | 22 (23) |
| Venezuela (Bolivarian Republic of) | 0.596513 | 156.17 (93.56 to 247.63) | 10.89 | 145.28 | 180 (180) |
| Viet Nam | 0.627934 | 18.83 (10.53 to 31.37) | 10.89 | 7.94 | 25 (18) |
| Yemen | 0.450376 | 22.77 (13.99 to 34.53) | 12.59 | 10.18 | 35 (38) |
| Zambia | 0.505949 | 26.15 (15.95 to 40.94) | 12.15 | 14 | 66 (64) |
| Zimbabwe | 0.473819 | 30.44 (19.34 to 45.32) | 12.39 | 18.04 | 96 (94) |
